# Synthesis of 1D Bi_2_O_3_ nanostructures from hybrid electrospun fibrous mats and their morphology, structure, optical and electrical properties

**DOI:** 10.1038/s41598-022-07830-z

**Published:** 2022-03-08

**Authors:** Wiktor Matysiak

**Affiliations:** Department of Engineering Materials and Biomaterials, Silesian University of Technology, Konarskiego 18A, 44-100 Gliwice, Poland

**Keywords:** Materials science, Nanoscience and technology

## Abstract

The aim of this study was to produce Bi_2_O_3_ nanowires using a combination of sol–gel process and electrospinning methods and a solution based on a 13% solution of polyacrylonitrile (PAN) in *N,N*-dimethylformamide (DMF) containing 1.5 g of bismuth (III) nitrate pentahydrate (Bi(NO_3_)_3_·5H_2_O). The obtained fibrous composite mats were dried at room temperature for 24 h followed by the calcination process in air at two different temperatures of 400 °C and 600 °C. Analysis of the morphology of the fabricated Bi_2_O_3_ nanomaterials based on TEM images showed that the obtained ceramic structures could be classified as one-dimensional Bi_2_O_3_ nanostructures, with the sizes of the presented structures being 260 nm, 125 nm and 200 nm for diameter, and 5.5μm , 2 μm and 2.125 μm for length, respectively. Moreover, further analysis of the morphology of the obtained Bi2O3 nanostructures with the use of SEM showed that their diameters ranged from 150 to 500 nm when a calcination temperature of 400 °C was employed, while Bi_2_O_3_ nanowires with diameters ranging from 150 to 450 nm were obtained at 600 °C. To analyse the chemical composition and oscillatory transitions of atoms vibrating between the oscillatory levels in the molecules of the produced 1D nanostructures, and to determine the functional groups existing therein, EDX and FTIR were used. Transmission peaks in FTIR spectra recorded for wave numbers in the range of 400–4000 cm^-1^ were due to the presence of vibrations in Bi–O bonds, which correspond to the structure of Bi_2_O_3_. In addition, a detailed analysis of optical constants of one-dimensional Bi_2_O_3_ nanostructures fabricated using a combination of sol–gel process, electrospinning and calcination methods has been presented in this paper for the first time. Optical studies based on the recorded UV–Vis spectra showed that the obtained Bi_2_O_3_ nanowires were characterized by sharp absorption edges of radiation in the near-ultraviolet range, with sharp absorption edges falling at wavelengths of 400 nm, regardless of the applied temperature during the calcination process. The study of optical constants showed that the Bi_2_O_3_ nanostructures exhibited refractive indices of 2.62 and 2.53 at temperatures of 400 °C and 600 °C, respectively, while dielectric constants were 6.87 and 6.42, respectively. The final stage of the study was the determination of the width of energy gaps of the produced bismuth oxide nanostructures, which were found to be 3.19 and 2.97 eV, respectively. The presented results of morphology and optical properties of the obtained one-dimensional Bi_2_O_3_ semiconductor nanostructures indicate a potential possibility to apply this type of materials for the production of a new generation of dye-sensitized photovoltaic cells (DSSCs).

## Introduction

In recent years, oxide nanomaterials in the form of nanotubes^1–3^, nanoparticles^4–6^, nanowires^7–10^, nanofibres^11–14^ and thin films^15–17^ have been of great interest for research and application, characterised by unprecedented properties compared to commonly used, conventional materials. The semiconductor nanostructures obtained from simple oxides, such as TiO_2_, ZnO and Bi_2_O_3_, due to the easy method of obtaining them as well as their wide application possibilities, are of special interest both in the field of scientific research and the increasingly widespread use in the field of industry^18–22^.

Nanostructures are used not only as an additive in the form of a nanofiller for composites, thus improving the properties of traditional engineering materials. Nowadays, the development of the electrical and optoelectronic devices industry is perceived through the production, testing and use of one-dimensional semiconductor bismuth oxide structures^23,24^. The huge application potential of Bi_2_O_3_ nanostructures is also associated with their very good photocatalytic properties, which are a response to the constantly growing demand for innovative and highly effective technologies for environmental protection, opening up a new possibility to use bismuth oxide nanostructures to degrade toxins in polluted air and water^25^.

Bismuth(III) oxide is a semiconductor with excellent physical properties, especially optical and electrical properties, such as the wide value of the energy gap contributing to the very good photocatalytic properties of Bi_2_O_3_ (α-Bi_2_O_3_ 2,91 eV, β-Bi_2_O_3_ 2,51 eV, γ- Bi_2_O_3_ 2,8 eV)^26–28^, high refractive index and dielectric constant, very good photoconductivity and luminescence properties^29^. Despite its advantages, bismuth(III) oxide is a material rarely produced in the synthesis of one-dimensional oxide nanostructures (Supplementary Informations 1, 2, 3, 4, 5).

Probably the first publication on the generation of one-dimensional bismuth oxide (Bi_2_O_3_) nanostructures by electrospinning was a paper published in 2008 by a Chinese team led by Changhua Wang^30^. A mixture of polyacrylonitrile (PAN) in *N*,*N*-dimethyl formamide (DMF) with 10 wt% polymer concentration relative to the solvent was used as the base for the spinning solution. Then, to obtain PAN/Bi(NO_3_)_3_ hybrid nanofibres, 1 g of bismuth(*III*) nitrate (Bi(NO_3_)_3_)was added to 20 ml of PAN/DMF base solution. The spinning solution prepared in this way was stirred for 4 h at room temperature. The next step was to subject the obtained PAN/DMF/Bi(NO_3_)_3_ solution to electrospinning with fixed parameters, which included a distance between the nozzle and collector of 10 cm and a voltage between the electrodes equal to 10 kV. However, the authors of this paper did not provide information on the rate at which the spinning solution was fed to the nozzle. The PAN/Bi(NO_3_)_3_ composite fibres obtained did not exhibit structural defects and their diameters ranged from 200 to 300 nm. To degrade the organic part from the PAN/Bi(NO_3_)_3_ fibres and obtain one-dimensional Bi_2_O_3_ nanostructures, the spun nanofibres were calcined at 500 °C for 10 h with a heating rate of 2 °C/min. Bi_2_O_3_ nanofibres with diameters of 70–100 nm, β-type crystal phase, and band gap of 2.6 eV were obtained in this way. Moreover, the one-dimensional bismuth oxide nanostructures obtained by Changhua Wang's team exhibited photocatalytic properties in the ultraviolet range through degradation of rhodamine B.

In 2009, the same team led by Changhua Wang presented a publication^31^ on the effect of calcination temperature on the structure and properties of Bi_2_O_3_ nanofibres produced by electrospinning. The methodology for producing the spinning solutions as well as the electrospinning parameters were the same as those presented in^30^, but in this case, the authors reported the flow rate of the spinning solution through the nozzle, which was 1 mL/h. The calcination process parameters, such as heating rate and annealing time for PAN/Bi(NO_3_)_3_ hybrid nanofibres obtained were the same, except that apart from calcination temperature of 500 °C, additional temperatures of 550 °C and 600 °C were also applied. Analysis of the morphology, structure, and properties of the obtained ceramic Bi_2_O_3_ nanofibres showed that annealing of PAN/Bi(NO_3_)_3_ hybrid nanofibres at 500 °C allowed the achievement of one-dimensional Bi_2_O_3_ nanostructures with β-type crystalline phase and diameters of 70–100 nm. The application of higher temperatures during the calcination process resulted in an increase in the diameters of Bi_2_O_3_ nanofibres to 120–150 nm for 550 °C and 150–200 °C for 600 °C, respectively. Moreover, the application of higher temperatures during the calcination process resulted in the appearance of trace amounts of α-phase in the crystal structure of Bi_2_O_3_ nanostructures and an increase in the width of band gaps amounting to 2.55, 2.88, and 2.97 eV for _Bi2_O_3_one-dimensional nanostructures calcined at 500, 550 and 600 °C, respectively. Analysis of the photocatalytic properties of the obtained bismuth oxide nanofibres showed that the efficiency of rhodamine B decomposition decreases with an increase in the temperature applied during the calcination process.

In 2015, a scientific team from Hong Kong lead by Carina Chun Pei presented a method of producing TiO_2_/ZnO/Bi_2_O_3_ hybrid nanofibres with enhanced photocatalytic properties^32^. Two solutions were mixed as the base for the spinning solutions. The first one was ethanol with 4 wt% PVP, to which they added a 3 wt% solution of TIIP and isometric acetic acid. The mixture thus prepared was ultrasonicated for 30 min, and then zinc acetate dehydrate and bismuth (III) nitrate pentahydrate were added to it in an amount not specified by the authors and ultrasonication continued for another 6 h. The prepared spinning solution was subjected to electrospinning, but the authors did not provide details of the process parameters. The obtained fibrous mat was subjected to calcination at 650 °C. Crystals corresponding to TiO_2_ (anatase), ZnO, and Bi_2_O_3_ were found on the structure of the obtained ceramic nanofibres. Concerning optical properties, the analysis showed that as Bi participation in the nanofibres increased, with the subsequent values being 0.1%, 0.2%, 0.3%, and 0.4, the band gaps were 2.74 eV, 2.51 eV 2.81 eV, and 2.85 eV, respectively. The photocatalytic efficiency of TiO_2_/ZnO/Bi_2_O_3_ nanofibres produced proved a significantly higher rate of conversion of nitrogen monoxide (NO) and degradation of o-xylene compared to TiO_2_/ZnO and TiO2/Bi_2_O_3_ composite nanofibres.

In 2018, a Chinese research team led by Kai Wang presented a publication on SnO_2_/Bi_2_O_3_/BiOI multi-heterojunction nanofibres generated using the solution electrospinning method^33^. One-dimensional SnO_2_/Bi_2_O_3_/BiOI structures were based on SnO2/Bi_2_O_3_ composite nanofibres obtained by the sol–gel and electrospinning methods. A polymer solution consisting of 1.5 g PVP, 5 ml DMF, and 5 ml ethanol was the base for the spinning solution. The mixture thus prepared was mechanically stirred for 12 h and then an unspecified amount of SnCl4—5H2O and BiOI was added, and the whole was stirred for another 0.5 h. The final spinning solution was subjected to electrospinning with the following parameters: a distance between the nozzle and the collector of 15 cm and a voltage between the electrodes equal to 15 kV. The resulting fibrous composite mat was calcined for 3 h at 600 °C. To produce one-dimensional SnO_2_/Bi_2_O_3_/BiOI composite nanostructures, the obtained SnO_2_/Bi_2_O_3_ ceramic nanofibres were added to 50 mL of KI solution and then stirred continuously for 0.5 h. As a result of I- ion exchanging, the Bi_2_O_3_ present in the ceramic fibres was transformed into BiOI nanosheets, which were deposited on the surface of the base SnO_2_/Bi_2_O_3_ nanofibres. In their morphology, the obtained one-dimensional nanostructures exhibited an average diameter value of SnO2/Bi_2_O_3_ nanofibres of about 250 nm, while the average diameter value of BiOI nanosheets located on the surface of these fibres was about 350 nm. Crystalline phases, corresponding to SnO2, Bi_2_O_3_, and BiOI, respectively, were found in the analysis of the structures using TEM and XRD. Photocatalytic efficiency analyses showed that SnO2/Bi2O3/BiOI multi-heterojunctions exhibited more than six times higher Cr(VI) ion and MO dye degradation efficiency compared to pure SnO_2_ nanofibres and SnO2/Bi_2_O_3_ composite nanofibres.

In 2019, a South Korean research team lead by Gyu-Dam Lim presented a method of producing Bi_2_O_3_ nanofibres with α-β phase heterojunctions using the electrospinning method^34^. To prepare the spinning solution, 10 g N*,N*-dimethylformamide, 8 g polyvinylpyrrolidone, and 1.75 g bismuth nitrate pentahydrate were used. The final spinning solution was subjected to electrospinning with the following parameters: a distance between the nozzle and the collector of 10 cm, a flow rate of the spinning solution of 0.4 ml/h, and a voltage between the electrodes of 20 kV. A drum collector rotating at 400 rpm was the electrode to which the fibres were applied. The resulting nanofibres were dried at 60 °C and then calcined at 325, 350, and 375 °C respectively for 10 h with a heating rate of 3 °C/min in each case. Surface topography analysis of the obtained PVP/Bi(NO_3_)_3_ fibrous mats showed that the electrospun fibres were free of structural defects, moreover, they were characterized by constant diameter values along their entire length with the average diameter value being was 64 nm. Calcination processes of the PVP/Bi(NO_3_)_3_ hybrid nanofibres yielded one-dimensional Bi_2_O_3_ nanostructures with average diameter values of 45 nm, 64 nm, and 75 nm, respectively, for the applied temperatures of 325, 350, and 375 °C. XRD examination of the structure of the obtained bismuth oxide nanofibres showed that the lowest applied temperature during the calcination process yielded pure tetragonal β-Bi_2_O_3_ structures. Analysis of diffractograms obtained for Bi_2_O_3_ nanofibres at 350 and 375 °C, respectively, showed the emergence of peaks corresponding to monoclinic α-phase Bi_2_O_3_ and the intensity of peaks for α-phase increased with increasing calcination temperature and decreased for β-phase. Examination of the optical properties of the generated Bi_2_O_3_ nanostructures, carried out using a UV–Vis spectrophotometer, showed that irrespective of the temperature used during the calcination process, all nanofibres had an absorption edge in the visible light range with length ranging from 500 to 550 nm. Moreover, the authors showed that Bi_2_O_3_ nanofibres consisting of α-β, phase heterojunctions calcined at 350 °C presented the best photocatalytic properties during RhB decomposition under visible light.

In 2020, S. Veeralingam and S. Badhulika from the Department of Electrical Engineering, Indian Institute of Technology Hyderabad described how to functionalize the surface of electrospun β-Bi_2_O_3_ nanofibres to serve as a base for fabricating flexible field-effect transistor-biosensor (BioFET) for rapid, label-free detection of serotonin in biological fluids^35^. Two separate mixtures were prepared to obtain the final spinning solution. For the first, 2.39 M bismuth nitrate was added to 15 ml dimethyl formaldehyde. The second consisted of a mixture of 1.2 M polyvinyl pyrolidine with 5 ml glacial acetic acid. The resulting mixtures were then stirred for 12 h until the precursors were completely dissolved. The nanofibre electrospinning process took place under constant parameters, which included a voltage between the nozzle and collector of 18 kV, a distance between the electrodes of 12 cm, and a solution flow rate of 0.5 ml/h. To produce β-Bi_2_O_3_ ceramic nanofibres, the obtained PVP/Bi(NO_3_)_3_ hybrid nanofibres were calcined at 450 °C for two hours. Analysis of surface morphology of the fabricated one-dimensional bismuth oxide nanostructures, performed using a scanning electron microscope, showed that the nanofibres exhibited constant diameter values along their entire length, with an average diameter value of 25–30 nm. Further in their paper, the focus was placed on functionalizing the surface of the obtained nanofibres, using Al nanoparticles and fabricating a field-effect transistor-based biosensor with them. The performed tests showed that the obtained sensors performed very well in detecting serotonin, which was attributed to the excellent electrochemical properties of the electrospun β-Bi_2_O_3_ nanofibres functionalized with Al.

In the same year, a Chinese research team led by Fan Yang reported a method of preparing CuBi_2_O_4_/Bi_2_O_3_ composite nanostructures using the solution electrospinning method^36^. 1.2 g polyvinylpyrrolidone, 15 ml dimethylformamide, 5 ml acetic acid, bismuth nitrate, and copper nitrate were used and stirred for 12 h to prepare the spinning solution. Depending on the type of sample, the following ratio of copper to bismuth precursors was used—Cu(NO_3_)_2_:Bi(NO_3_)_3_ = 0:1, 1:2, 1:4, and 1:16. They were prepared in the same way, keeping the quantity of Bi(NO_3_)_3_·5H_2_O and adjusting the quantity of Cu(NO_3_)_2_·6H_2_O. The solutions thus prepared were placed in the device pump and the electrospinning was performed with the following parameters: distance and voltage between the electrodes of 12 cm and 18 kV. The feeding rate of the spinning solution to the device nozzle was 0.24 ml/h. The resulting PVP/Bi(NO_3_)_3_/Cu(NO_3_)_2_ hybrid nanofibres were calcined at 650 °C for 2 h with a heating rate of 1 °C/min. Morphology analysis of the obtained one-dimensional Bi_2_O_3_ and Bi_2_O_3_/CuBi_2_O_4_ nanostructures showed that their diameters ranged from 250 to 350 nm. Moreover, with the increase in the number of Cu^2+^ ions in the nanofibre structure, the nanofibre surface developed more. XRD examination of this structure showed the presence of diffraction peaks corresponding to the α-Bi_2_O_3_ phase for pure Bi_2_O_3_ nanostructures, while an increasing presence of Cu^2+^ ions was accompanied by an increase in the intensity of diffraction peaks corresponding to CuBi_2_O_4_. The analysis of optical and electrochemical properties showed that for the Bi_2_O_3_/CuBi_2_O_4_ composite nanofibres both the electromagnetic radiation absorption and photocatalytic performance are better compared to the pure α-Bi_2_O_3_ and CuBi_2_O_4_ structures.

Among the techniques that enable the production of amorphous one-dimensional bismuth(III) oxide nanostructures, there are no reports of the use of electrospinning from solutions that would allow the production of nanofibres with nanometric diameters. In this work, the author presented a method for obtaining amorphous bismuth oxide nanowires using a combination of sol–gel and spinning in an electrostatic field. By using the thus obtained hybrid nanofibres with a polymer matrix doped with the Bi_2_O_3_ precursor, then submitting the thus obtained fibrous mats to the calcination process, which resulted in the lack of crystallisation, one-dimensional bismuth oxide nanostructures with an amorphous structure were obtained. In order to fully understand the physical properties of the amorphous Bi_2_O_3_ nanostructures, their optical properties have been tested, reflecting the full electrical nature of the material, which clearly shows that materials of this type are characterised by wide application possibilities.

## Material and methods

### Preparation of PAN/Bi_2_O_3_ thin fibrous mats and Bi_2_O_3_ nanowires

To prepare the Bi_2_O_3_ nanowires, PAN/DMF/Bi(NO_3_)_3_·5H_2_O spinning solutions were used, which were produced using the following products: polyacrylonitrile (PAN, purity of 99%, Mw = 150 000 g/mole), N,N-dimethylformamide (DMF, purity of 99.8%) and bismuth(III) nitrate pentahydrate (Bi(NO_3_)_3_ · 5H_2_O, purity of 98%). All products used were provided by the company Sigma-Aldrich.

**Figure 1:**
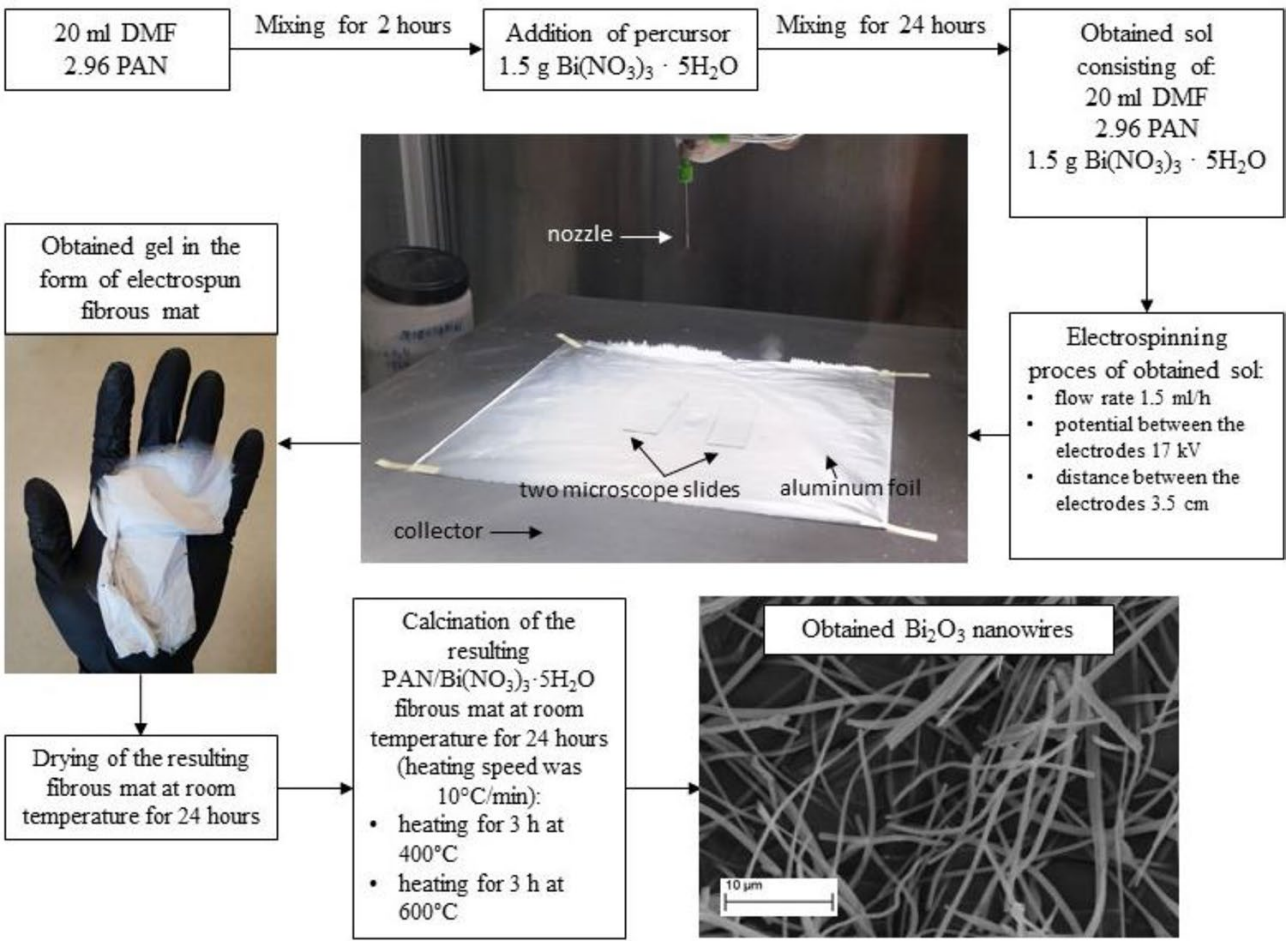
Experimental procedure scheme for fabrication of Bi_2_O_3_ nanostructures using sol–gel process, electrospinning and calcination methods.

The Fig. 1 shows a detailed diagram of the production of bismuth oxide nanowires using the sol–gel and electrospinning methods (Fig. 1). To spinning solution preparation, a 13% polyacrylonitrile solution was prepared in dimethylformamide to obtain bismuth oxide nanowires. For complete dissolution of the polymer, the obtained mixture was stirred for 2 h at room temperature with a magnetic stirrer. Then, 1.5 g of bismuth(III) nitrate pentahydrate was added to the thus prepared 13% PAN/DMF solution and it was stirred for another 24 h. Immediately after mixing, the solution was placed in the device's pump and subjected to the electrospinning process. Hybrid PAN/Bi_2_O_3_ nanofibres were obtained using the FLOW—Nanotechnology Solutions Electrospinner 2.2.0–500. During the electrospinning process, constant parameters were used, which included: distance and voltage between the nozzle and the collector of 3.5 cm and 17 kV, respectively, and a flow rate of the spinning solution through the nozzle of 1.5 ml/h. Immediately after production, the fibrous mats were allowed to dry at room temperature, and then they were subjected to the calcination process in a HT-2100-G-Vac-Graphit-Special high-temperature vacuum furnace oven at temperatures of 400 °C and 600 °C in the vacuum for a time of 3 h (heating speed was 10 °C/min).

### Characterization

In order to analyse the morphology and structure of the produced one-dimensional Bi_2_O_3_ structures, a high-resolution transmission electron microscope TITAN 80–300 from FEI was used. The transmission and scanning mode using light and dark field (BF, DF), HAADF detector and an energy filter were used during the analysis. In addition, to analyse the surface topography of the obtained PAN/Bi(NO_3_)_3_ · 5H_2_O nanofibers and Bi_2_O_3_ nanowires, the Zeiss Supra 35 scanning electron microscope (Zeiss) was used. Based on the taken surface topography images, the diameters of fifty randomly selected PAN/Bi(NO_3_)_3_ · 5H_2_O nanofibers and Bi_2_O_3_ nanowires were measured using the Digital Micrograph program (GATAN) and their average value was determined.The methodology of analyzing the morphology and structure of one-dimensional nanostructures using electron microscopy was thoroughly presented by the authors in^13,14^.

To analyse the chemical composition and oscillatory transitions of atoms vibrating between the oscillatory levels in the molecules of the produced bismuth oxide 1D nanostructures and to determine the functional groups existing therein, energy dispersive spectrometry (EDX) (Trident XM4, EDAX ) and Fourier-Transform Infrared spectroscopy (FTIR) (Nicolet™ iS™ 50 FTIR Spectrometer, Thermo Fisher Scientific) were used. The methodology of EDX and FTIR analysis was thoroughly presented by the authors in^14,44^.

To test the optical and electrical properties of the obtained bismuth(III) oxide nanowires, they were applied to glass microscope slides (Thermo Scientific, Menzel Gläser, refractive index of about 1.5) and then they were subjected to UV–Vis spectroscopic analysis. Measurements of the absorbance of the obtained materials as a function of the length of electromagnetic radiation incident on the sample were carried out using a Thermo-Scientific UV/VIS Evolution 220 spectrophotometer (Thermo Fisher Scientific). Then, Based on the received spectra of absorbance as a function of the wavelength using the method presented in^13,14^, the refractive index n, real n′ and imaginary k part of the refractive index as a function of the wavelength, complex dielectric permeability ε, and real and imaginary part εr and εi of the dielectric permeability as a function of the radiation wavelength of the produced ceramic bismuth(III) oxide nanowires, were used to determine the impact of the temperature used during the calcination process on the their physical properties. In addition, using the obtained Abs(λ) spectra, the energy band gap of the produced Bi_2_O_3_ nanowires was determined.

## Results and discussion

### Analysis of morphology and structure

#### TEM analysis

**Figure 2:**
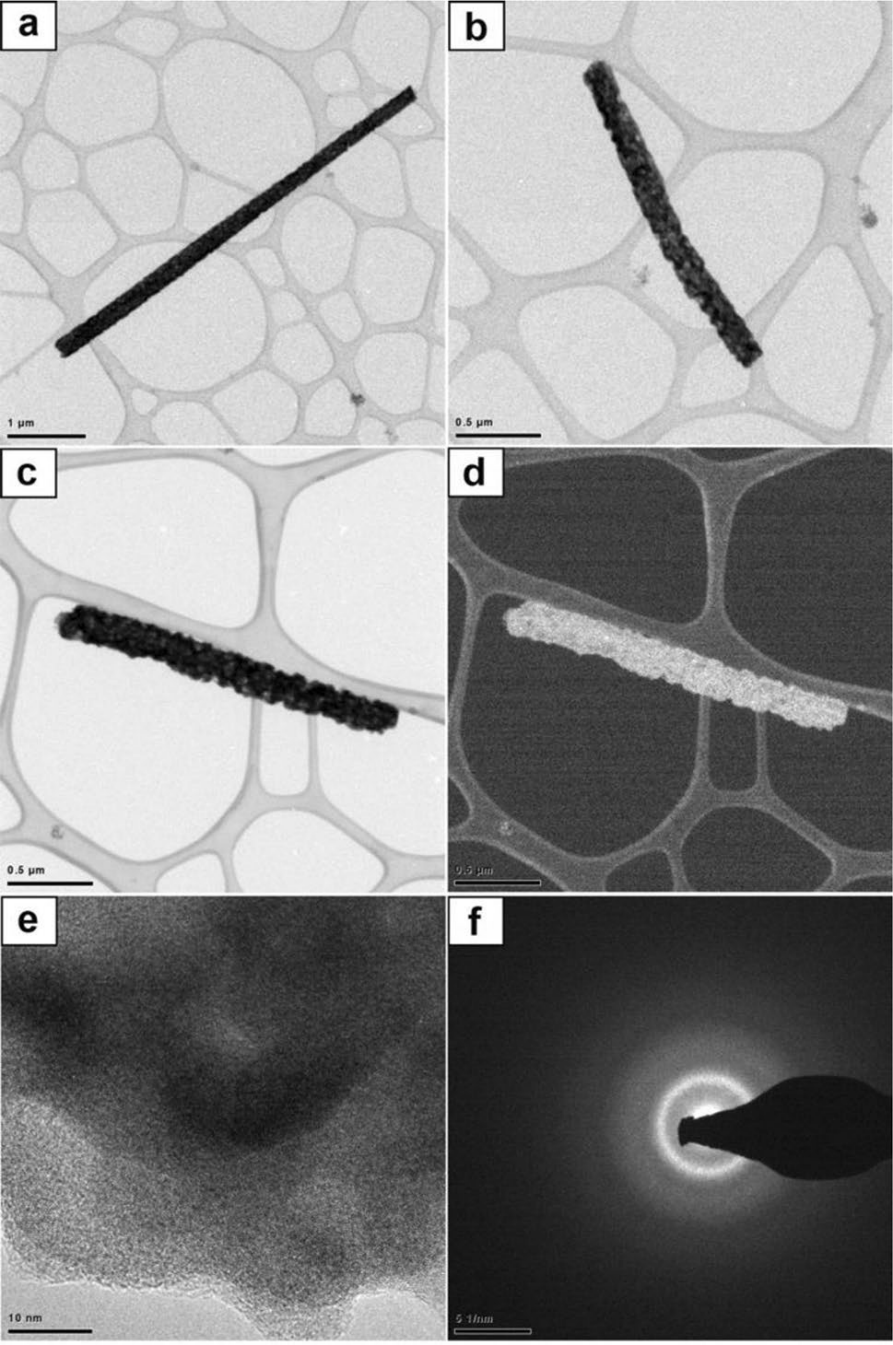
TEM images of the obtained one-dimensional bismuth oxide nanostructures produced by calcination of fibrous composite PAN/Bi_2_O_3_ mats at 600 °C made in transmission bronzing (**a**), (**b**), scanning-transmission mode, using bright (BF) and dark field (DF) (**c**), (**d**), HAADF detector (**e**) and using analytical microscopy in nano areas in the STEM mode (**f**).

The analysis of the structures of the obtained ceramic nanostructures based on electron diffraction images, carried out with the aid of a TEM microscope, confirmed that it is possible to produce amorphous one-dimensional bismuth oxide nanostructures through a combination of the sol–gel and electrospinning methods from PAN/Bi(NO_3_)_3_ · 5H_2_O/DMF solutions and calcination of the obtained PAN/Bi_2_O_3_ fibrous mats at high temperatures. The use of two different calcination temperatures of 400 and 600 °C in order to degrade organic parts from the electrospun fibrous hybrid mats contributed to obtaining amorphous bismuth(III) oxide nanowires regardless of the temperature used (Fig. 2).

The morphology analysis of obtained 1D Bi_2_O_3_ nanomaterials produced by calcination of hybrid PAN/ Bi(NO_3_)_3_ · 5H2O nanofibres at high temperatures, showed that the dimensions of the presented bismuth(III) oxide structures were 260 nm for the diameter and 5.5 μm long (Fig. 2a), 125 nm for the diameter and 2 μm long (Fig. 2b) and 200 nm for the diameter and 2.125 μm long (Fig. 2c, d). Considering the thickness to length ratio of obtained one-dimensional nanostructures, allows to classify them as ceramic nanowires.

Studies of the structure of produced Bi_2_O_3_ nanowires carried out with, as well as with the use of the HAADF detector, and results of diffraction studies obtained with the use of analytical microscopy in nano areas in the STEM mode, allowed to determine the phase composition and amorphous structure of the studied nanostructures (Fig. 2c–f). The TEM images (Fig. 2c, d) showed a uniform contrast in the whole area of the studied bismuth oxide nanostructures, which proves the absence of crystallites in their structure. Moreover, further analysis of the obtained TEM images showed that the presented Bi_2_O_3_ structures consisted of many fine, amorphous grains of various sizes and shapes (Fig. 2b–d). The electron diffraction spectrum obtained for a single Bi_2_O_3_ nanowire produced at 600 °C, in the form of fuzzy rings formed as a result of electron beam scattering, indicates their amorphous character (Fig. 2f). The TEM examinations of the morphology and structures of the obtained one-dimensional bismuth(III) oxide ceramic nanostructures permitted to define the influence of the applied calcination temperatures on the structure, morphology, optical and electrical properties of the produced one-dimensional bismuth oxide nanostructures.

#### FTIR analysis

**Figure 3:**
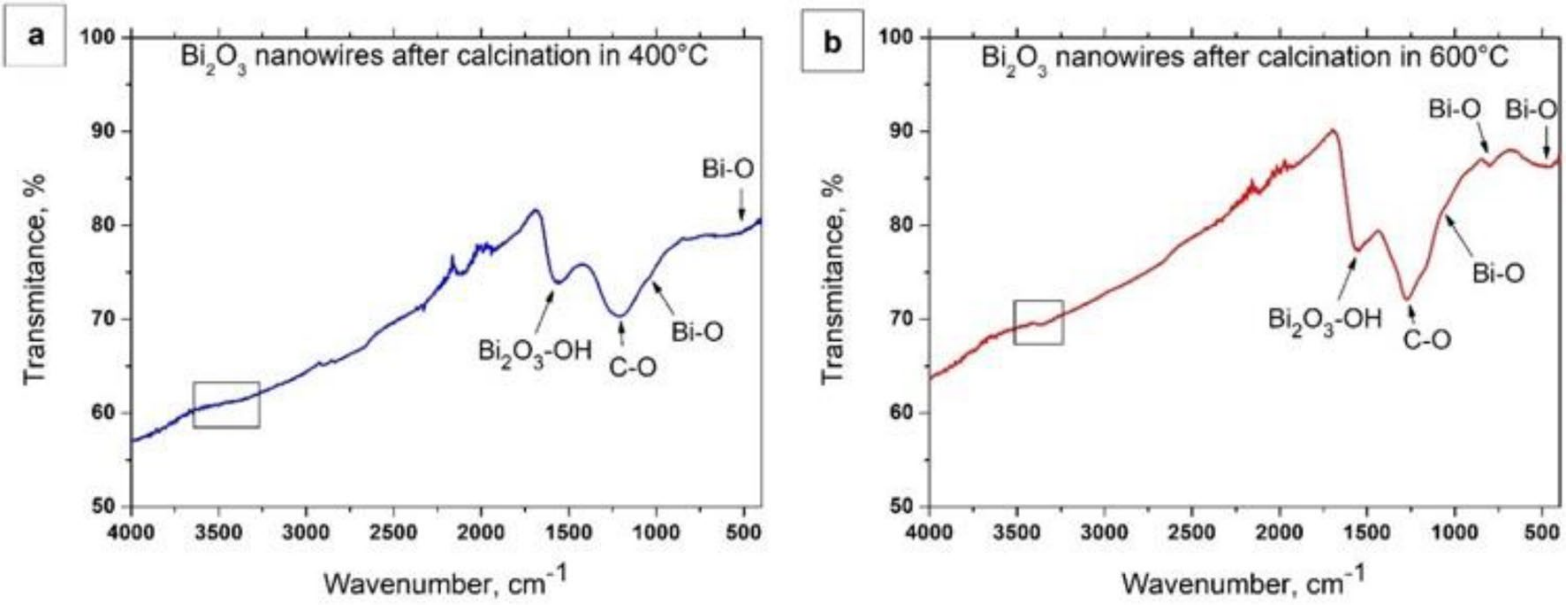
FTIR spectra recorded for the obtained one-dimensional bismuth oxide nanostructures produced by calcination of fibrous composite PAN/Bi_2_O_3_ mats at 400 °C (**a**) and 600 °C (**b**).

In order to analyse the oscillatory transitions of atoms vibrating between the oscillatory levels in the molecules of the produced bismuth oxide nanowires and to determine the functional groups existing therein, the transmittance FTIR spectra were analysed as a function of the wavenumber in the range from 400 to 4000 cm^-1^. A wide band visible in the wavenumber range equalling to 3500 cm^-1^ (marked with rectangles—Fig. 3), visible both in the case of Bi_2_O_3_ one-dimensional nanostructures obtained by calcination of hybrid PAN/Bi (NO_3_)_3_ · 5H_2_O nanofibres at 400 °C (Fig. 3a) and 600 °C (Fig. 3b), corresponds to the stretching vibrations of hydrogen-bonded surface water molecules and hydroxyl groups. The transmission peak visible to both samples in the wavenumber range is about 1600 cm^-1^ and probably corresponds to residual hydroxyl groups of the Bi_2_O_3_-OH type.

A wide band in the range from 1300 to 1200 cm^-1^, whose maximum peak is 1250 cm^-1^, is caused by the occurrence of vibrations in C-O bonds, which are the result of the use of an organic solvent for the production of a spinning solution, i.e. *N, N*-Dimethylformamide^14^. The intensity of the peak recorded for the wavenumber of 1025 cm-1 decreases with the increase in the temperature used during the calcination process of the hybrid nanofibres. This fact may be related to differences in sizes (diameters) of the obtained bismuth oxide nanostructures depending on the temperature at which fibrous composite PAN/Bi(NO_3_)_3_ · 5H_2_O mats were heated, which was indicated by the authors of the work^37^. Transmission peaks recorded for wavenumbers in the range from 800 to 400 cm-1 are caused by the occurrence of vibrations in Bi–O type bonds, corresponding to the structure of Bi_2_O_3_^31,37^, which unambiguously confirms obtaining one—dimensional bismuth(III) oxide nanostructures without visible impurities.

#### SEM and EDS analysis

**Figure 4:**
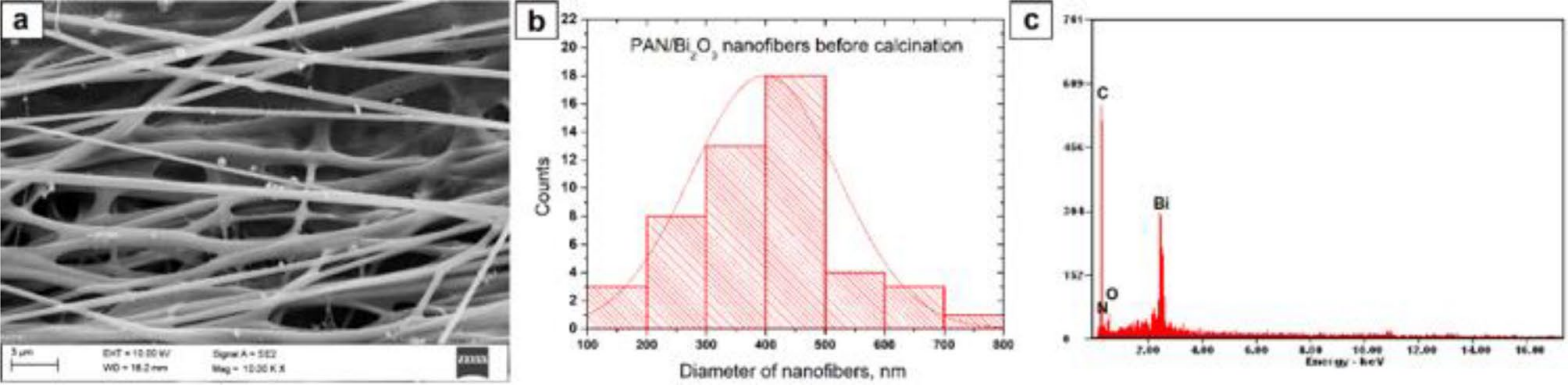
SEM image of the topography on the surface of the formed fibrous PAN/Bi_2_O_3_ composite mats (**a**), histogram presenting the distribution of the diameter of randomly selected nanofibers obtained from PAN/Bi(NO_3_)_3_ · 5H_2_O/DMF solution (**b**), EDS spectra (the strong signal of the C peak, except the signal from the polymer, derive from the substrate on which nanofibers were deposited) from the entire area shown in the SEM images (**c**).

**Figure 5:**
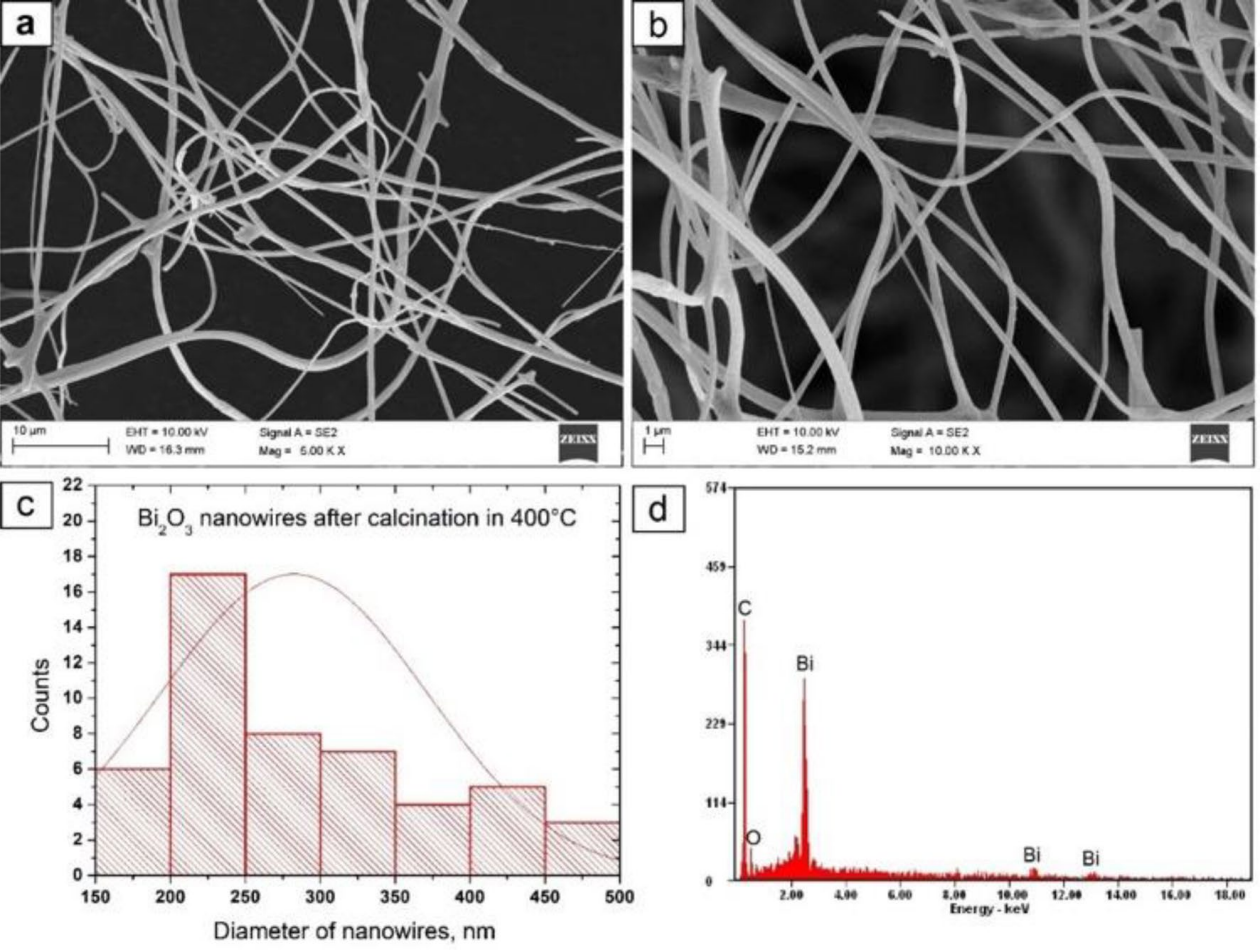
SEM image of the topography on the surface of obtained nanowires after calcination the PAN/Bi_2_O_3_ fibrous composite mats in 400 °C, magnification 5 000 x (**a**) and 10 000 x (**b**), histograms presenting the distribution of the diameter of randomly selected nanowires (**c**), EDS spectra (peak of C derive from the substrate on which nanowires were deposited) from the entire area shown in the SEM images (**d**).

**Figure 6:**
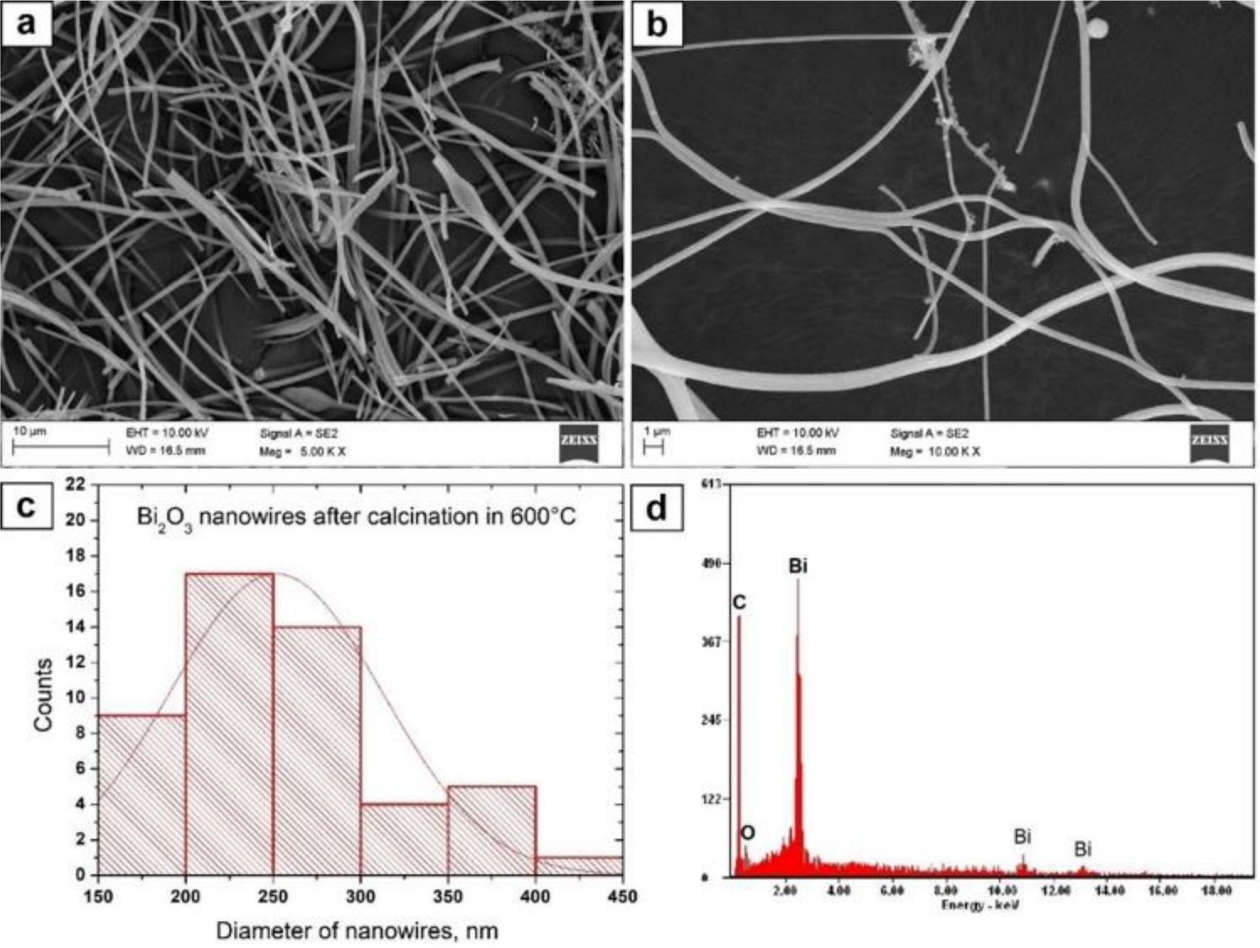
SEM image of the topography on the surface of obtained nanowires after calcination the PAN/Bi_2_O_3_ fibrous composite mats in 600 °C, magnification 5 000 x (**a**) and 10 000 x (**b**), histograms presenting the distribution of the diameter of randomly selected nanowires (**c**), EDS spectra (peak of C derive from the substrate on which nanowires were deposited) from the entire area shown in the SEM images (**d**).

To determine the morphology and chemical composition of the produced hybrid PAN/Bi(NO_3_)_3_ · 5H_2_O nanofibres as well as the obtained Bi_2_O_3_ nanowires, the scanning electron microscope with EDX detector were used (Figs. 4, 5, 6). The analysis of the hybrid PAN/Bi_2_O_3_ nanofibres (Fig. 4), obtained from the PAN/Bi(NO_3_)_3_ · 5H_2_O/DMF spinning solution with a 13% concentration by weight of the polymer, showed that the obtained fibres constituting the starting material for producing one-dimensional bismuth(III) oxide nanostructures were free from structural defects in the form of spherical and fusiform beads and were characterised by constant diameters along their entire lengths (Fig. 4a). The analysisof single hybrid PAN nanofibres containing Bi (NO_3_)_3_ · 5H_2_O precursor particles in their volume, showed that the most numerous group of nanofibers, constituting 36%, were non-fibers with diameters of 400–500 nm, while all the measured diameters were within the 100–800 nm range. The average value of nanofiber diameter for this sample was 390 nm. (Fig. 4b).

The next stage of the research consisting in subjecting the obtained fibrous mat consisting of electrospunned PAN/Bi_2_O_3_ hybrid nanofibres to the process of high temperature calcination for three hours allowed to obtain clean one-dimensional bismuth oxide nanostructures that were free from structural defects, regardless of the temperature used during heating, which was confirmed by the analysis of the EDS spectra obtained for these nanomaterials (Figs. 5d, 6d). Regardless of the temperature used when heating the samples, the obtained Bi_2_O_3_ nanowires were characterised by a lack of structural defects (Figs. 5a, b, 6a, b), and the 50-fold measurement of the diameters of the obtained nanowires showed that the diameters measured were near to the nanoscale range. As a result of heating the fibrous PAN mat containing Bi_2_O_3_ precursor particles at 400 °C, bismuth oxide nanowires were obtained, whose diameters ranged from 150 to 500 nm, while subjecting the same fibrous mat to the calcination process at a higher temperature of 600 °C allowed to obtain Bi_2_O_3_ nanowires, whose diameters were in a narrower range of 150–450 nm (Figs. 5c, 6c). In addition, along with the increase in the temperature of the degradation of the polymer constituting a matrix of calcined hybrid nanofibres, the average value of the diameters of the analysed nanowires decreased and was 277 nm for 400 °C nm and 247 nm for 600 °C, successively.

This fact may indicate the possibility of controlling the morphology of the produced bismuth oxide nanowires by changing the temperature used during the calcination process. The possibility to control the average values of the diameters of the produced bismuth oxide nanowires may be crucial for future application possibilities of this type of materials, with particular emphasis on dye sensitized photovoltaic cells and photocatalysis reaction, where the largest specific surface of the applied nanostructures is important, thus having an effect on the increase in the contact of the active surface with reagents.

### Analysis of optical properties

**Figure 7:**
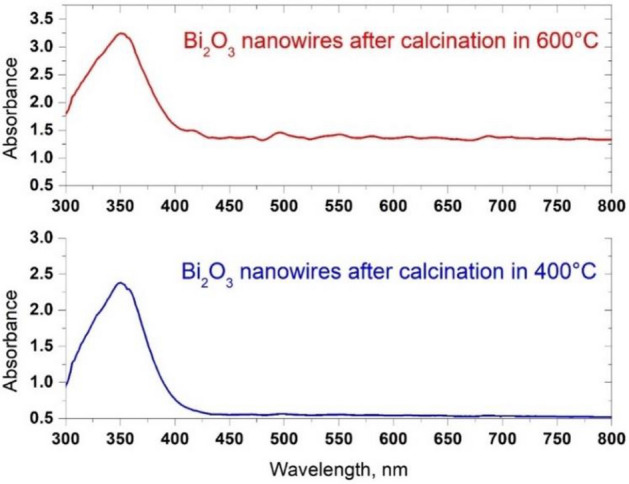
UV–Vis spectra recorded for the obtained one-dimensional bismuth oxide nanostructures produced by calcination of fibrous composite PAN/Bi_2_O_3_ mats at 400 °C (**a**) and 600 °C (**b**).

In order to analyse the effect of the temperature used during the calcination process of PAN/Bi_2_O_3_ hybrid nanofibres on the optical properties of the thus produced one-dimensional bismuth oxide nanostructures, the absorbance spectra as a function of the wavelength were obtained for all nanowires obtained using a UV–Vis spectrometer (Fig. 7). Spectral characteristics recorded for the produced one-dimensional Bi_2_O_3_ nanowires, obtained using the sol–gel method and electrospinning from a PAN/Bi (NO_3_)_3_ · 5H_2_O/DMF solution, had sharp edges of radiation absorption in the near ultraviolet range, with the sharp edges of absorption falling for waves with a length of around 400 nm regardless of the temperature used during the calcination process. A typical absorption edge recorded for bismuth oxide is for waves with a length of 450 nm^37–39^. The presented results of UV–Vis analyses of Bi_2_O_3_ nanowires produced as the effect of the combination of the sol–gel method and electrospinning from the solution coincide with the results presented in work^31^, and the shift of the absorption edge of electromagnetic radiation in the visible wavelength range towards the near ultraviolet may probably be caused by the micrometric thickness of the layer of bismuth oxide nanowires analysed during the study^40^.

With the increase in the temperature used during the calcination process of hybrid PAN/Bi_2_O_3_ nanofibres, an increase was observed in absorption maxima corresponding to the excitonic absorption that occurs for waves 350 nm long, from the value of 2.38 for one-dimensional bismuth oxide nanostructures obtained at 400 °C, to the value of 3.25 for Bi_2_O_3_ nanowires obtained during the calcination process carried out at 600 °C. By analysing the absorption spectra of electromagnetic radiation recorded for titanium oxide and zinc oxide nanowires^41,42^, it can be concluded that the observed increase in the absorption of electromagnetic radiation in the near ultraviolet area recorded for one-dimensional bismuth oxide nanostructures testifies to potentially more effective application possibilities of this type of nanostructures both in terms of the construction of photovoltaic cells and the materials used in photocatalytic reactions.

Based on the received spectra of absorbance as a function of the wavelength using the method presented in^13,14^, the following optical and electrical properties were determined: the refractive index n, real n′ and imaginary k part of the refractive index as a function of the wavelength, complex dielectric permeability ε, and real and imaginary part εr and εi of the dielectric permeability as a function of the radiation wavelength of the produced ceramic bismuth(III) oxide nanowires, using the final equations in the following form:

1 $$ n = \left\{ {2n_{p} \frac{{10^{ - A\left( \lambda \right)}_{max} - 10^{ - A\left( \lambda \right)}_{min} }}{{10^{ - A\left( \lambda \right)}_{max} { }10^{ - A\left( \lambda \right)}_{min} }} + \frac{{n_{p}^{2} + 1}}{2} + \left[ {\left( {2n_{p} \frac{{10^{ - A\left( \lambda \right)}_{max} - 10^{ - A\left( \lambda \right)}_{min} }}{{10^{ - A\left( \lambda \right)}_{max} { }10^{ - A\left( \lambda \right)}_{min} }} + \frac{{n_{p}^{2} + 1}}{2}} \right)^{2} - n_{p}^{2} } \right]^{{{\raise0.7ex\hbox{$1$} \!\mathord{\left/ {\vphantom {1 2}}\right.\kern-\nulldelimiterspace} \!\lower0.7ex\hbox{$2$}}}} } \right\}^{{{\raise0.7ex\hbox{$1$} \!\mathord{\left/ {\vphantom {1 2}}\right.\kern-\nulldelimiterspace} \!\lower0.7ex\hbox{$2$}}}} , $$

where $n_{p}$—the refractive index of the substrate used (refractive index of glass microscope slide – 1.5),

2 $$ n^{^{\prime}} \left( \lambda \right) = \left\{ {\frac{4 R\left( \lambda \right) }{{\left[ {R\left( \lambda \right) - 1} \right]^{2} }} - \left[ {\frac{1}{4\pi }\lambda \ln \frac{1}{T\left( \lambda \right)}} \right]^{2} } \right\}^{{{\raise0.7ex\hbox{$1$} \!\mathord{\left/ {\vphantom {1 2}}\right.\kern-\nulldelimiterspace} \!\lower0.7ex\hbox{$2$}}}} - \frac{R\left( \lambda \right) + 1}{{R\left( \lambda \right) - 1}}, $$

3 $$ k\left( \lambda \right) = \frac{1}{4\pi }\lambda { }ln\frac{1}{{10^{ - A\left( \lambda \right)} }}, $$

4 $$ \varepsilon = 2n_{p} \frac{{10^{ - A\left( \lambda \right)}_{max} - 10^{ - A\left( \lambda \right)}_{min} }}{{10^{ - A\left( \lambda \right)}_{max} { }10^{ - A\left( \lambda \right)}_{min} }} + \frac{{n_{p}^{2} + 1}}{2} + \left[ {\left( {2n_{p} \frac{{10^{ - A\left( \lambda \right)}_{max} - 10^{ - A\left( \lambda \right)}_{min} }}{{10^{ - A\left( \lambda \right)}_{max} { }10^{ - A\left( \lambda \right)}_{min} }} + \frac{{n_{p}^{2} + 1}}{2}} \right) - n_{p}^{2} } \right]^{{{\raise0.7ex\hbox{$1$} \!\mathord{\left/ {\vphantom {1 2}}\right.\kern-\nulldelimiterspace} \!\lower0.7ex\hbox{$2$}}}} $$

5 $$ \varepsilon_{r} \left( \lambda \right) = \left\{ {\left\{ {\frac{4 R\left( \lambda \right) }{{\left[ {R\left( \lambda \right) - 1} \right]^{2} }} - \left[ {\frac{1}{4\pi }\lambda \ln \frac{1}{T\left( \lambda \right)}} \right]^{2} } \right\}^{{{\raise0.7ex\hbox{$1$} \!\mathord{\left/ {\vphantom {1 2}}\right.\kern-\nulldelimiterspace} \!\lower0.7ex\hbox{$2$}}}} - \frac{R\left( \lambda \right) + 1}{{R\left( \lambda \right) - 1}}} \right\}^{2} - \left[ {\frac{1}{4\pi }\lambda \ln \frac{1}{T\left( \lambda \right)}} \right]^{2} $$

6 $$ \varepsilon_{i} \left( \lambda \right) = 2 \left\{ {\left\{ {\frac{4 R\left( \lambda \right) }{{\left[ {R\left( \lambda \right) - 1} \right]^{2} }} - \left[ {\frac{1}{4\pi }\lambda \ln \frac{1}{T\left( \lambda \right)}} \right]^{2} } \right\}^{{{\raise0.7ex\hbox{$1$} \!\mathord{\left/ {\vphantom {1 2}}\right.\kern-\nulldelimiterspace} \!\lower0.7ex\hbox{$2$}}}} - \frac{R\left( \lambda \right) + 1}{{R\left( \lambda \right) - 1}}} \right\} \left[ {\frac{1}{4\pi }\lambda \ln \frac{1}{T\left( \lambda \right)}} \right] $$

**Figure 8:**
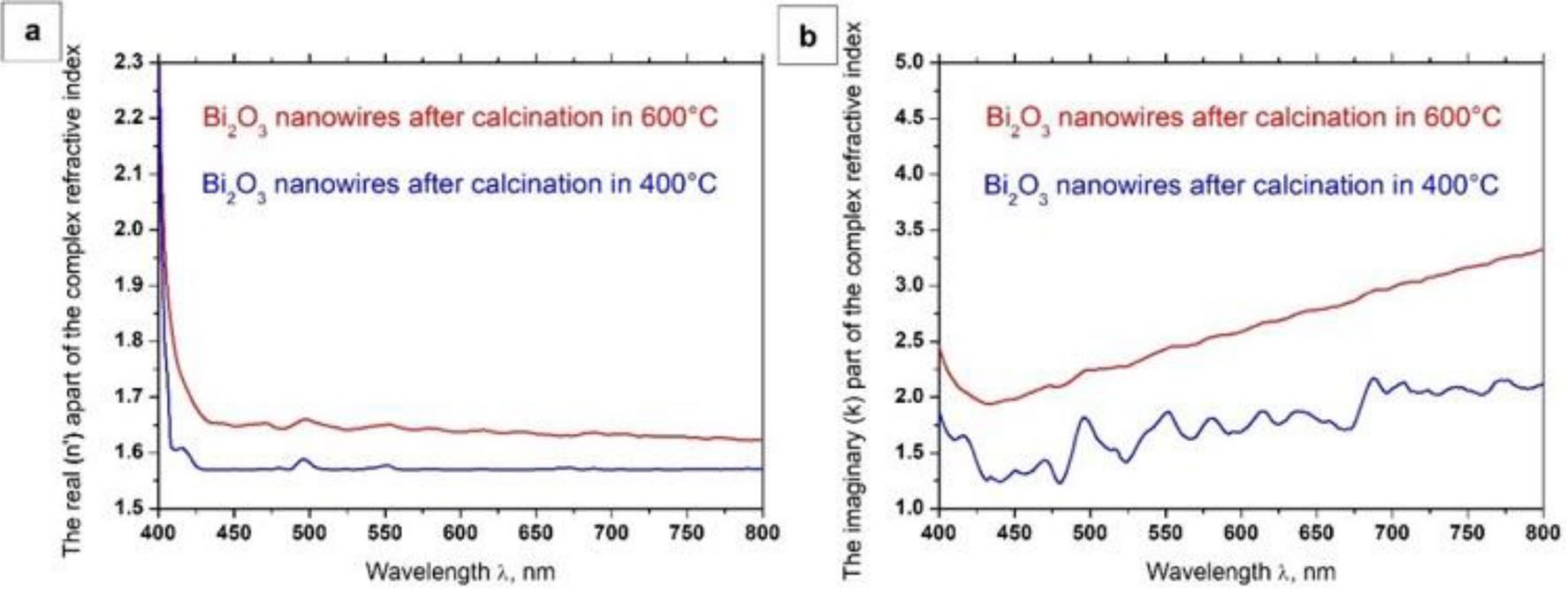
The dependences of the real *n*′(*λ*) (**a**) and imaginary *k*(*λ*) parts (**b**) of the refractive index determined for the one-dimensional bismuth oxide nanostructures produced by calcination of fibrous composite PAN/Bi_2_O_3_ mats at 400 °C and 600 °C.

**Figure 9:**
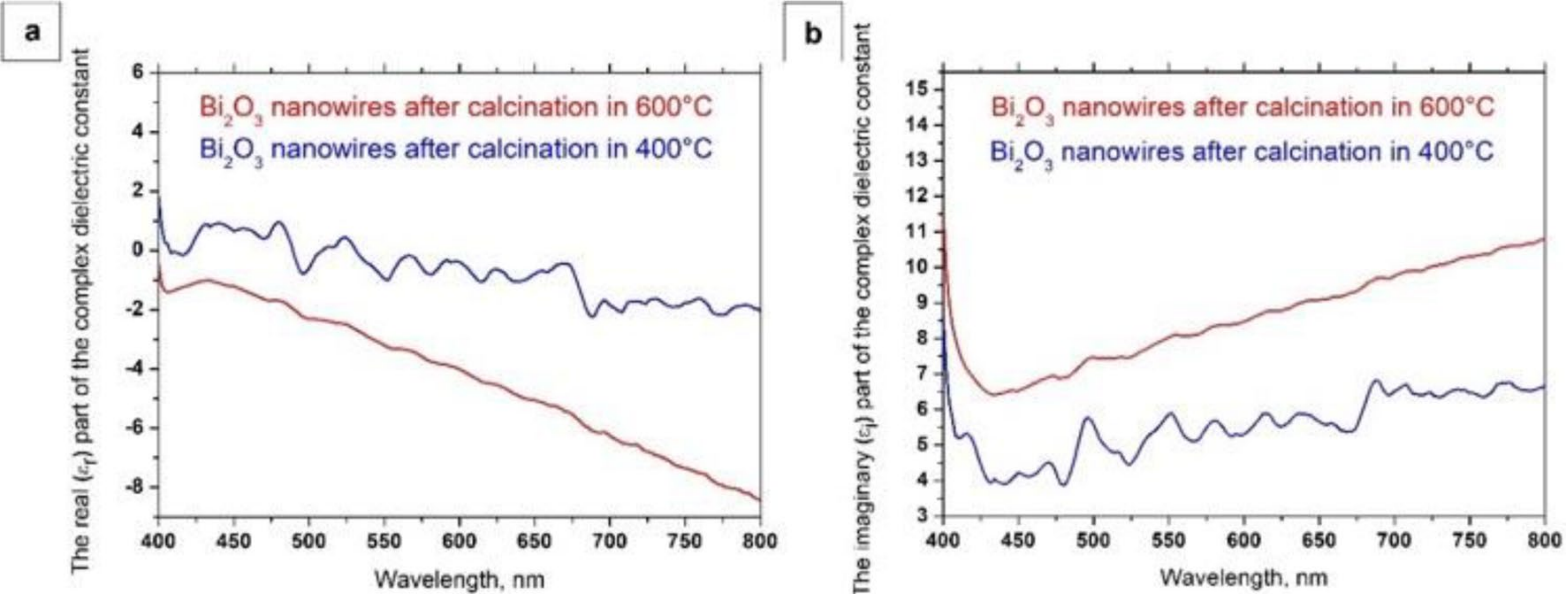
The dependences of the real *ε*_*r*_(*λ*), (**a**) and imaginary *ε*_*i*_(*λ*) parts (**b**) of the dielectric constant for the one-dimensional bismuth oxide nanostructures produced by calcination of fibrous composite PAN/Bi_2_O_3_ mats at 400 °C and 600 °C.

Based on the above equations and UV–Vis spectra obtained as a function of the wavelength of electromagnetic radiation incident on the sample, recorded for the produced semiconductor one-dimensional Bi_2_O_3_ nanostructures, dependences of the $n^{^{\prime}} \left( \lambda \right)$, $k\left( \lambda \right)$ and $\varepsilon_{r} \left( \lambda \right),$ $\varepsilon_{i} \left( \lambda \right)$, were determined (Figs. 8 and 9), and complex values of the refractive index (Eq. 1), dielectric permittivity (Eq. 4), and energy band gap for the formed bismuth(III) oxide nanostructures were determined with the equation

7 $$ \left[ {h\nu \ln \left( {\frac{1}{{10^{ - ABS} }}} \right)} \right]^{2} = B\left( {h\nu - E_{g} } \right), $$

where B is a result of relationship between the probability of electron transitions in electronic structure and thickness of the analyzed layer, and the obtained results are presented in Table 1.

**Table 1:** Determined values of the complex refractive index, dielectric permittivity and energy gap width values obtained for the produced nanowires.

Parameter	Bi_2_O_3_ nanowires	
	Calcination temperature, °C	
	400	600
*n*	2.62	2.53
ε	6.87	6.42
*E*_*g*_, eV	3.19	2.97

Complex refractive index analyses performed on the produced one-dimensional bismuth oxide nanostructures showed that calcining fine fibrous mat consisting of hybrid nanofibres with a polyacrylonitrile matrix containing Bi(NO_3_)_3_ · 5H_2_O precursor particles at 400 °C for 3 h contributed to obtaining Bi_2_O_3_ nanowires, which were characterised by a refractive index of 2.62. The use of a higher temperature of 600 °C when heating PAN/Bi_2_O_3_ nanofibres contributed to the formation of bismuth oxide nanowires characterised by a lower value of the complex refractive index, which, in this case, was 2.53. The obtained values of the complex refractive index determined for the produced Bi_2_O_3_ nanowires are similar to the results presented in the work^43^. In addition, the decrease in the determined complex refractive index values of the obtained one-dimensional bismuth oxide nanostructures accompanying the temperature increase during the calcination process from 400 to 600 °C is probably related to the morphology of the tested ceramic nanostructures. The analyses carried out testify to the influence of the diameter of the tested nanowires on their optical properties, which can be confirmed by the results presented in the work^40^. This fact indicates the possibility of producing semiconductor one-dimensional nanostructures of bismuth oxide, characterised by the same structure and chemical composition, as well as selected optical properties matched by manipulating the diameters of individual nanowires produced using a combination of the sol–gel method and electrospinning from a solution. The analysis of complex dielectric permittivity showed a decrease in the dielectric constant from the value of 6.87 in the case of Bi_2_O_3_ nanowires produced by calcination of hybrid PAN/Bi(NO_3_)_3_ · 5H_2_O nanofibres at 400 °C, to the value of 6.42 for nanowires obtained at 600 °C.

**Figure 10:**
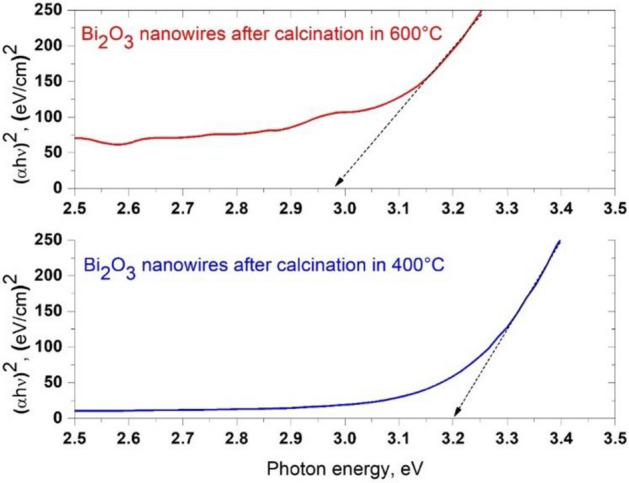
Formulas *(αhν)*^2^ as a function of the quantum radiation energy obtained for one-dimensional bismuth oxide nanostructures produced by calcination of fibrous composite PAN/Bi_2_O_3_ mats at 400 °C and 600 °C.

The analysis of the energy band gap based on recorded Abs(λ) spectra showed that the Bi_2_O_3_ nanofibres obtained in the calcination process carried out at 400 °C were characterised by an energy barrier value between the conduction band and the valence band of 3.19 eV (Fig. 10). The use of a higher temperature during the process of heating hybrid nanofibres with a polyacrylonitrile matrix containing Bi(NO_3_)_3_ · 5H_2_O precursor particles resulted in obtaining one-dimensional Bi_2_O_3_ nanostructures characterised by smaller values of the energy band gap, in this case, 2.97 eV. The obtained values of energy gaps coincide with the results presented in the work^40^ and show simple electron transitions between the valence band and the conduction band in the analysed Bi_2_O_3_ nanowires. The opposite tendency for the decrease in the values of determined energy gaps depending on the temperature used during the calcination process, presented in the work^31^, can be explained by a different composition of the spinning solution used during the electrospinning process of PAN/Bi_2_O_3_ hybrid nanofibres and, what follows, differences in the structure and morphology of the obtained one-dimensional bismuth oxide nanostructures.

## Conclusions

The work presents the technique of the production of one-dimensional Bi_2_O_3_ nanostructures using a combination of sol–gel and electrospinning techniques from a 13% polymer solution based on polyacrylonitrile (PAN) and dimethylformamide (DMF) containing bismuth(III) nitrate pentahydrate (Bi(NO_3_)_3_·5H_2_O). In the first stage of the research, with the use of the prepared spinning solution PAN/DMF/Bi(NO_3_)_3_·5H_2_O and the electrospinning process carried out with constant process parameters (distance and voltage between the nozzle and the collector of 3.5 and 17 kV, and a flow rate of the spinning solution of 1.5 ml/h), thin fibrous mats consisting of PAN/Bi_2_O_3_ hybrid nanofibres were obtained. The nanofibres produced in this way were subjected to a calcination process using two different temperatures of 400 °C and 600 °C to degrade the polymer and obtain ceramic one-dimensional Bi_2_O_3_ nanostructures. The analysis of the structures, chemical composition and morphology of the produced nanowires showed that as a result of calcinations, amorphous bismuth oxide nanowires with average diameter values of 277 nm, respectively, were obtained at 400 °C, as well as 247 nm when heated at 600 °C. The literature review on the fabrication of one-dimensional Bi_2_O_3_ nanostructures using a combination of sol–gel process, electrospinning and calcination methods—as presented in the Introduction part—shows that no detailed analysis of optical and electrical properties of such nanostructures (refractive index *n*, real *n*′ and imaginary *k* part of the refractive index as a function of the wavelength, complex dielectric permeability *ε*, and real and imaginary part *ε*_*r*_ and *ε*_*i*_ of the dielectric permeability as a function of the radiation energy) has been performed to date. Therefore, a detailed analysis of optical constants of one-dimensional Bi_2_O_3_ nanostructures fabricated using a combination of sol–gel process, electrospinning and calcination methods has been presented in this paper for the first time. Testing the optical and electrical properties of bismuth (III) oxide nanostructures conducted on the basis of recorded Abs (λ) spectra showed that, with an increase in the temperature used during the calcination process, the complex refractive index decreased (from 2.62 to 2.53), the dielectric constant decreased (from 6.87 to 6.42) and the value of the energy band gap decreased from 3.19 eV to 2.97 eV. The results of the one-dimensional semiconductor Bi2O3 nanostructures presented in this work indicate the potential for controlling both the morphology as well as the optical and electrical properties of the obtained nanomaterials, thus increasing the scope of their future application possibilities. The obtained results of research into the optical and electrical properties of the manufactured Bi_2_O_3_ nanostructures indicate the potential possibility of using this type of materials for the construction of a new generation of dye-sensitized photovoltaic cells (DSSCs).

## Supplementary Information

Supplementary Information 1.

Supplementary Information 2.

Supplementary Information 3.

Supplementary Information 4.

Supplementary Information 5.
