# Supplementary material for: Synthesis of 1D Bi2O3 nanostructures from hybrid electrospun fibrous mats and their morphology, structure, optical and electrical properties
Source: Sci Rep. 2022 Mar 8;12:4046. doi: 10.1038/s41598-022-07830-z (PMC8904472; doi:10.1038/s41598-022-07830-z)
Supplement: Supplementary file 1 — Supplementary Information 1. [file 41598_2022_7830_MOESM1_ESM.pdf]

3.991926e+002	8.058855e+001
4.011211e+002	8.061019e+001
4.030496e+002	8.075254e+001
4.049780e+002	8.073753e+001
4.069065e+002	8.064567e+001
4.088350e+002	8.031722e+001
4.107634e+002	8.021442e+001
4.126919e+002	8.028954e+001
4.146204e+002	8.054771e+001
4.165488e+002	8.062214e+001
4.184773e+002	8.044926e+001
4.204058e+002	8.057813e+001
4.223342e+002	8.038983e+001
4.242627e+002	8.015974e+001
4.261912e+002	8.025513e+001
4.281196e+002	8.028528e+001
4.300481e+002	8.031054e+001
4.319766e+002	8.012152e+001
4.339050e+002	8.004468e+001
4.358335e+002	8.002873e+001
4.377620e+002	8.002640e+001
4.396904e+002	7.999825e+001
4.416189e+002	8.005089e+001
4.435474e+002	8.001201e+001
4.454758e+002	8.004659e+001
4.474043e+002	7.992018e+001
4.493328e+002	7.992432e+001
4.512612e+002	7.991537e+001
4.531897e+002	7.982247e+001
4.551182e+002	7.987387e+001
4.570466e+002	7.984201e+001
4.589751e+002	7.971322e+001
4.609036e+002	7.964326e+001
4.628320e+002	7.968362e+001
4.647605e+002	7.970596e+001
4.666890e+002	7.972951e+001
4.686174e+002	7.983225e+001
4.705459e+002	7.977295e+001
4.724744e+002	7.973124e+001
4.744028e+002	7.958144e+001
4.763313e+002	7.961427e+001
4.782598e+002	7.966822e+001
4.801882e+002	7.959466e+001
4.821167e+002	7.951372e+001
4.840452e+002	7.947964e+001
4.859736e+002	7.947266e+001
4.879021e+002	7.954102e+001
4.898306e+002	7.958054e+001
4.917590e+002	7.962257e+001
4.936875e+002	7.951163e+001

4.956160e+002	7.938419e+001
4.975444e+002	7.933472e+001
4.994729e+002	7.943971e+001
5.014014e+002	7.936934e+001
5.033298e+002	7.934726e+001
5.052583e+002	7.936386e+001
5.071868e+002	7.932304e+001
5.091152e+002	7.930190e+001
5.110437e+002	7.929318e+001
5.129722e+002	7.919612e+001
5.149006e+002	7.916704e+001
5.168291e+002	7.918308e+001
5.187576e+002	7.918443e+001
5.206860e+002	7.913793e+001
5.226145e+002	7.917099e+001
5.245430e+002	7.919851e+001
5.264714e+002	7.916680e+001
5.283999e+002	7.907119e+001
5.303284e+002	7.911116e+001
5.322568e+002	7.921346e+001
5.341853e+002	7.924454e+001
5.361138e+002	7.916631e+001
5.380422e+002	7.915341e+001
5.399707e+002	7.913436e+001
5.418992e+002	7.911691e+001
5.438276e+002	7.912565e+001
5.457561e+002	7.916931e+001
5.476846e+002	7.916821e+001
5.496130e+002	7.911459e+001
5.515415e+002	7.907304e+001
5.534700e+002	7.903690e+001
5.553984e+002	7.898424e+001
5.573269e+002	7.900774e+001
5.592554e+002	7.902570e+001
5.611838e+002	7.904488e+001
5.631123e+002	7.908615e+001
5.650408e+002	7.911535e+001
5.669692e+002	7.905285e+001
5.688977e+002	7.907204e+001
5.708262e+002	7.906300e+001
5.727546e+002	7.901972e+001
5.746831e+002	7.898517e+001
5.766116e+002	7.898482e+001
5.785400e+002	7.899078e+001
5.804685e+002	7.899322e+001
5.823970e+002	7.897324e+001
5.843254e+002	7.894070e+001
5.862539e+002	7.892612e+001
5.881824e+002	7.887211e+001
5.901108e+002	7.885043e+001

5.920393e+002	7.893584e+001
5.939678e+002	7.894884e+001
5.958962e+002	7.893449e+001
5.978247e+002	7.890361e+001
5.997532e+002	7.886185e+001
6.016816e+002	7.884250e+001
6.036101e+002	7.886977e+001
6.055386e+002	7.890197e+001
6.074670e+002	7.891701e+001
6.093955e+002	7.890385e+001
6.113240e+002	7.892397e+001
6.132524e+002	7.890260e+001
6.151809e+002	7.887174e+001
6.171094e+002	7.887505e+001
6.190378e+002	7.889367e+001
6.209663e+002	7.887714e+001
6.228948e+002	7.889362e+001
6.248232e+002	7.890849e+001
6.267517e+002	7.892978e+001
6.286802e+002	7.892110e+001
6.306086e+002	7.890295e+001
6.325371e+002	7.887825e+001
6.344656e+002	7.889397e+001
6.363940e+002	7.889745e+001
6.383225e+002	7.891386e+001
6.402510e+002	7.890011e+001
6.421794e+002	7.891106e+001
6.441079e+002	7.891136e+001
6.460364e+002	7.887659e+001
6.479648e+002	7.888184e+001
6.498933e+002	7.890931e+001
6.518218e+002	7.890572e+001
6.537502e+002	7.890634e+001
6.556787e+002	7.890067e+001
6.576072e+002	7.896431e+001
6.595356e+002	7.897472e+001
6.614641e+002	7.893232e+001
6.633926e+002	7.891965e+001
6.653210e+002	7.888931e+001
6.672495e+002	7.876881e+001
6.691780e+002	7.891142e+001
6.711064e+002	7.896871e+001
6.730349e+002	7.896679e+001
6.749634e+002	7.895317e+001
6.768918e+002	7.893215e+001
6.788203e+002	7.890452e+001
6.807488e+002	7.890797e+001
6.826772e+002	7.893742e+001
6.846057e+002	7.899295e+001
6.865342e+002	7.900657e+001

6.884626e+002	7.897905e+001
6.903911e+002	7.893209e+001
6.923196e+002	7.893401e+001
6.942480e+002	7.891315e+001
6.961765e+002	7.891653e+001
6.981050e+002	7.894324e+001
7.000334e+002	7.897511e+001
7.019619e+002	7.897275e+001
7.038904e+002	7.896334e+001
7.058188e+002	7.894928e+001
7.077473e+002	7.893549e+001
7.096758e+002	7.893753e+001
7.116042e+002	7.894257e+001
7.135327e+002	7.895120e+001
7.154612e+002	7.896703e+001
7.173896e+002	7.896537e+001
7.193181e+002	7.897316e+001
7.212466e+002	7.896075e+001
7.231750e+002	7.895859e+001
7.251035e+002	7.894076e+001
7.270320e+002	7.892528e+001
7.289604e+002	7.889698e+001
7.308889e+002	7.889419e+001
7.328174e+002	7.888026e+001
7.347458e+002	7.888988e+001
7.366743e+002	7.889110e+001
7.386028e+002	7.889172e+001
7.405313e+002	7.885133e+001
7.424597e+002	7.883651e+001
7.443882e+002	7.883135e+001
7.463167e+002	7.882978e+001
7.482451e+002	7.882805e+001
7.501736e+002	7.885363e+001
7.521021e+002	7.883340e+001
7.540305e+002	7.883441e+001
7.559590e+002	7.883760e+001
7.578875e+002	7.879137e+001
7.598159e+002	7.877509e+001
7.617444e+002	7.878252e+001
7.636729e+002	7.877074e+001
7.656013e+002	7.876363e+001
7.675298e+002	7.873676e+001
7.694583e+002	7.870828e+001
7.713867e+002	7.867223e+001
7.733152e+002	7.863071e+001
7.752437e+002	7.861509e+001
7.771721e+002	7.864890e+001
7.791006e+002	7.866164e+001
7.810291e+002	7.865819e+001
7.829575e+002	7.865277e+001

7.848860e+002	7.865594e+001
7.868145e+002	7.865871e+001
7.887429e+002	7.863612e+001
7.906714e+002	7.860349e+001
7.925999e+002	7.860377e+001
7.945283e+002	7.860745e+001
7.964568e+002	7.861176e+001
7.983853e+002	7.857473e+001
8.003137e+002	7.855797e+001
8.022422e+002	7.855080e+001
8.041707e+002	7.853275e+001
8.060991e+002	7.851417e+001
8.080276e+002	7.855563e+001
8.099561e+002	7.858907e+001
8.118845e+002	7.857316e+001
8.138130e+002	7.856434e+001
8.157415e+002	7.855048e+001
8.176699e+002	7.852283e+001
8.195984e+002	7.851785e+001
8.215269e+002	7.850262e+001
8.234553e+002	7.851234e+001
8.253838e+002	7.851212e+001
8.273123e+002	7.848838e+001
8.292407e+002	7.847863e+001
8.311692e+002	7.847843e+001
8.330977e+002	7.844235e+001
8.350261e+002	7.844658e+001
8.369546e+002	7.848419e+001
8.388831e+002	7.853233e+001
8.408115e+002	7.857777e+001
8.427400e+002	7.862040e+001
8.446685e+002	7.861410e+001
8.465969e+002	7.860614e+001
8.485254e+002	7.858288e+001
8.504539e+002	7.857169e+001
8.523823e+002	7.855670e+001
8.543108e+002	7.855880e+001
8.562393e+002	7.855367e+001
8.581677e+002	7.851753e+001
8.600962e+002	7.848856e+001
8.620247e+002	7.842466e+001
8.639531e+002	7.835167e+001
8.658816e+002	7.834349e+001
8.678101e+002	7.832517e+001
8.697385e+002	7.827145e+001
8.716670e+002	7.824802e+001
8.735955e+002	7.819461e+001
8.755239e+002	7.813844e+001
8.774524e+002	7.809876e+001
8.793809e+002	7.806163e+001

8.813093e+002	7.807671e+001
8.832378e+002	7.808789e+001
8.851663e+002	7.804463e+001
8.870947e+002	7.801362e+001
8.890232e+002	7.799595e+001
8.909517e+002	7.795144e+001
8.928801e+002	7.790570e+001
8.948085e+002	7.786822e+001
8.967370e+002	7.785205e+001
8.986655e+002	7.786042e+001
9.005939e+002	7.780898e+001
9.025224e+002	7.776874e+001
9.044509e+002	7.774944e+001
9.063793e+002	7.770750e+001
9.083078e+002	7.767932e+001
9.102363e+002	7.766292e+001
9.121647e+002	7.766018e+001
9.140932e+002	7.763896e+001
9.160217e+002	7.760909e+001
9.179501e+002	7.759468e+001
9.198786e+002	7.757269e+001
9.218071e+002	7.753506e+001
9.237355e+002	7.749812e+001
9.256640e+002	7.745588e+001
9.275925e+002	7.744770e+001
9.295209e+002	7.741859e+001
9.314494e+002	7.738972e+001
9.333779e+002	7.737338e+001
9.353063e+002	7.738136e+001
9.372348e+002	7.735123e+001
9.391633e+002	7.731284e+001
9.410917e+002	7.727451e+001
9.430202e+002	7.721494e+001
9.449487e+002	7.717391e+001
9.468771e+002	7.715573e+001
9.488056e+002	7.713618e+001
9.507341e+002	7.709870e+001
9.526625e+002	7.702650e+001
9.545910e+002	7.696691e+001
9.565195e+002	7.692190e+001
9.584479e+002	7.687731e+001
9.603764e+002	7.683475e+001
9.623049e+002	7.678840e+001
9.642333e+002	7.672944e+001
9.661618e+002	7.668021e+001
9.680903e+002	7.663582e+001
9.700187e+002	7.658405e+001
9.719472e+002	7.654536e+001
9.738757e+002	7.649932e+001
9.758041e+002	7.642281e+001

9.777326e+002	7.635404e+001
9.796611e+002	7.630220e+001
9.815895e+002	7.623367e+001
9.835180e+002	7.617393e+001
9.854465e+002	7.610503e+001
9.873749e+002	7.602537e+001
9.893034e+002	7.597565e+001
9.912319e+002	7.592479e+001
9.931603e+002	7.588577e+001
9.950888e+002	7.585654e+001
9.970173e+002	7.582825e+001
9.989457e+002	7.575689e+001
1.000874e+003	7.569186e+001
1.002803e+003	7.563040e+001
1.004731e+003	7.556134e+001
1.006660e+003	7.549009e+001
1.008588e+003	7.544332e+001
1.010517e+003	7.539523e+001
1.012445e+003	7.533983e+001
1.014373e+003	7.526236e+001
1.016302e+003	7.518970e+001
1.018230e+003	7.511891e+001
1.020159e+003	7.506743e+001
1.022087e+003	7.501811e+001
1.024016e+003	7.494073e+001
1.025944e+003	7.486510e+001
1.027873e+003	7.481758e+001
1.029801e+003	7.475389e+001
1.031730e+003	7.465969e+001
1.033658e+003	7.455587e+001
1.035587e+003	7.448502e+001
1.037515e+003	7.443755e+001
1.039444e+003	7.439185e+001
1.041372e+003	7.435071e+001
1.043301e+003	7.432173e+001
1.045229e+003	7.430158e+001
1.047157e+003	7.426343e+001
1.049086e+003	7.422223e+001
1.051014e+003	7.421846e+001
1.052943e+003	7.420740e+001
1.054871e+003	7.417811e+001
1.056800e+003	7.414494e+001
1.058728e+003	7.410712e+001
1.060657e+003	7.404870e+001
1.062585e+003	7.399969e+001
1.064514e+003	7.394936e+001
1.066442e+003	7.389201e+001
1.068371e+003	7.385795e+001
1.070299e+003	7.381655e+001
1.072228e+003	7.374075e+001

1.074156e+003	7.368452e+001
1.076084e+003	7.364466e+001
1.078013e+003	7.356450e+001
1.079941e+003	7.349711e+001
1.081870e+003	7.348041e+001
1.083798e+003	7.344040e+001
1.085727e+003	7.337792e+001
1.087655e+003	7.332872e+001
1.089584e+003	7.325098e+001
1.091512e+003	7.317299e+001
1.093441e+003	7.311317e+001
1.095369e+003	7.304886e+001
1.097298e+003	7.298716e+001
1.099226e+003	7.292455e+001
1.101155e+003	7.284026e+001
1.103083e+003	7.277180e+001
1.105011e+003	7.270918e+001
1.106940e+003	7.262368e+001
1.108868e+003	7.255952e+001
1.110797e+003	7.248680e+001
1.112725e+003	7.240308e+001
1.114654e+003	7.236192e+001
1.116582e+003	7.231941e+001
1.118511e+003	7.223697e+001
1.120439e+003	7.216165e+001
1.122368e+003	7.209571e+001
1.124296e+003	7.202707e+001
1.126225e+003	7.194644e+001
1.128153e+003	7.191013e+001
1.130082e+003	7.185588e+001
1.132010e+003	7.178621e+001
1.133938e+003	7.173785e+001
1.135867e+003	7.167911e+001
1.137795e+003	7.161988e+001
1.139724e+003	7.156451e+001
1.141652e+003	7.148664e+001
1.143581e+003	7.143050e+001
1.145509e+003	7.139552e+001
1.147438e+003	7.135661e+001
1.149366e+003	7.130453e+001
1.151295e+003	7.123740e+001
1.153223e+003	7.114944e+001
1.155152e+003	7.108545e+001
1.157080e+003	7.104139e+001
1.159009e+003	7.099895e+001
1.160937e+003	7.095018e+001
1.162865e+003	7.088024e+001
1.164794e+003	7.082326e+001
1.166722e+003	7.078819e+001
1.168651e+003	7.074934e+001

1.170579e+003	7.069432e+001
1.172508e+003	7.063752e+001
1.174436e+003	7.059080e+001
1.176365e+003	7.057342e+001
1.178293e+003	7.055109e+001
1.180222e+003	7.051344e+001
1.182150e+003	7.049346e+001
1.184079e+003	7.047016e+001
1.186007e+003	7.042702e+001
1.187936e+003	7.039137e+001
1.189864e+003	7.037733e+001
1.191792e+003	7.038215e+001
1.193721e+003	7.037246e+001
1.195649e+003	7.035974e+001
1.197578e+003	7.035793e+001
1.199506e+003	7.033377e+001
1.201435e+003	7.030410e+001
1.203363e+003	7.029729e+001
1.205292e+003	7.029626e+001
1.207220e+003	7.028864e+001
1.209149e+003	7.028222e+001
1.211077e+003	7.028622e+001
1.213006e+003	7.029588e+001
1.214934e+003	7.029913e+001
1.216863e+003	7.029405e+001
1.218791e+003	7.030061e+001
1.220719e+003	7.031576e+001
1.222648e+003	7.033627e+001
1.224576e+003	7.035374e+001
1.226505e+003	7.036433e+001
1.228433e+003	7.038248e+001
1.230362e+003	7.041050e+001
1.232290e+003	7.042985e+001
1.234219e+003	7.044233e+001
1.236147e+003	7.045838e+001
1.238076e+003	7.049375e+001
1.240004e+003	7.053620e+001
1.241933e+003	7.056124e+001
1.243861e+003	7.057857e+001
1.245790e+003	7.059766e+001
1.247718e+003	7.060643e+001
1.249646e+003	7.063216e+001
1.251575e+003	7.067532e+001
1.253503e+003	7.071701e+001
1.255432e+003	7.075481e+001
1.257360e+003	7.078790e+001
1.259289e+003	7.081348e+001
1.261217e+003	7.085942e+001
1.263146e+003	7.090535e+001
1.265074e+003	7.092837e+001

1.267003e+003	7.096683e+001
1.268931e+003	7.100717e+001
1.270860e+003	7.104678e+001
1.272788e+003	7.110101e+001
1.274717e+003	7.115185e+001
1.276645e+003	7.120389e+001
1.278573e+003	7.125043e+001
1.280502e+003	7.131864e+001
1.282430e+003	7.138480e+001
1.284359e+003	7.144111e+001
1.286287e+003	7.149687e+001
1.288216e+003	7.155015e+001
1.290144e+003	7.162868e+001
1.292073e+003	7.172787e+001
1.294001e+003	7.181860e+001
1.295930e+003	7.188373e+001
1.297858e+003	7.196015e+001
1.299787e+003	7.205188e+001
1.301715e+003	7.215676e+001
1.303644e+003	7.225318e+001
1.305572e+003	7.233022e+001
1.307500e+003	7.242013e+001
1.309429e+003	7.252634e+001
1.311357e+003	7.260218e+001
1.313286e+003	7.269267e+001
1.315214e+003	7.280037e+001
1.317143e+003	7.288425e+001
1.319071e+003	7.296603e+001
1.321000e+003	7.308536e+001
1.322928e+003	7.320202e+001
1.324857e+003	7.330511e+001
1.326785e+003	7.340165e+001
1.328714e+003	7.350329e+001
1.330642e+003	7.362705e+001
1.332571e+003	7.373320e+001
1.334499e+003	7.381428e+001
1.336427e+003	7.387175e+001
1.338356e+003	7.391165e+001
1.340284e+003	7.405486e+001
1.342213e+003	7.419341e+001
1.344141e+003	7.427729e+001
1.346070e+003	7.436942e+001
1.347998e+003	7.445339e+001
1.349927e+003	7.452547e+001
1.351855e+003	7.460906e+001
1.353784e+003	7.468272e+001
1.355712e+003	7.475907e+001
1.357641e+003	7.482834e+001
1.359569e+003	7.488053e+001
1.361498e+003	7.491856e+001

1.363426e+003	7.502621e+001
1.365354e+003	7.510781e+001
1.367283e+003	7.514850e+001
1.369211e+003	7.520040e+001
1.371140e+003	7.521714e+001
1.373068e+003	7.521044e+001
1.374997e+003	7.528280e+001
1.376925e+003	7.535579e+001
1.378854e+003	7.540245e+001
1.380782e+003	7.546597e+001
1.382711e+003	7.551787e+001
1.384639e+003	7.551333e+001
1.386568e+003	7.550790e+001
1.388496e+003	7.558132e+001
1.390424e+003	7.562901e+001
1.392353e+003	7.565646e+001
1.394281e+003	7.565907e+001
1.396210e+003	7.565251e+001
1.398138e+003	7.565363e+001
1.400067e+003	7.571982e+001
1.401995e+003	7.573862e+001
1.403924e+003	7.573824e+001
1.405852e+003	7.579087e+001
1.407781e+003	7.581515e+001
1.409709e+003	7.580178e+001
1.411638e+003	7.582323e+001
1.413566e+003	7.584280e+001
1.415495e+003	7.579155e+001
1.417423e+003	7.575311e+001
1.419351e+003	7.583875e+001
1.421280e+003	7.585565e+001
1.423208e+003	7.581257e+001
1.425137e+003	7.585469e+001
1.427065e+003	7.584281e+001
1.428994e+003	7.581059e+001
1.430922e+003	7.581639e+001
1.432851e+003	7.578769e+001
1.434779e+003	7.575901e+001
1.436708e+003	7.580188e+001
1.438636e+003	7.581573e+001
1.440565e+003	7.577780e+001
1.442493e+003	7.576530e+001
1.444422e+003	7.574268e+001
1.446350e+003	7.568533e+001
1.448278e+003	7.566661e+001
1.450207e+003	7.565904e+001
1.452135e+003	7.562827e+001
1.454064e+003	7.561627e+001
1.455992e+003	7.560961e+001
1.457921e+003	7.559606e+001

1.459849e+003	7.560443e+001
1.461778e+003	7.558914e+001
1.463706e+003	7.557272e+001
1.465635e+003	7.556438e+001
1.467563e+003	7.552869e+001
1.469492e+003	7.547185e+001
1.471420e+003	7.536817e+001
1.473349e+003	7.533447e+001
1.475277e+003	7.534073e+001
1.477205e+003	7.536279e+001
1.479134e+003	7.531564e+001
1.481062e+003	7.523875e+001
1.482991e+003	7.522446e+001
1.484919e+003	7.520212e+001
1.486848e+003	7.510160e+001
1.488776e+003	7.498870e+001
1.490705e+003	7.504694e+001
1.492633e+003	7.504199e+001
1.494562e+003	7.499583e+001
1.496490e+003	7.493866e+001
1.498419e+003	7.488400e+001
1.500347e+003	7.484192e+001
1.502276e+003	7.479051e+001
1.504204e+003	7.481743e+001
1.506132e+003	7.459648e+001
1.508061e+003	7.449007e+001
1.509989e+003	7.451296e+001
1.511918e+003	7.451730e+001
1.513846e+003	7.446146e+001
1.515775e+003	7.436113e+001
1.517703e+003	7.438645e+001
1.519632e+003	7.439017e+001
1.521560e+003	7.429723e+001
1.523489e+003	7.430215e+001
1.525417e+003	7.426111e+001
1.527346e+003	7.421219e+001
1.529274e+003	7.421136e+001
1.531203e+003	7.417710e+001
1.533131e+003	7.413216e+001
1.535059e+003	7.409912e+001
1.536988e+003	7.402991e+001
1.538916e+003	7.399220e+001
1.540845e+003	7.398341e+001
1.542773e+003	7.396946e+001
1.544702e+003	7.392919e+001
1.546630e+003	7.397910e+001
1.548559e+003	7.391869e+001
1.550487e+003	7.390640e+001
1.552416e+003	7.395184e+001
1.554344e+003	7.393656e+001

1.556273e+003	7.388921e+001
1.558201e+003	7.379936e+001
1.560130e+003	7.409969e+001
1.562058e+003	7.394411e+001
1.563986e+003	7.385952e+001
1.565915e+003	7.387481e+001
1.567843e+003	7.385683e+001
1.569772e+003	7.390543e+001
1.571700e+003	7.393249e+001
1.573629e+003	7.392999e+001
1.575557e+003	7.402129e+001
1.577486e+003	7.411343e+001
1.579414e+003	7.408057e+001
1.581343e+003	7.411453e+001
1.583271e+003	7.414835e+001
1.585200e+003	7.421959e+001
1.587128e+003	7.429740e+001
1.589057e+003	7.437666e+001
1.590985e+003	7.442160e+001
1.592913e+003	7.449185e+001
1.594842e+003	7.457436e+001
1.596770e+003	7.470872e+001
1.598699e+003	7.481606e+001
1.600627e+003	7.490774e+001
1.602556e+003	7.500165e+001
1.604484e+003	7.516050e+001
1.606413e+003	7.525711e+001
1.608341e+003	7.540498e+001
1.610270e+003	7.559064e+001
1.612198e+003	7.578119e+001
1.614127e+003	7.591845e+001
1.616055e+003	7.616305e+001
1.617984e+003	7.641237e+001
1.619912e+003	7.649677e+001
1.621840e+003	7.665154e+001
1.623769e+003	7.700339e+001
1.625697e+003	7.718382e+001
1.627626e+003	7.741010e+001
1.629554e+003	7.769274e+001
1.631483e+003	7.790022e+001
1.633411e+003	7.809939e+001
1.635340e+003	7.831576e+001
1.637268e+003	7.864317e+001
1.639197e+003	7.889084e+001
1.641125e+003	7.911510e+001
1.643054e+003	7.934610e+001
1.644982e+003	7.952414e+001
1.646911e+003	7.971922e+001
1.648839e+003	7.990896e+001
1.650767e+003	8.014875e+001

1.652696e+003	8.036261e+001
1.654624e+003	8.080254e+001
1.656553e+003	8.074496e+001
1.658481e+003	8.084618e+001
1.660410e+003	8.088609e+001
1.662338e+003	8.100579e+001
1.664267e+003	8.115127e+001
1.666195e+003	8.119383e+001
1.668124e+003	8.118729e+001
1.670052e+003	8.128573e+001
1.671981e+003	8.137975e+001
1.673909e+003	8.148422e+001
1.675838e+003	8.154910e+001
1.677766e+003	8.153833e+001
1.679694e+003	8.147017e+001
1.681623e+003	8.147376e+001
1.683551e+003	8.147922e+001
1.685480e+003	8.169182e+001
1.687408e+003	8.151330e+001
1.689337e+003	8.152029e+001
1.691265e+003	8.156549e+001
1.693194e+003	8.163370e+001
1.695122e+003	8.168496e+001
1.697051e+003	8.148984e+001
1.698979e+003	8.143848e+001
1.700908e+003	8.160346e+001
1.702836e+003	8.141611e+001
1.704765e+003	8.132691e+001
1.706693e+003	8.130766e+001
1.708621e+003	8.135364e+001
1.710550e+003	8.129024e+001
1.712478e+003	8.122520e+001
1.714407e+003	8.111134e+001
1.716335e+003	8.120798e+001
1.718264e+003	8.123380e+001
1.720192e+003	8.116545e+001
1.722121e+003	8.104443e+001
1.724049e+003	8.098507e+001
1.725978e+003	8.093893e+001
1.727906e+003	8.090535e+001
1.729835e+003	8.082345e+001
1.731763e+003	8.087175e+001
1.733692e+003	8.089555e+001
1.735620e+003	8.082916e+001
1.737548e+003	8.065858e+001
1.739477e+003	8.058880e+001
1.741405e+003	8.048271e+001
1.743334e+003	8.040705e+001
1.745262e+003	8.033454e+001
1.747191e+003	8.026088e+001

1.749119e+003	8.027812e+001
1.751048e+003	8.032240e+001
1.752976e+003	8.024736e+001
1.754905e+003	8.017947e+001
1.756833e+003	8.007182e+001
1.758762e+003	8.005709e+001
1.760690e+003	7.999780e+001
1.762619e+003	7.995691e+001
1.764547e+003	7.991198e+001
1.766475e+003	7.985550e+001
1.768404e+003	7.974646e+001
1.770332e+003	7.968037e+001
1.772261e+003	7.961444e+001
1.774189e+003	7.958586e+001
1.776118e+003	7.949592e+001
1.778046e+003	7.947273e+001
1.779975e+003	7.940439e+001
1.781903e+003	7.938071e+001
1.783832e+003	7.929848e+001
1.785760e+003	7.931673e+001
1.787689e+003	7.933948e+001
1.789617e+003	7.931445e+001
1.791546e+003	7.927483e+001
1.793474e+003	7.929015e+001
1.795402e+003	7.917126e+001
1.797331e+003	7.914147e+001
1.799259e+003	7.913355e+001
1.801188e+003	7.916219e+001
1.803116e+003	7.913960e+001
1.805045e+003	7.909800e+001
1.806973e+003	7.904073e+001
1.808902e+003	7.901172e+001
1.810830e+003	7.896416e+001
1.812759e+003	7.899108e+001
1.814687e+003	7.900497e+001
1.816616e+003	7.895661e+001
1.818544e+003	7.891037e+001
1.820473e+003	7.887550e+001
1.822401e+003	7.884138e+001
1.824329e+003	7.885006e+001
1.826258e+003	7.875763e+001
1.828186e+003	7.868051e+001
1.830115e+003	7.867747e+001
1.832043e+003	7.867628e+001
1.833972e+003	7.858506e+001
1.835900e+003	7.857560e+001
1.837829e+003	7.861053e+001
1.839757e+003	7.862152e+001
1.841686e+003	7.852820e+001
1.843614e+003	7.849788e+001

1.845543e+003	7.840369e+001
1.847471e+003	7.836729e+001
1.849400e+003	7.834429e+001
1.851328e+003	7.838013e+001
1.853256e+003	7.833862e+001
1.855185e+003	7.825890e+001
1.857113e+003	7.814005e+001
1.859042e+003	7.817675e+001
1.860970e+003	7.818325e+001
1.862899e+003	7.816219e+001
1.864827e+003	7.813235e+001
1.866756e+003	7.807631e+001
1.868684e+003	7.811168e+001
1.870613e+003	7.820404e+001
1.872541e+003	7.801681e+001
1.874470e+003	7.790808e+001
1.876398e+003	7.798302e+001
1.878327e+003	7.796712e+001
1.880255e+003	7.792507e+001
1.882183e+003	7.794393e+001
1.884112e+003	7.801674e+001
1.886040e+003	7.805872e+001
1.887969e+003	7.789710e+001
1.889897e+003	7.778820e+001
1.891826e+003	7.777351e+001
1.893754e+003	7.768417e+001
1.895683e+003	7.760590e+001
1.897611e+003	7.764832e+001
1.899540e+003	7.771915e+001
1.901468e+003	7.779120e+001
1.903397e+003	7.776665e+001
1.905325e+003	7.772452e+001
1.907254e+003	7.754547e+001
1.909182e+003	7.742744e+001
1.911110e+003	7.736544e+001
1.913039e+003	7.738024e+001
1.914967e+003	7.749253e+001
1.916896e+003	7.758720e+001
1.918824e+003	7.760546e+001
1.920753e+003	7.739590e+001
1.922681e+003	7.726294e+001
1.924610e+003	7.739729e+001
1.926538e+003	7.736796e+001
1.928467e+003	7.746279e+001
1.930395e+003	7.748994e+001
1.932324e+003	7.749089e+001
1.934252e+003	7.745566e+001
1.936181e+003	7.762649e+001
1.938109e+003	7.756062e+001
1.940037e+003	7.709972e+001

1.941966e+003	7.686211e+001
1.943894e+003	7.727174e+001
1.945823e+003	7.733146e+001
1.947751e+003	7.742186e+001
1.949680e+003	7.760720e+001
1.951608e+003	7.781058e+001
1.953537e+003	7.766299e+001
1.955465e+003	7.763836e+001
1.957394e+003	7.766860e+001
1.959322e+003	7.774741e+001
1.961251e+003	7.784604e+001
1.963179e+003	7.788364e+001
1.965108e+003	7.755924e+001
1.967036e+003	7.777238e+001
1.968964e+003	7.809258e+001
1.970893e+003	7.815773e+001
1.972821e+003	7.768810e+001
1.974750e+003	7.769881e+001
1.976678e+003	7.749642e+001
1.978607e+003	7.774723e+001
1.980535e+003	7.787403e+001
1.982464e+003	7.763905e+001
1.984392e+003	7.729878e+001
1.986321e+003	7.723722e+001
1.988249e+003	7.716057e+001
1.990178e+003	7.709776e+001
1.992106e+003	7.717210e+001
1.994035e+003	7.737991e+001
1.995963e+003	7.738869e+001
1.997891e+003	7.757487e+001
1.999820e+003	7.763955e+001
2.001748e+003	7.774480e+001
2.003677e+003	7.791929e+001
2.005605e+003	7.779820e+001
2.007534e+003	7.765874e+001
2.009462e+003	7.778596e+001
2.011391e+003	7.738161e+001
2.013319e+003	7.736552e+001
2.015248e+003	7.736352e+001
2.017176e+003	7.738828e+001
2.019105e+003	7.765653e+001
2.021033e+003	7.769624e+001
2.022962e+003	7.723971e+001
2.024890e+003	7.772969e+001
2.026818e+003	7.794597e+001
2.028747e+003	7.734771e+001
2.030675e+003	7.685844e+001
2.032604e+003	7.693460e+001
2.034532e+003	7.662294e+001
2.036461e+003	7.677760e+001

2.038389e+003	7.696259e+001
2.040318e+003	7.714441e+001
2.042246e+003	7.695354e+001
2.044175e+003	7.679827e+001
2.046103e+003	7.645217e+001
2.048032e+003	7.601569e+001
2.049960e+003	7.594579e+001
2.051889e+003	7.602023e+001
2.053817e+003	7.597766e+001
2.055746e+003	7.597717e+001
2.057674e+003	7.612664e+001
2.059603e+003	7.629016e+001
2.061531e+003	7.610950e+001
2.063459e+003	7.578715e+001
2.065388e+003	7.586653e+001
2.067316e+003	7.586452e+001
2.069245e+003	7.565105e+001
2.071173e+003	7.584519e+001
2.073102e+003	7.583170e+001
2.075030e+003	7.552208e+001
2.076959e+003	7.517024e+001
2.078887e+003	7.513461e+001
2.080816e+003	7.515143e+001
2.082744e+003	7.527838e+001
2.084673e+003	7.519968e+001
2.086601e+003	7.520489e+001
2.088530e+003	7.511965e+001
2.090458e+003	7.492425e+001
2.092386e+003	7.496532e+001
2.094315e+003	7.503724e+001
2.096243e+003	7.489060e+001
2.098172e+003	7.502120e+001
2.100100e+003	7.493556e+001
2.102029e+003	7.491743e+001
2.103957e+003	7.498142e+001
2.105886e+003	7.511653e+001
2.107814e+003	7.500126e+001
2.109743e+003	7.473940e+001
2.111671e+003	7.468324e+001
2.113600e+003	7.482520e+001
2.115528e+003	7.484701e+001
2.117457e+003	7.485938e+001
2.119385e+003	7.494856e+001
2.121313e+003	7.495573e+001
2.123242e+003	7.485027e+001
2.125170e+003	7.486743e+001
2.127099e+003	7.484606e+001
2.129027e+003	7.475405e+001
2.130956e+003	7.482480e+001
2.132884e+003	7.506318e+001

2.134813e+003	7.508832e+001
2.136741e+003	7.486549e+001
2.138670e+003	7.491312e+001
2.140598e+003	7.512440e+001
2.142527e+003	7.492210e+001
2.144455e+003	7.482609e+001
2.146384e+003	7.533073e+001
2.148312e+003	7.556641e+001
2.150240e+003	7.556978e+001
2.152169e+003	7.563036e+001
2.154097e+003	7.572134e+001
2.156026e+003	7.582912e+001
2.157954e+003	7.545695e+001
2.159883e+003	7.573515e+001
2.161811e+003	7.574403e+001
2.163740e+003	7.638456e+001
2.165668e+003	7.680257e+001
2.167597e+003	7.595731e+001
2.169525e+003	7.514909e+001
2.171454e+003	7.532840e+001
2.173382e+003	7.515718e+001
2.175311e+003	7.513442e+001
2.177239e+003	7.532308e+001
2.179167e+003	7.532050e+001
2.181096e+003	7.494919e+001
2.183024e+003	7.493942e+001
2.184953e+003	7.498207e+001
2.186881e+003	7.482038e+001
2.188810e+003	7.451929e+001
2.190738e+003	7.432246e+001
2.192667e+003	7.432198e+001
2.194595e+003	7.447553e+001
2.196524e+003	7.428043e+001
2.198452e+003	7.427127e+001
2.200381e+003	7.434885e+001
2.202309e+003	7.426692e+001
2.204238e+003	7.395311e+001
2.206166e+003	7.394154e+001
2.208094e+003	7.414975e+001
2.210023e+003	7.451917e+001
2.211951e+003	7.426630e+001
2.213880e+003	7.382420e+001
2.215808e+003	7.382578e+001
2.217737e+003	7.402686e+001
2.219665e+003	7.407919e+001
2.221594e+003	7.385204e+001
2.223522e+003	7.367306e+001
2.225451e+003	7.351460e+001
2.227379e+003	7.379657e+001
2.229308e+003	7.381121e+001

2.231236e+003	7.351062e+001
2.233165e+003	7.341595e+001
2.235093e+003	7.328320e+001
2.237021e+003	7.330429e+001
2.238950e+003	7.330590e+001
2.240878e+003	7.340521e+001
2.242807e+003	7.360487e+001
2.244735e+003	7.352449e+001
2.246664e+003	7.333184e+001
2.248592e+003	7.315998e+001
2.250521e+003	7.313239e+001
2.252449e+003	7.322933e+001
2.254378e+003	7.318035e+001
2.256306e+003	7.323298e+001
2.258235e+003	7.311639e+001
2.260163e+003	7.309761e+001
2.262092e+003	7.301363e+001
2.264020e+003	7.275777e+001
2.265948e+003	7.263972e+001
2.267877e+003	7.277116e+001
2.269805e+003	7.287430e+001
2.271734e+003	7.280443e+001
2.273662e+003	7.261729e+001
2.275591e+003	7.257456e+001
2.277519e+003	7.253857e+001
2.279448e+003	7.249506e+001
2.281376e+003	7.248714e+001
2.283305e+003	7.249464e+001
2.285233e+003	7.239422e+001
2.287162e+003	7.236655e+001
2.289090e+003	7.229593e+001
2.291019e+003	7.221159e+001
2.292947e+003	7.217042e+001
2.294875e+003	7.219732e+001
2.296804e+003	7.221481e+001
2.298732e+003	7.215362e+001
2.300661e+003	7.212448e+001
2.302589e+003	7.214395e+001
2.304518e+003	7.207448e+001
2.306446e+003	7.197348e+001
2.308375e+003	7.188394e+001
2.310303e+003	7.192835e+001
2.312232e+003	7.197012e+001
2.314160e+003	7.188657e+001
2.316089e+003	7.176823e+001
2.318017e+003	7.180217e+001
2.319946e+003	7.172799e+001
2.321874e+003	7.160028e+001
2.323802e+003	7.144407e+001
2.325731e+003	7.123837e+001

2.327659e+003	7.104026e+001
2.329588e+003	7.092773e+001
2.331516e+003	7.119539e+001
2.333445e+003	7.145663e+001
2.335373e+003	7.136966e+001
2.337302e+003	7.134097e+001
2.339230e+003	7.140808e+001
2.341159e+003	7.147282e+001
2.343087e+003	7.147648e+001
2.345016e+003	7.134429e+001
2.346944e+003	7.122818e+001
2.348873e+003	7.122226e+001
2.350801e+003	7.124296e+001
2.352729e+003	7.110890e+001
2.354658e+003	7.107861e+001
2.356586e+003	7.115151e+001
2.358515e+003	7.115416e+001
2.360443e+003	7.113721e+001
2.362372e+003	7.119316e+001
2.364300e+003	7.117887e+001
2.366229e+003	7.128399e+001
2.368157e+003	7.125025e+001
2.370086e+003	7.104179e+001
2.372014e+003	7.105967e+001
2.373942e+003	7.112167e+001
2.375871e+003	7.109531e+001
2.377799e+003	7.103324e+001
2.379728e+003	7.090046e+001
2.381656e+003	7.084106e+001
2.383585e+003	7.088322e+001
2.385513e+003	7.084732e+001
2.387442e+003	7.082629e+001
2.389370e+003	7.079202e+001
2.391299e+003	7.074844e+001
2.393227e+003	7.079066e+001
2.395156e+003	7.077908e+001
2.397084e+003	7.070721e+001
2.399012e+003	7.066253e+001
2.400941e+003	7.061963e+001
2.402869e+003	7.061754e+001
2.404798e+003	7.068703e+001
2.406726e+003	7.070982e+001
2.408655e+003	7.071501e+001
2.410583e+003	7.068269e+001
2.412512e+003	7.064425e+001
2.414440e+003	7.066403e+001
2.416369e+003	7.055933e+001
2.418297e+003	7.045991e+001
2.420226e+003	7.041393e+001
2.422154e+003	7.040566e+001

2.424083e+003	7.045039e+001
2.426011e+003	7.042461e+001
2.427939e+003	7.036982e+001
2.429868e+003	7.037858e+001
2.431796e+003	7.034638e+001
2.433725e+003	7.028919e+001
2.435653e+003	7.024517e+001
2.437582e+003	7.025631e+001
2.439510e+003	7.026544e+001
2.441439e+003	7.031938e+001
2.443367e+003	7.030153e+001
2.445296e+003	7.029287e+001
2.447224e+003	7.019987e+001
2.449153e+003	7.016202e+001
2.451081e+003	7.021320e+001
2.453010e+003	7.016055e+001
2.454938e+003	7.016828e+001
2.456866e+003	7.018830e+001
2.458795e+003	7.008673e+001
2.460723e+003	7.001155e+001
2.462652e+003	7.004461e+001
2.464580e+003	7.012679e+001
2.466509e+003	7.009971e+001
2.468437e+003	7.003089e+001
2.470366e+003	6.997239e+001
2.472294e+003	6.994035e+001
2.474223e+003	7.002312e+001
2.476151e+003	7.001504e+001
2.478080e+003	6.991109e+001
2.480008e+003	6.994718e+001
2.481937e+003	6.989345e+001
2.483865e+003	6.981279e+001
2.485793e+003	6.984956e+001
2.487722e+003	6.983358e+001
2.489650e+003	6.973695e+001
2.491579e+003	6.973288e+001
2.493507e+003	6.975404e+001
2.495436e+003	6.966041e+001
2.497364e+003	6.959190e+001
2.499293e+003	6.967751e+001
2.501221e+003	6.972410e+001
2.503150e+003	6.965748e+001
2.505078e+003	6.964530e+001
2.507007e+003	6.960638e+001
2.508935e+003	6.955086e+001
2.510864e+003	6.955508e+001
2.512792e+003	6.947447e+001
2.514720e+003	6.946284e+001
2.516649e+003	6.948080e+001
2.518577e+003	6.949302e+001

2.520506e+003	6.947176e+001
2.522434e+003	6.939832e+001
2.524363e+003	6.934153e+001
2.526291e+003	6.935290e+001
2.528220e+003	6.937717e+001
2.530148e+003	6.942580e+001
2.532077e+003	6.938985e+001
2.534005e+003	6.929285e+001
2.535934e+003	6.923876e+001
2.537862e+003	6.926859e+001
2.539791e+003	6.924021e+001
2.541719e+003	6.919061e+001
2.543647e+003	6.914804e+001
2.545576e+003	6.908426e+001
2.547504e+003	6.907434e+001
2.549433e+003	6.914577e+001
2.551361e+003	6.914898e+001
2.553290e+003	6.909939e+001
2.555218e+003	6.908182e+001
2.557147e+003	6.904157e+001
2.559075e+003	6.898114e+001
2.561004e+003	6.892385e+001
2.562932e+003	6.888602e+001
2.564861e+003	6.888062e+001
2.566789e+003	6.889796e+001
2.568718e+003	6.890138e+001
2.570646e+003	6.886015e+001
2.572574e+003	6.873325e+001
2.574503e+003	6.871377e+001
2.576431e+003	6.877187e+001
2.578360e+003	6.879661e+001
2.580288e+003	6.875741e+001
2.582217e+003	6.869433e+001
2.584145e+003	6.864509e+001
2.586074e+003	6.858734e+001
2.588002e+003	6.857776e+001
2.589931e+003	6.857013e+001
2.591859e+003	6.852921e+001
2.593788e+003	6.849030e+001
2.595716e+003	6.853089e+001
2.597645e+003	6.858088e+001
2.599573e+003	6.847605e+001
2.601501e+003	6.833602e+001
2.603430e+003	6.829067e+001
2.605358e+003	6.833074e+001
2.607287e+003	6.828890e+001
2.609215e+003	6.822499e+001
2.611144e+003	6.821171e+001
2.613072e+003	6.817963e+001
2.615001e+003	6.819198e+001

2.616929e+003	6.815392e+001
2.618858e+003	6.808926e+001
2.620786e+003	6.803159e+001
2.622715e+003	6.799122e+001
2.624643e+003	6.795922e+001
2.626572e+003	6.792511e+001
2.628500e+003	6.790874e+001
2.630428e+003	6.786820e+001
2.632357e+003	6.780923e+001
2.634285e+003	6.776870e+001
2.636214e+003	6.770747e+001
2.638142e+003	6.768971e+001
2.640071e+003	6.767228e+001
2.641999e+003	6.760252e+001
2.643928e+003	6.752460e+001
2.645856e+003	6.745600e+001
2.647785e+003	6.742275e+001
2.649713e+003	6.737280e+001
2.651642e+003	6.728947e+001
2.653570e+003	6.728979e+001
2.655499e+003	6.727140e+001
2.657427e+003	6.719163e+001
2.659355e+003	6.714114e+001
2.661284e+003	6.711484e+001
2.663212e+003	6.707468e+001
2.665141e+003	6.705202e+001
2.667069e+003	6.704479e+001
2.668998e+003	6.702340e+001
2.670926e+003	6.697131e+001
2.672855e+003	6.695963e+001
2.674783e+003	6.695347e+001
2.676712e+003	6.691713e+001
2.678640e+003	6.688412e+001
2.680569e+003	6.687977e+001
2.682497e+003	6.686724e+001
2.684426e+003	6.684180e+001
2.686354e+003	6.685431e+001
2.688282e+003	6.685800e+001
2.690211e+003	6.677863e+001
2.692139e+003	6.674695e+001
2.694068e+003	6.674769e+001
2.695996e+003	6.673329e+001
2.697925e+003	6.669907e+001
2.699853e+003	6.668849e+001
2.701782e+003	6.671197e+001
2.703710e+003	6.670489e+001
2.705639e+003	6.667266e+001
2.707567e+003	6.662031e+001
2.709496e+003	6.658561e+001
2.711424e+003	6.658203e+001

2.713353e+003	6.657864e+001
2.715281e+003	6.656510e+001
2.717209e+003	6.652907e+001
2.719138e+003	6.650553e+001
2.721066e+003	6.648636e+001
2.722995e+003	6.648354e+001
2.724923e+003	6.650650e+001
2.726852e+003	6.646522e+001
2.728780e+003	6.642536e+001
2.730709e+003	6.642709e+001
2.732637e+003	6.643320e+001
2.734566e+003	6.637416e+001
2.736494e+003	6.634746e+001
2.738423e+003	6.633569e+001
2.740351e+003	6.632692e+001
2.742280e+003	6.632365e+001
2.744208e+003	6.629355e+001
2.746136e+003	6.630239e+001
2.748065e+003	6.630785e+001
2.749993e+003	6.624664e+001
2.751922e+003	6.620453e+001
2.753850e+003	6.615703e+001
2.755779e+003	6.614693e+001
2.757707e+003	6.615913e+001
2.759636e+003	6.617108e+001
2.761564e+003	6.614726e+001
2.763493e+003	6.611592e+001
2.765421e+003	6.610535e+001
2.767350e+003	6.610589e+001
2.769278e+003	6.606446e+001
2.771207e+003	6.600649e+001
2.773135e+003	6.596432e+001
2.775063e+003	6.596141e+001
2.776992e+003	6.597243e+001
2.778920e+003	6.599979e+001
2.780849e+003	6.598792e+001
2.782777e+003	6.593481e+001
2.784706e+003	6.589940e+001
2.786634e+003	6.586799e+001
2.788563e+003	6.585700e+001
2.790491e+003	6.587357e+001
2.792420e+003	6.586450e+001
2.794348e+003	6.581553e+001
2.796277e+003	6.579870e+001
2.798205e+003	6.575879e+001
2.800134e+003	6.573391e+001
2.802062e+003	6.573679e+001
2.803990e+003	6.573657e+001
2.805919e+003	6.573126e+001
2.807847e+003	6.573087e+001

2.809776e+003	6.570992e+001
2.811704e+003	6.566438e+001
2.813633e+003	6.564945e+001
2.815561e+003	6.563856e+001
2.817490e+003	6.559852e+001
2.819418e+003	6.558691e+001
2.821347e+003	6.558322e+001
2.823275e+003	6.555556e+001
2.825204e+003	6.557053e+001
2.827132e+003	6.555598e+001
2.829061e+003	6.552061e+001
2.830989e+003	6.548375e+001
2.832917e+003	6.543402e+001
2.834846e+003	6.542357e+001
2.836774e+003	6.544818e+001
2.838703e+003	6.545286e+001
2.840631e+003	6.546622e+001
2.842560e+003	6.548608e+001
2.844488e+003	6.550510e+001
2.846417e+003	6.549242e+001
2.848345e+003	6.553828e+001
2.850274e+003	6.561257e+001
2.852202e+003	6.561891e+001
2.854131e+003	6.562792e+001
2.856059e+003	6.563955e+001
2.857988e+003	6.558853e+001
2.859916e+003	6.551400e+001
2.861844e+003	6.548550e+001
2.863773e+003	6.543604e+001
2.865701e+003	6.538628e+001
2.867630e+003	6.536503e+001
2.869558e+003	6.535588e+001
2.871487e+003	6.533574e+001
2.873415e+003	6.530056e+001
2.875344e+003	6.525714e+001
2.877272e+003	6.525022e+001
2.879201e+003	6.524944e+001
2.881129e+003	6.525467e+001
2.883058e+003	6.526308e+001
2.884986e+003	6.526118e+001
2.886915e+003	6.525887e+001
2.888843e+003	6.521716e+001
2.890771e+003	6.515988e+001
2.892700e+003	6.516534e+001
2.894628e+003	6.512028e+001
2.896557e+003	6.507537e+001
2.898485e+003	6.513298e+001
2.900414e+003	6.514461e+001
2.902342e+003	6.515506e+001
2.904271e+003	6.517138e+001

2.906199e+003	6.518395e+001
2.908128e+003	6.518799e+001
2.910056e+003	6.510879e+001
2.911985e+003	6.508855e+001
2.913913e+003	6.519296e+001
2.915842e+003	6.527620e+001
2.917770e+003	6.529690e+001
2.919698e+003	6.536445e+001
2.921627e+003	6.540410e+001
2.923555e+003	6.532065e+001
2.925484e+003	6.532638e+001
2.927412e+003	6.537936e+001
2.929341e+003	6.532213e+001
2.931269e+003	6.525193e+001
2.933198e+003	6.521152e+001
2.935126e+003	6.520647e+001
2.937055e+003	6.519213e+001
2.938983e+003	6.516800e+001
2.940912e+003	6.510220e+001
2.942840e+003	6.503528e+001
2.944769e+003	6.501015e+001
2.946697e+003	6.497410e+001
2.948625e+003	6.495981e+001
2.950554e+003	6.491749e+001
2.952482e+003	6.487114e+001
2.954411e+003	6.485262e+001
2.956339e+003	6.486028e+001
2.958268e+003	6.487150e+001
2.960196e+003	6.485902e+001
2.962125e+003	6.483249e+001
2.964053e+003	6.483108e+001
2.965982e+003	6.483490e+001
2.967910e+003	6.482914e+001
2.969839e+003	6.481650e+001
2.971767e+003	6.475333e+001
2.973696e+003	6.468826e+001
2.975624e+003	6.467683e+001
2.977552e+003	6.467941e+001
2.979481e+003	6.464066e+001
2.981409e+003	6.459336e+001
2.983338e+003	6.456598e+001
2.985266e+003	6.456862e+001
2.987195e+003	6.451456e+001
2.989123e+003	6.447929e+001
2.991052e+003	6.445765e+001
2.992980e+003	6.443535e+001
2.994909e+003	6.444470e+001
2.996837e+003	6.447456e+001
2.998766e+003	6.445281e+001
3.000694e+003	6.438658e+001

3.002623e+003	6.432212e+001
3.004551e+003	6.431683e+001
3.006479e+003	6.436466e+001
3.008408e+003	6.431510e+001
3.010336e+003	6.422375e+001
3.012265e+003	6.422184e+001
3.014193e+003	6.424689e+001
3.016122e+003	6.421838e+001
3.018050e+003	6.410760e+001
3.019979e+003	6.406580e+001
3.021907e+003	6.410394e+001
3.023836e+003	6.413544e+001
3.025764e+003	6.416687e+001
3.027693e+003	6.414648e+001
3.029621e+003	6.409524e+001
3.031550e+003	6.404700e+001
3.033478e+003	6.401058e+001
3.035406e+003	6.398809e+001
3.037335e+003	6.395663e+001
3.039263e+003	6.393431e+001
3.041192e+003	6.392978e+001
3.043120e+003	6.394281e+001
3.045049e+003	6.390705e+001
3.046977e+003	6.389445e+001
3.048906e+003	6.391617e+001
3.050834e+003	6.389727e+001
3.052763e+003	6.389490e+001
3.054691e+003	6.388580e+001
3.056620e+003	6.386741e+001
3.058548e+003	6.380939e+001
3.060477e+003	6.373347e+001
3.062405e+003	6.368502e+001
3.064333e+003	6.366015e+001
3.066262e+003	6.366425e+001
3.068190e+003	6.369144e+001
3.070119e+003	6.366570e+001
3.072047e+003	6.367073e+001
3.073976e+003	6.370972e+001
3.075904e+003	6.364678e+001
3.077833e+003	6.354294e+001
3.079761e+003	6.351860e+001
3.081690e+003	6.353844e+001
3.083618e+003	6.354668e+001
3.085547e+003	6.354539e+001
3.087475e+003	6.352775e+001
3.089404e+003	6.345483e+001
3.091332e+003	6.339331e+001
3.093260e+003	6.341633e+001
3.095189e+003	6.344122e+001
3.097117e+003	6.344830e+001

3.099046e+003	6.343615e+001
3.100974e+003	6.336982e+001
3.102903e+003	6.335000e+001
3.104831e+003	6.339846e+001
3.106760e+003	6.338972e+001
3.108688e+003	6.332331e+001
3.110617e+003	6.326782e+001
3.112545e+003	6.329273e+001
3.114474e+003	6.332807e+001
3.116402e+003	6.332032e+001
3.118331e+003	6.332022e+001
3.120259e+003	6.330708e+001
3.122188e+003	6.326075e+001
3.124116e+003	6.325177e+001
3.126044e+003	6.322403e+001
3.127973e+003	6.320009e+001
3.129901e+003	6.313278e+001
3.131830e+003	6.310905e+001
3.133758e+003	6.314460e+001
3.135687e+003	6.311836e+001
3.137615e+003	6.308250e+001
3.139544e+003	6.307798e+001
3.141472e+003	6.306156e+001
3.143401e+003	6.307820e+001
3.145329e+003	6.306128e+001
3.147258e+003	6.302423e+001
3.149186e+003	6.302444e+001
3.151115e+003	6.302178e+001
3.153043e+003	6.300075e+001
3.154971e+003	6.303856e+001
3.156900e+003	6.302315e+001
3.158828e+003	6.302028e+001
3.160757e+003	6.299546e+001
3.162685e+003	6.301891e+001
3.164614e+003	6.306476e+001
3.166542e+003	6.300077e+001
3.168471e+003	6.292927e+001
3.170399e+003	6.286673e+001
3.172328e+003	6.279386e+001
3.174256e+003	6.275512e+001
3.176185e+003	6.279311e+001
3.178113e+003	6.279823e+001
3.180042e+003	6.277067e+001
3.181970e+003	6.278629e+001
3.183898e+003	6.278331e+001
3.185827e+003	6.277457e+001
3.187755e+003	6.272525e+001
3.189684e+003	6.272697e+001
3.191612e+003	6.270903e+001
3.193541e+003	6.270044e+001

3.195469e+003	6.268541e+001
3.197398e+003	6.264872e+001
3.199326e+003	6.261409e+001
3.201255e+003	6.261684e+001
3.203183e+003	6.260167e+001
3.205112e+003	6.258561e+001
3.207040e+003	6.260143e+001
3.208969e+003	6.261460e+001
3.210897e+003	6.258265e+001
3.212825e+003	6.254634e+001
3.214754e+003	6.255119e+001
3.216682e+003	6.253115e+001
3.218611e+003	6.239610e+001
3.220539e+003	6.242817e+001
3.222468e+003	6.250700e+001
3.224396e+003	6.248960e+001
3.226325e+003	6.250424e+001
3.228253e+003	6.243831e+001
3.230182e+003	6.239291e+001
3.232110e+003	6.234952e+001
3.234039e+003	6.235114e+001
3.235967e+003	6.235083e+001
3.237896e+003	6.238349e+001
3.239824e+003	6.234244e+001
3.241752e+003	6.228247e+001
3.243681e+003	6.228999e+001
3.245609e+003	6.231020e+001
3.247538e+003	6.230055e+001
3.249466e+003	6.224220e+001
3.251395e+003	6.221714e+001
3.253323e+003	6.218375e+001
3.255252e+003	6.220872e+001
3.257180e+003	6.222292e+001
3.259109e+003	6.217678e+001
3.261037e+003	6.216438e+001
3.262966e+003	6.217378e+001
3.264894e+003	6.215127e+001
3.266823e+003	6.210816e+001
3.268751e+003	6.207920e+001
3.270679e+003	6.201968e+001
3.272608e+003	6.202924e+001
3.274536e+003	6.204300e+001
3.276465e+003	6.199880e+001
3.278393e+003	6.195444e+001
3.280322e+003	6.197072e+001
3.282250e+003	6.203730e+001
3.284179e+003	6.203024e+001
3.286107e+003	6.198615e+001
3.288036e+003	6.196891e+001
3.289964e+003	6.191394e+001

3.291893e+003	6.186366e+001
3.293821e+003	6.187663e+001
3.295750e+003	6.187101e+001
3.297678e+003	6.190649e+001
3.299606e+003	6.190425e+001
3.301535e+003	6.189483e+001
3.303463e+003	6.187493e+001
3.305392e+003	6.182772e+001
3.307320e+003	6.177943e+001
3.309249e+003	6.173730e+001
3.311177e+003	6.172353e+001
3.313106e+003	6.178289e+001
3.315034e+003	6.180994e+001
3.316963e+003	6.175240e+001
3.318891e+003	6.172075e+001
3.320820e+003	6.168795e+001
3.322748e+003	6.167841e+001
3.324677e+003	6.165957e+001
3.326605e+003	6.165421e+001
3.328533e+003	6.162157e+001
3.330462e+003	6.157656e+001
3.332390e+003	6.155399e+001
3.334319e+003	6.150915e+001
3.336247e+003	6.150137e+001
3.338176e+003	6.152178e+001
3.340104e+003	6.152968e+001
3.342033e+003	6.154577e+001
3.343961e+003	6.152380e+001
3.345890e+003	6.158004e+001
3.347818e+003	6.159641e+001
3.349747e+003	6.157447e+001
3.351675e+003	6.155700e+001
3.353604e+003	6.146641e+001
3.355532e+003	6.144510e+001
3.357460e+003	6.145424e+001
3.359389e+003	6.139057e+001
3.361317e+003	6.140602e+001
3.363246e+003	6.146601e+001
3.365174e+003	6.143083e+001
3.367103e+003	6.139011e+001
3.369031e+003	6.138382e+001
3.370960e+003	6.130957e+001
3.372888e+003	6.134540e+001
3.374817e+003	6.143185e+001
3.376745e+003	6.140998e+001
3.378674e+003	6.139915e+001
3.380602e+003	6.138859e+001
3.382531e+003	6.138702e+001
3.384459e+003	6.134262e+001
3.386387e+003	6.131026e+001

3.388316e+003	6.130570e+001
3.390244e+003	6.126330e+001
3.392173e+003	6.124220e+001
3.394101e+003	6.128398e+001
3.396030e+003	6.130447e+001
3.397958e+003	6.124901e+001
3.399887e+003	6.122782e+001
3.401815e+003	6.125371e+001
3.403744e+003	6.125970e+001
3.405672e+003	6.129196e+001
3.407601e+003	6.130375e+001
3.409529e+003	6.129435e+001
3.411458e+003	6.125259e+001
3.413386e+003	6.119430e+001
3.415314e+003	6.118776e+001
3.417243e+003	6.119405e+001
3.419171e+003	6.114557e+001
3.421100e+003	6.119930e+001
3.423028e+003	6.126119e+001
3.424957e+003	6.128078e+001
3.426885e+003	6.128580e+001
3.428814e+003	6.123695e+001
3.430742e+003	6.116661e+001
3.432671e+003	6.118853e+001
3.434599e+003	6.116487e+001
3.436528e+003	6.115215e+001
3.438456e+003	6.123217e+001
3.440385e+003	6.118805e+001
3.442313e+003	6.109896e+001
3.444241e+003	6.112251e+001
3.446170e+003	6.108334e+001
3.448098e+003	6.110973e+001
3.450027e+003	6.116253e+001
3.451955e+003	6.118195e+001
3.453884e+003	6.123029e+001
3.455812e+003	6.118827e+001
3.457741e+003	6.110833e+001
3.459669e+003	6.114861e+001
3.461598e+003	6.114310e+001
3.463526e+003	6.110622e+001
3.465455e+003	6.109649e+001
3.467383e+003	6.108464e+001
3.469312e+003	6.105880e+001
3.471240e+003	6.106510e+001
3.473168e+003	6.108408e+001
3.475097e+003	6.106220e+001
3.477025e+003	6.103616e+001
3.478954e+003	6.096604e+001
3.480882e+003	6.093155e+001
3.482811e+003	6.093297e+001

3.484739e+003	6.102114e+001
3.486668e+003	6.107593e+001
3.488596e+003	6.103685e+001
3.490525e+003	6.103324e+001
3.492453e+003	6.103870e+001
3.494382e+003	6.092421e+001
3.496310e+003	6.084023e+001
3.498239e+003	6.085311e+001
3.500167e+003	6.083031e+001
3.502095e+003	6.078210e+001
3.504024e+003	6.082261e+001
3.505952e+003	6.085149e+001
3.507881e+003	6.081020e+001
3.509809e+003	6.081721e+001
3.511738e+003	6.083546e+001
3.513666e+003	6.094098e+001
3.515595e+003	6.097039e+001
3.517523e+003	6.090470e+001
3.519452e+003	6.092742e+001
3.521380e+003	6.087483e+001
3.523309e+003	6.085911e+001
3.525237e+003	6.081210e+001
3.527166e+003	6.076001e+001
3.529094e+003	6.081965e+001
3.531022e+003	6.084217e+001
3.532951e+003	6.077240e+001
3.534879e+003	6.073834e+001
3.536808e+003	6.074984e+001
3.538736e+003	6.079218e+001
3.540665e+003	6.077910e+001
3.542593e+003	6.069546e+001
3.544522e+003	6.066831e+001
3.546450e+003	6.079033e+001
3.548379e+003	6.083279e+001
3.550307e+003	6.070555e+001
3.552236e+003	6.065617e+001
3.554164e+003	6.069668e+001
3.556093e+003	6.070658e+001
3.558021e+003	6.072464e+001
3.559949e+003	6.069971e+001
3.561878e+003	6.072533e+001
3.563806e+003	6.066148e+001
3.565735e+003	6.056604e+001
3.567663e+003	6.068513e+001
3.569592e+003	6.068704e+001
3.571520e+003	6.065936e+001
3.573449e+003	6.075899e+001
3.575377e+003	6.078147e+001
3.577306e+003	6.066755e+001
3.579234e+003	6.069315e+001

3.581163e+003	6.073769e+001
3.583091e+003	6.066382e+001
3.585020e+003	6.051131e+001
3.586948e+003	6.045580e+001
3.588876e+003	6.067554e+001
3.590805e+003	6.070467e+001
3.592733e+003	6.061337e+001
3.594662e+003	6.051186e+001
3.596590e+003	6.058722e+001
3.598519e+003	6.057549e+001
3.600447e+003	6.051276e+001
3.602376e+003	6.051834e+001
3.604304e+003	6.046001e+001
3.606233e+003	6.037515e+001
3.608161e+003	6.047232e+001
3.610090e+003	6.051227e+001
3.612018e+003	6.041106e+001
3.613947e+003	6.028811e+001
3.615875e+003	6.030596e+001
3.617803e+003	6.038431e+001
3.619732e+003	6.044585e+001
3.621660e+003	6.045534e+001
3.623589e+003	6.042810e+001
3.625517e+003	6.041223e+001
3.627446e+003	6.046630e+001
3.629374e+003	6.043214e+001
3.631303e+003	6.033461e+001
3.633231e+003	6.042441e+001
3.635160e+003	6.048441e+001
3.637088e+003	6.041879e+001
3.639017e+003	6.041575e+001
3.640945e+003	6.035883e+001
3.642874e+003	6.026804e+001
3.644802e+003	6.025833e+001
3.646730e+003	6.012199e+001
3.648659e+003	5.996799e+001
3.650587e+003	6.007143e+001
3.652516e+003	6.018063e+001
3.654444e+003	6.028809e+001
3.656373e+003	6.030750e+001
3.658301e+003	6.017875e+001
3.660230e+003	6.013514e+001
3.662158e+003	6.018964e+001
3.664087e+003	6.016330e+001
3.666015e+003	6.007892e+001
3.667944e+003	6.014526e+001
3.669872e+003	6.028371e+001
3.671801e+003	6.007352e+001
3.673729e+003	5.987582e+001
3.675657e+003	5.989555e+001

3.677586e+003	5.984751e+001
3.679514e+003	5.990868e+001
3.681443e+003	5.994923e+001
3.683371e+003	5.991179e+001
3.685300e+003	5.984315e+001
3.687228e+003	5.974731e+001
3.689157e+003	5.968101e+001
3.691085e+003	5.981826e+001
3.693014e+003	5.984046e+001
3.694942e+003	5.978474e+001
3.696871e+003	5.982096e+001
3.698799e+003	5.987774e+001
3.700728e+003	5.978430e+001
3.702656e+003	5.971543e+001
3.704584e+003	5.970288e+001
3.706513e+003	5.972689e+001
3.708441e+003	5.969810e+001
3.710370e+003	5.961942e+001
3.712298e+003	5.960658e+001
3.714227e+003	5.958145e+001
3.716155e+003	5.959307e+001
3.718084e+003	5.953286e+001
3.720012e+003	5.953324e+001
3.721941e+003	5.947652e+001
3.723869e+003	5.941371e+001
3.725798e+003	5.946285e+001
3.727726e+003	5.953449e+001
3.729655e+003	5.957682e+001
3.731583e+003	5.954639e+001
3.733511e+003	5.942348e+001
3.735440e+003	5.936046e+001
3.737368e+003	5.916243e+001
3.739297e+003	5.902184e+001
3.741225e+003	5.913929e+001
3.743154e+003	5.929535e+001
3.745082e+003	5.916299e+001
3.747011e+003	5.911594e+001
3.748939e+003	5.920197e+001
3.750868e+003	5.912767e+001
3.752796e+003	5.912406e+001
3.754725e+003	5.902999e+001
3.756653e+003	5.892179e+001
3.758582e+003	5.888340e+001
3.760510e+003	5.905352e+001
3.762438e+003	5.905879e+001
3.764367e+003	5.906015e+001
3.766295e+003	5.898243e+001
3.768224e+003	5.884715e+001
3.770152e+003	5.889709e+001
3.772081e+003	5.902391e+001

3.774009e+003	5.904142e+001
3.775938e+003	5.900053e+001
3.777866e+003	5.895814e+001
3.779795e+003	5.897343e+001
3.781723e+003	5.884622e+001
3.783652e+003	5.871642e+001
3.785580e+003	5.872442e+001
3.787509e+003	5.878070e+001
3.789437e+003	5.875377e+001
3.791365e+003	5.869521e+001
3.793294e+003	5.865370e+001
3.795222e+003	5.860999e+001
3.797151e+003	5.849411e+001
3.799079e+003	5.840641e+001
3.801008e+003	5.839433e+001
3.802936e+003	5.846361e+001
3.804865e+003	5.841107e+001
3.806793e+003	5.841165e+001
3.808722e+003	5.848848e+001
3.810650e+003	5.854979e+001
3.812579e+003	5.846738e+001
3.814507e+003	5.843584e+001
3.816436e+003	5.845006e+001
3.818364e+003	5.835774e+001
3.820292e+003	5.832113e+001
3.822221e+003	5.837829e+001
3.824149e+003	5.833208e+001
3.826078e+003	5.833830e+001
3.828006e+003	5.831096e+001
3.829935e+003	5.827716e+001
3.831863e+003	5.821027e+001
3.833792e+003	5.805195e+001
3.835720e+003	5.802631e+001
3.837649e+003	5.803398e+001
3.839577e+003	5.796265e+001
3.841506e+003	5.805447e+001
3.843434e+003	5.807905e+001
3.845363e+003	5.810167e+001
3.847291e+003	5.807320e+001
3.849219e+003	5.806960e+001
3.851148e+003	5.817559e+001
3.853076e+003	5.835418e+001
3.855005e+003	5.811273e+001
3.856933e+003	5.806295e+001
3.858862e+003	5.813510e+001
3.860790e+003	5.796600e+001
3.862719e+003	5.782835e+001
3.864647e+003	5.787909e+001
3.866576e+003	5.788502e+001
3.868504e+003	5.783975e+001

3.870433e+003	5.780491e+001
3.872361e+003	5.783261e+001
3.874290e+003	5.792067e+001
3.876218e+003	5.788192e+001
3.878146e+003	5.785277e+001
3.880075e+003	5.776818e+001
3.882003e+003	5.765263e+001
3.883932e+003	5.772804e+001
3.885860e+003	5.793370e+001
3.887789e+003	5.780521e+001
3.889717e+003	5.763213e+001
3.891646e+003	5.749900e+001
3.893574e+003	5.765383e+001
3.895503e+003	5.768767e+001
3.897431e+003	5.767102e+001
3.899360e+003	5.771922e+001
3.901288e+003	5.764329e+001
3.903217e+003	5.747584e+001
3.905145e+003	5.748626e+001
3.907073e+003	5.757378e+001
3.909002e+003	5.765034e+001
3.910930e+003	5.763232e+001
3.912859e+003	5.756153e+001
3.914787e+003	5.753207e+001
3.916716e+003	5.758145e+001
3.918644e+003	5.752904e+001
3.920573e+003	5.750497e+001
3.922501e+003	5.747086e+001
3.924430e+003	5.744031e+001
3.926358e+003	5.750824e+001
3.928287e+003	5.745670e+001
3.930215e+003	5.738375e+001
3.932144e+003	5.739335e+001
3.934072e+003	5.735792e+001
3.936000e+003	5.740734e+001
3.937929e+003	5.745408e+001
3.939857e+003	5.735971e+001
3.941786e+003	5.730968e+001
3.943714e+003	5.736503e+001
3.945643e+003	5.733483e+001
3.947571e+003	5.722483e+001
3.949500e+003	5.723435e+001
3.951428e+003	5.733218e+001
3.953357e+003	5.728881e+001
3.955285e+003	5.723666e+001
3.957214e+003	5.718077e+001
3.959142e+003	5.711832e+001
3.961071e+003	5.716817e+001
3.962999e+003	5.722130e+001
3.964927e+003	5.721645e+001

3.966856e+003	5.721920e+001
3.968784e+003	5.719595e+001
3.970713e+003	5.713723e+001
3.972641e+003	5.707509e+001
3.974570e+003	5.708413e+001
3.976498e+003	5.711877e+001
3.978427e+003	5.716173e+001
3.980355e+003	5.715744e+001
3.982284e+003	5.714741e+001
3.984212e+003	5.710968e+001
3.986141e+003	5.707422e+001
3.988069e+003	5.707367e+001
3.989998e+003	5.711193e+001
3.991926e+003	5.711992e+001
3.993854e+003	5.705966e+001
3.995783e+003	5.694498e+001
3.997711e+003	5.689953e+001
3.999640e+003	5.695874e+001
4.001568e+003	0.000000e+000
