# Supplementary material for: Synthesis of 1D Bi2O3 nanostructures from hybrid electrospun fibrous mats and their morphology, structure, optical and electrical properties
Source: Sci Rep. 2022 Mar 8;12:4046. doi: 10.1038/s41598-022-07830-z (PMC8904472; doi:10.1038/s41598-022-07830-z)
Supplement: Supplementary file 2 — Supplementary Information 2. [file 41598_2022_7830_MOESM2_ESM.pdf]

3.991926e+002	8.692548e+001
4.011211e+002	8.735826e+001
4.030496e+002	8.763062e+001
4.049780e+002	8.732893e+001
4.069065e+002	8.715771e+001
4.088350e+002	8.704961e+001
4.107634e+002	8.697151e+001
4.126919e+002	8.693985e+001
4.146204e+002	8.688741e+001
4.165488e+002	8.686444e+001
4.184773e+002	8.677306e+001
4.204058e+002	8.654849e+001
4.223342e+002	8.656989e+001
4.242627e+002	8.669187e+001
4.261912e+002	8.670750e+001
4.281196e+002	8.675354e+001
4.300481e+002	8.668240e+001
4.319766e+002	8.652880e+001
4.339050e+002	8.657819e+001
4.358335e+002	8.655738e+001
4.377620e+002	8.646529e+001
4.396904e+002	8.641971e+001
4.416189e+002	8.642165e+001
4.435474e+002	8.641264e+001
4.454758e+002	8.638516e+001
4.474043e+002	8.621622e+001
4.493328e+002	8.623082e+001
4.512612e+002	8.621786e+001
4.531897e+002	8.628850e+001
4.551182e+002	8.624204e+001
4.570466e+002	8.619040e+001
4.589751e+002	8.619674e+001
4.609036e+002	8.621775e+001
4.628320e+002	8.617398e+001
4.647605e+002	8.623202e+001
4.666890e+002	8.623385e+001
4.686174e+002	8.625757e+001
4.705459e+002	8.628565e+001
4.724744e+002	8.632461e+001
4.744028e+002	8.624766e+001
4.763313e+002	8.618221e+001
4.782598e+002	8.620837e+001
4.801882e+002	8.618324e+001
4.821167e+002	8.618385e+001
4.840452e+002	8.631542e+001
4.859736e+002	8.632270e+001
4.879021e+002	8.634293e+001
4.898306e+002	8.634890e+001
4.917590e+002	8.627970e+001
4.936875e+002	8.627009e+001

4.956160e+002	8.630714e+001
4.975444e+002	8.627840e+001
4.994729e+002	8.629447e+001
5.014014e+002	8.630758e+001
5.033298e+002	8.637027e+001
5.052583e+002	8.644154e+001
5.071868e+002	8.637002e+001
5.091152e+002	8.628014e+001
5.110437e+002	8.628387e+001
5.129722e+002	8.630395e+001
5.149006e+002	8.638047e+001
5.168291e+002	8.639546e+001
5.187576e+002	8.641277e+001
5.206860e+002	8.634683e+001
5.226145e+002	8.639603e+001
5.245430e+002	8.645827e+001
5.264714e+002	8.643816e+001
5.283999e+002	8.636495e+001
5.303284e+002	8.638314e+001
5.322568e+002	8.639891e+001
5.341853e+002	8.648634e+001
5.361138e+002	8.651863e+001
5.380422e+002	8.651463e+001
5.399707e+002	8.654579e+001
5.418992e+002	8.660062e+001
5.438276e+002	8.652989e+001
5.457561e+002	8.648441e+001
5.476846e+002	8.649416e+001
5.496130e+002	8.656609e+001
5.515415e+002	8.659314e+001
5.534700e+002	8.662826e+001
5.553984e+002	8.664691e+001
5.573269e+002	8.661311e+001
5.592554e+002	8.657767e+001
5.611838e+002	8.666106e+001
5.631123e+002	8.669483e+001
5.650408e+002	8.672349e+001
5.669692e+002	8.675739e+001
5.688977e+002	8.676619e+001
5.708262e+002	8.678433e+001
5.727546e+002	8.682442e+001
5.746831e+002	8.684800e+001
5.766116e+002	8.685512e+001
5.785400e+002	8.686347e+001
5.804685e+002	8.690432e+001
5.823970e+002	8.696416e+001
5.843254e+002	8.703036e+001
5.862539e+002	8.698923e+001
5.881824e+002	8.698672e+001
5.901108e+002	8.699352e+001

5.920393e+002	8.702557e+001
5.939678e+002	8.704041e+001
5.958962e+002	8.704751e+001
5.978247e+002	8.705909e+001
5.997532e+002	8.710416e+001
6.016816e+002	8.714680e+001
6.036101e+002	8.717461e+001
6.055386e+002	8.716438e+001
6.074670e+002	8.717070e+001
6.093955e+002	8.717310e+001
6.113240e+002	8.722730e+001
6.132524e+002	8.726418e+001
6.151809e+002	8.728627e+001
6.171094e+002	8.733138e+001
6.190378e+002	8.736929e+001
6.209663e+002	8.740128e+001
6.228948e+002	8.747077e+001
6.248232e+002	8.753796e+001
6.267517e+002	8.758965e+001
6.286802e+002	8.761525e+001
6.306086e+002	8.765065e+001
6.325371e+002	8.767134e+001
6.344656e+002	8.771668e+001
6.363940e+002	8.771448e+001
6.383225e+002	8.773897e+001
6.402510e+002	8.776002e+001
6.421794e+002	8.776228e+001
6.441079e+002	8.780908e+001
6.460364e+002	8.784956e+001
6.479648e+002	8.786868e+001
6.498933e+002	8.793503e+001
6.518218e+002	8.793349e+001
6.537502e+002	8.791642e+001
6.556787e+002	8.791497e+001
6.576072e+002	8.792193e+001
6.595356e+002	8.794359e+001
6.614641e+002	8.795982e+001
6.633926e+002	8.796450e+001
6.653210e+002	8.793382e+001
6.672495e+002	8.788784e+001
6.691780e+002	8.799946e+001
6.711064e+002	8.803999e+001
6.730349e+002	8.802120e+001
6.749634e+002	8.798149e+001
6.768918e+002	8.793933e+001
6.788203e+002	8.795498e+001
6.807488e+002	8.800169e+001
6.826772e+002	8.802653e+001
6.846057e+002	8.800857e+001
6.865342e+002	8.799317e+001

6.884626e+002	8.799155e+001
6.903911e+002	8.798361e+001
6.923196e+002	8.798583e+001
6.942480e+002	8.797445e+001
6.961765e+002	8.797243e+001
6.981050e+002	8.796182e+001
7.000334e+002	8.797130e+001
7.019619e+002	8.795885e+001
7.038904e+002	8.792225e+001
7.058188e+002	8.787061e+001
7.077473e+002	8.782092e+001
7.096758e+002	8.778600e+001
7.116042e+002	8.777403e+001
7.135327e+002	8.775856e+001
7.154612e+002	8.775437e+001
7.173896e+002	8.776527e+001
7.193181e+002	8.774097e+001
7.212466e+002	8.771603e+001
7.231750e+002	8.773396e+001
7.251035e+002	8.772427e+001
7.270320e+002	8.768967e+001
7.289604e+002	8.765997e+001
7.308889e+002	8.763106e+001
7.328174e+002	8.758983e+001
7.347458e+002	8.756268e+001
7.366743e+002	8.753141e+001
7.386028e+002	8.750918e+001
7.405313e+002	8.749521e+001
7.424597e+002	8.743456e+001
7.443882e+002	8.736752e+001
7.463167e+002	8.737817e+001
7.482451e+002	8.738592e+001
7.501736e+002	8.735667e+001
7.521021e+002	8.731029e+001
7.540305e+002	8.727136e+001
7.559590e+002	8.722851e+001
7.578875e+002	8.715557e+001
7.598159e+002	8.712158e+001
7.617444e+002	8.712350e+001
7.636729e+002	8.707887e+001
7.656013e+002	8.702335e+001
7.675298e+002	8.696665e+001
7.694583e+002	8.692309e+001
7.713867e+002	8.688066e+001
7.733152e+002	8.681519e+001
7.752437e+002	8.677547e+001
7.771721e+002	8.676086e+001
7.791006e+002	8.672469e+001
7.810291e+002	8.667715e+001
7.829575e+002	8.664322e+001

7.848860e+002	8.660884e+001
7.868145e+002	8.656731e+001
7.887429e+002	8.653214e+001
7.906714e+002	8.651196e+001
7.925999e+002	8.646156e+001
7.945283e+002	8.637872e+001
7.964568e+002	8.634402e+001
7.983853e+002	8.631578e+001
8.003137e+002	8.629401e+001
8.022422e+002	8.629310e+001
8.041707e+002	8.630604e+001
8.060991e+002	8.636111e+001
8.080276e+002	8.642759e+001
8.099561e+002	8.648821e+001
8.118845e+002	8.654510e+001
8.138130e+002	8.659000e+001
8.157415e+002	8.666673e+001
8.176699e+002	8.671778e+001
8.195984e+002	8.672324e+001
8.215269e+002	8.670934e+001
8.234553e+002	8.668421e+001
8.253838e+002	8.666012e+001
8.273123e+002	8.668208e+001
8.292407e+002	8.669954e+001
8.311692e+002	8.672202e+001
8.330977e+002	8.679671e+001
8.350261e+002	8.689443e+001
8.369546e+002	8.693114e+001
8.388831e+002	8.696352e+001
8.408115e+002	8.702180e+001
8.427400e+002	8.702039e+001
8.446685e+002	8.702926e+001
8.465969e+002	8.707336e+001
8.485254e+002	8.704742e+001
8.504539e+002	8.702763e+001
8.523823e+002	8.704369e+001
8.543108e+002	8.703894e+001
8.562393e+002	8.698005e+001
8.581677e+002	8.695126e+001
8.600962e+002	8.691541e+001
8.620247e+002	8.688197e+001
8.639531e+002	8.682124e+001
8.658816e+002	8.676846e+001
8.678101e+002	8.673202e+001
8.697385e+002	8.666866e+001
8.716670e+002	8.660397e+001
8.735955e+002	8.660432e+001
8.755239e+002	8.657180e+001
8.774524e+002	8.653676e+001
8.793809e+002	8.651352e+001

8.813093e+002	8.646035e+001
8.832378e+002	8.642661e+001
8.851663e+002	8.640233e+001
8.870947e+002	8.635596e+001
8.890232e+002	8.634990e+001
8.909517e+002	8.631848e+001
8.928801e+002	8.628027e+001
8.948085e+002	8.625055e+001
8.967370e+002	8.620880e+001
8.986655e+002	8.618355e+001
9.005939e+002	8.615012e+001
9.025224e+002	8.611199e+001
9.044509e+002	8.607145e+001
9.063793e+002	8.604401e+001
9.083078e+002	8.601214e+001
9.102363e+002	8.596175e+001
9.121647e+002	8.595348e+001
9.140932e+002	8.594898e+001
9.160217e+002	8.589482e+001
9.179501e+002	8.584142e+001
9.198786e+002	8.583046e+001
9.218071e+002	8.581075e+001
9.237355e+002	8.577314e+001
9.256640e+002	8.574615e+001
9.275925e+002	8.571980e+001
9.295209e+002	8.567627e+001
9.314494e+002	8.564122e+001
9.333779e+002	8.563527e+001
9.353063e+002	8.562169e+001
9.372348e+002	8.556696e+001
9.391633e+002	8.556241e+001
9.410917e+002	8.554810e+001
9.430202e+002	8.549545e+001
9.449487e+002	8.544624e+001
9.468771e+002	8.540720e+001
9.488056e+002	8.538155e+001
9.507341e+002	8.536569e+001
9.526625e+002	8.532431e+001
9.545910e+002	8.526241e+001
9.565195e+002	8.521664e+001
9.584479e+002	8.519312e+001
9.603764e+002	8.512981e+001
9.623049e+002	8.506854e+001
9.642333e+002	8.502523e+001
9.661618e+002	8.494693e+001
9.680903e+002	8.487111e+001
9.700187e+002	8.482848e+001
9.719472e+002	8.477035e+001
9.738757e+002	8.471013e+001
9.758041e+002	8.465302e+001

9.777326e+002	8.458649e+001
9.796611e+002	8.450665e+001
9.815895e+002	8.441571e+001
9.835180e+002	8.434300e+001
9.854465e+002	8.430724e+001
9.873749e+002	8.424436e+001
9.893034e+002	8.417600e+001
9.912319e+002	8.412225e+001
9.931603e+002	8.406680e+001
9.950888e+002	8.399478e+001
9.970173e+002	8.392442e+001
9.989457e+002	8.384554e+001
1.000874e+003	8.378428e+001
1.002803e+003	8.374148e+001
1.004731e+003	8.371256e+001
1.006660e+003	8.364999e+001
1.008588e+003	8.357677e+001
1.010517e+003	8.350161e+001
1.012445e+003	8.343304e+001
1.014373e+003	8.337148e+001
1.016302e+003	8.329513e+001
1.018230e+003	8.320044e+001
1.020159e+003	8.312006e+001
1.022087e+003	8.304480e+001
1.024016e+003	8.297614e+001
1.025944e+003	8.291988e+001
1.027873e+003	8.284976e+001
1.029801e+003	8.276655e+001
1.031730e+003	8.269288e+001
1.033658e+003	8.263819e+001
1.035587e+003	8.258304e+001
1.037515e+003	8.250539e+001
1.039444e+003	8.244500e+001
1.041372e+003	8.238383e+001
1.043301e+003	8.232950e+001
1.045229e+003	8.226830e+001
1.047157e+003	8.223083e+001
1.049086e+003	8.219236e+001
1.051014e+003	8.212691e+001
1.052943e+003	8.208434e+001
1.054871e+003	8.203242e+001
1.056800e+003	8.195521e+001
1.058728e+003	8.186993e+001
1.060657e+003	8.181854e+001
1.062585e+003	8.179529e+001
1.064514e+003	8.173930e+001
1.066442e+003	8.167568e+001
1.068371e+003	8.161393e+001
1.070299e+003	8.154322e+001
1.072228e+003	8.146093e+001

1.074156e+003	8.138241e+001
1.076084e+003	8.130824e+001
1.078013e+003	8.123111e+001
1.079941e+003	8.116257e+001
1.081870e+003	8.108530e+001
1.083798e+003	8.097511e+001
1.085727e+003	8.087153e+001
1.087655e+003	8.074868e+001
1.089584e+003	8.064713e+001
1.091512e+003	8.053564e+001
1.093441e+003	8.043456e+001
1.095369e+003	8.032619e+001
1.097298e+003	8.020496e+001
1.099226e+003	8.008746e+001
1.101155e+003	7.997780e+001
1.103083e+003	7.984132e+001
1.105011e+003	7.970012e+001
1.106940e+003	7.956817e+001
1.108868e+003	7.943687e+001
1.110797e+003	7.929983e+001
1.112725e+003	7.916660e+001
1.114654e+003	7.902557e+001
1.116582e+003	7.888983e+001
1.118511e+003	7.874467e+001
1.120439e+003	7.859547e+001
1.122368e+003	7.843913e+001
1.124296e+003	7.828685e+001
1.126225e+003	7.811721e+001
1.128153e+003	7.795908e+001
1.130082e+003	7.780476e+001
1.132010e+003	7.765598e+001
1.133938e+003	7.751016e+001
1.135867e+003	7.736694e+001
1.137795e+003	7.721608e+001
1.139724e+003	7.707376e+001
1.141652e+003	7.689684e+001
1.143581e+003	7.675376e+001
1.145509e+003	7.664122e+001
1.147438e+003	7.651636e+001
1.149366e+003	7.637540e+001
1.151295e+003	7.624456e+001
1.153223e+003	7.612154e+001
1.155152e+003	7.601002e+001
1.157080e+003	7.590091e+001
1.159009e+003	7.579748e+001
1.160937e+003	7.570422e+001
1.162865e+003	7.561475e+001
1.164794e+003	7.553299e+001
1.166722e+003	7.546349e+001
1.168651e+003	7.539703e+001

1.170579e+003	7.531571e+001
1.172508e+003	7.523238e+001
1.174436e+003	7.516843e+001
1.176365e+003	7.511725e+001
1.178293e+003	7.506509e+001
1.180222e+003	7.501991e+001
1.182150e+003	7.496439e+001
1.184079e+003	7.490504e+001
1.186007e+003	7.484711e+001
1.187936e+003	7.481057e+001
1.189864e+003	7.478098e+001
1.191792e+003	7.472449e+001
1.193721e+003	7.467647e+001
1.195649e+003	7.462948e+001
1.197578e+003	7.456116e+001
1.199506e+003	7.448711e+001
1.201435e+003	7.441853e+001
1.203363e+003	7.436586e+001
1.205292e+003	7.430306e+001
1.207220e+003	7.422811e+001
1.209149e+003	7.415431e+001
1.211077e+003	7.408751e+001
1.213006e+003	7.404425e+001
1.214934e+003	7.398823e+001
1.216863e+003	7.391413e+001
1.218791e+003	7.383006e+001
1.220719e+003	7.376090e+001
1.222648e+003	7.368729e+001
1.224576e+003	7.361093e+001
1.226505e+003	7.353747e+001
1.228433e+003	7.346243e+001
1.230362e+003	7.339568e+001
1.232290e+003	7.332032e+001
1.234219e+003	7.323136e+001
1.236147e+003	7.313758e+001
1.238076e+003	7.305209e+001
1.240004e+003	7.298253e+001
1.241933e+003	7.289542e+001
1.243861e+003	7.282153e+001
1.245790e+003	7.277407e+001
1.247718e+003	7.271955e+001
1.249646e+003	7.263190e+001
1.251575e+003	7.254531e+001
1.253503e+003	7.247585e+001
1.255432e+003	7.240965e+001
1.257360e+003	7.234756e+001
1.259289e+003	7.229385e+001
1.261217e+003	7.225890e+001
1.263146e+003	7.221973e+001
1.265074e+003	7.216647e+001

1.267003e+003	7.212884e+001
1.268931e+003	7.209155e+001
1.270860e+003	7.206772e+001
1.272788e+003	7.207320e+001
1.274717e+003	7.209284e+001
1.276645e+003	7.211948e+001
1.278573e+003	7.213491e+001
1.280502e+003	7.215687e+001
1.282430e+003	7.220512e+001
1.284359e+003	7.225749e+001
1.286287e+003	7.230196e+001
1.288216e+003	7.237926e+001
1.290144e+003	7.248209e+001
1.292073e+003	7.257773e+001
1.294001e+003	7.267123e+001
1.295930e+003	7.278336e+001
1.297858e+003	7.291162e+001
1.299787e+003	7.305650e+001
1.301715e+003	7.318968e+001
1.303644e+003	7.331754e+001
1.305572e+003	7.343507e+001
1.307500e+003	7.358399e+001
1.309429e+003	7.373743e+001
1.311357e+003	7.386746e+001
1.313286e+003	7.401661e+001
1.315214e+003	7.417223e+001
1.317143e+003	7.429677e+001
1.319071e+003	7.443166e+001
1.321000e+003	7.457484e+001
1.322928e+003	7.470790e+001
1.324857e+003	7.483572e+001
1.326785e+003	7.495186e+001
1.328714e+003	7.505438e+001
1.330642e+003	7.516064e+001
1.332571e+003	7.525498e+001
1.334499e+003	7.533686e+001
1.336427e+003	7.539899e+001
1.338356e+003	7.546255e+001
1.340284e+003	7.562325e+001
1.342213e+003	7.575050e+001
1.344141e+003	7.582214e+001
1.346070e+003	7.590878e+001
1.347998e+003	7.599232e+001
1.349927e+003	7.609051e+001
1.351855e+003	7.615536e+001
1.353784e+003	7.621605e+001
1.355712e+003	7.631741e+001
1.357641e+003	7.640584e+001
1.359569e+003	7.645937e+001
1.361498e+003	7.651556e+001

1.363426e+003	7.665001e+001
1.365354e+003	7.675118e+001
1.367283e+003	7.681898e+001
1.369211e+003	7.690647e+001
1.371140e+003	7.698483e+001
1.373068e+003	7.704646e+001
1.374997e+003	7.718884e+001
1.376925e+003	7.729501e+001
1.378854e+003	7.739106e+001
1.380782e+003	7.749557e+001
1.382711e+003	7.758270e+001
1.384639e+003	7.765336e+001
1.386568e+003	7.775244e+001
1.388496e+003	7.789532e+001
1.390424e+003	7.797633e+001
1.392353e+003	7.804871e+001
1.394281e+003	7.811432e+001
1.396210e+003	7.819989e+001
1.398138e+003	7.827142e+001
1.400067e+003	7.842270e+001
1.401995e+003	7.849474e+001
1.403924e+003	7.855222e+001
1.405852e+003	7.866962e+001
1.407781e+003	7.874253e+001
1.409709e+003	7.877237e+001
1.411638e+003	7.884945e+001
1.413566e+003	7.894526e+001
1.415495e+003	7.896509e+001
1.417423e+003	7.898650e+001
1.419351e+003	7.916100e+001
1.421280e+003	7.921481e+001
1.423208e+003	7.921334e+001
1.425137e+003	7.928024e+001
1.427065e+003	7.930399e+001
1.428994e+003	7.932249e+001
1.430922e+003	7.934689e+001
1.432851e+003	7.935474e+001
1.434779e+003	7.936456e+001
1.436708e+003	7.944285e+001
1.438636e+003	7.945748e+001
1.440565e+003	7.942678e+001
1.442493e+003	7.940238e+001
1.444422e+003	7.937746e+001
1.446350e+003	7.934589e+001
1.448278e+003	7.931617e+001
1.450207e+003	7.927309e+001
1.452135e+003	7.920870e+001
1.454064e+003	7.914615e+001
1.455992e+003	7.911343e+001
1.457921e+003	7.911571e+001

1.459849e+003	7.911102e+001
1.461778e+003	7.907259e+001
1.463706e+003	7.902364e+001
1.465635e+003	7.899270e+001
1.467563e+003	7.893545e+001
1.469492e+003	7.886468e+001
1.471420e+003	7.877908e+001
1.473349e+003	7.872099e+001
1.475277e+003	7.871488e+001
1.477205e+003	7.872662e+001
1.479134e+003	7.868021e+001
1.481062e+003	7.861758e+001
1.482991e+003	7.859381e+001
1.484919e+003	7.854726e+001
1.486848e+003	7.845824e+001
1.488776e+003	7.838006e+001
1.490705e+003	7.844147e+001
1.492633e+003	7.846690e+001
1.494562e+003	7.844420e+001
1.496490e+003	7.840675e+001
1.498419e+003	7.836274e+001
1.500347e+003	7.831841e+001
1.502276e+003	7.829225e+001
1.504204e+003	7.830886e+001
1.506132e+003	7.819380e+001
1.508061e+003	7.817702e+001
1.509989e+003	7.814874e+001
1.511918e+003	7.815932e+001
1.513846e+003	7.809435e+001
1.515775e+003	7.799522e+001
1.517703e+003	7.803480e+001
1.519632e+003	7.802482e+001
1.521560e+003	7.791142e+001
1.523489e+003	7.786880e+001
1.525417e+003	7.781653e+001
1.527346e+003	7.781289e+001
1.529274e+003	7.780911e+001
1.531203e+003	7.776363e+001
1.533131e+003	7.770805e+001
1.535059e+003	7.766044e+001
1.536988e+003	7.759380e+001
1.538916e+003	7.748688e+001
1.540845e+003	7.733464e+001
1.542773e+003	7.735632e+001
1.544702e+003	7.735878e+001
1.546630e+003	7.741140e+001
1.548559e+003	7.736726e+001
1.550487e+003	7.735736e+001
1.552416e+003	7.733225e+001
1.554344e+003	7.728202e+001

1.556273e+003	7.724066e+001
1.558201e+003	7.733473e+001
1.560130e+003	7.758769e+001
1.562058e+003	7.742377e+001
1.563986e+003	7.737483e+001
1.565915e+003	7.741058e+001
1.567843e+003	7.742756e+001
1.569772e+003	7.756487e+001
1.571700e+003	7.760895e+001
1.573629e+003	7.763779e+001
1.575557e+003	7.775460e+001
1.577486e+003	7.794193e+001
1.579414e+003	7.801112e+001
1.581343e+003	7.815046e+001
1.583271e+003	7.832436e+001
1.585200e+003	7.853390e+001
1.587128e+003	7.870414e+001
1.589057e+003	7.892088e+001
1.590985e+003	7.913315e+001
1.592913e+003	7.939081e+001
1.594842e+003	7.963559e+001
1.596770e+003	7.991177e+001
1.598699e+003	8.013456e+001
1.600627e+003	8.038575e+001
1.602556e+003	8.063142e+001
1.604484e+003	8.092570e+001
1.606413e+003	8.114211e+001
1.608341e+003	8.144165e+001
1.610270e+003	8.173528e+001
1.612198e+003	8.201382e+001
1.614127e+003	8.220467e+001
1.616055e+003	8.250483e+001
1.617984e+003	8.284127e+001
1.619912e+003	8.304162e+001
1.621840e+003	8.323136e+001
1.623769e+003	8.364919e+001
1.625697e+003	8.390791e+001
1.627626e+003	8.418134e+001
1.629554e+003	8.448698e+001
1.631483e+003	8.476538e+001
1.633411e+003	8.497922e+001
1.635340e+003	8.529893e+001
1.637268e+003	8.563461e+001
1.639197e+003	8.593915e+001
1.641125e+003	8.621964e+001
1.643054e+003	8.651537e+001
1.644982e+003	8.673062e+001
1.646911e+003	8.700734e+001
1.648839e+003	8.728951e+001
1.650767e+003	8.763773e+001

1.652696e+003	8.788246e+001
1.654624e+003	8.826460e+001
1.656553e+003	8.835141e+001
1.658481e+003	8.859013e+001
1.660410e+003	8.874861e+001
1.662338e+003	8.893456e+001
1.664267e+003	8.908594e+001
1.666195e+003	8.923731e+001
1.668124e+003	8.930270e+001
1.670052e+003	8.944666e+001
1.671981e+003	8.955087e+001
1.673909e+003	8.972110e+001
1.675838e+003	8.980761e+001
1.677766e+003	8.984846e+001
1.679694e+003	8.981858e+001
1.681623e+003	8.987070e+001
1.683551e+003	8.987336e+001
1.685480e+003	9.002200e+001
1.687408e+003	8.996072e+001
1.689337e+003	8.999266e+001
1.691265e+003	9.001228e+001
1.693194e+003	9.009798e+001
1.695122e+003	9.007829e+001
1.697051e+003	8.997868e+001
1.698979e+003	9.010902e+001
1.700908e+003	9.022781e+001
1.702836e+003	8.999780e+001
1.704765e+003	8.995532e+001
1.706693e+003	8.990058e+001
1.708621e+003	8.991257e+001
1.710550e+003	8.984124e+001
1.712478e+003	8.982158e+001
1.714407e+003	8.968900e+001
1.716335e+003	8.967433e+001
1.718264e+003	8.974352e+001
1.720192e+003	8.976933e+001
1.722121e+003	8.965594e+001
1.724049e+003	8.965206e+001
1.725978e+003	8.967248e+001
1.727906e+003	8.964255e+001
1.729835e+003	8.950099e+001
1.731763e+003	8.955621e+001
1.733692e+003	8.955749e+001
1.735620e+003	8.946535e+001
1.737548e+003	8.933726e+001
1.739477e+003	8.930782e+001
1.741405e+003	8.928761e+001
1.743334e+003	8.925069e+001
1.745262e+003	8.912301e+001
1.747191e+003	8.906645e+001

1.749119e+003	8.897516e+001
1.751048e+003	8.906275e+001
1.752976e+003	8.905530e+001
1.754905e+003	8.907235e+001
1.756833e+003	8.897320e+001
1.758762e+003	8.893499e+001
1.760690e+003	8.892548e+001
1.762619e+003	8.888987e+001
1.764547e+003	8.882091e+001
1.766475e+003	8.882131e+001
1.768404e+003	8.872973e+001
1.770332e+003	8.865611e+001
1.772261e+003	8.856567e+001
1.774189e+003	8.859827e+001
1.776118e+003	8.856901e+001
1.778046e+003	8.855643e+001
1.779975e+003	8.847244e+001
1.781903e+003	8.839029e+001
1.783832e+003	8.834813e+001
1.785760e+003	8.836555e+001
1.787689e+003	8.832777e+001
1.789617e+003	8.827263e+001
1.791546e+003	8.820370e+001
1.793474e+003	8.819193e+001
1.795402e+003	8.812728e+001
1.797331e+003	8.815150e+001
1.799259e+003	8.811272e+001
1.801188e+003	8.801784e+001
1.803116e+003	8.798489e+001
1.805045e+003	8.803235e+001
1.806973e+003	8.802290e+001
1.808902e+003	8.803088e+001
1.810830e+003	8.795203e+001
1.812759e+003	8.790909e+001
1.814687e+003	8.784955e+001
1.816616e+003	8.782680e+001
1.818544e+003	8.780517e+001
1.820473e+003	8.779030e+001
1.822401e+003	8.772830e+001
1.824329e+003	8.769595e+001
1.826258e+003	8.763521e+001
1.828186e+003	8.761253e+001
1.830115e+003	8.754374e+001
1.832043e+003	8.754787e+001
1.833972e+003	8.749409e+001
1.835900e+003	8.747031e+001
1.837829e+003	8.742320e+001
1.839757e+003	8.742394e+001
1.841686e+003	8.736342e+001
1.843614e+003	8.733315e+001

1.845543e+003	8.729356e+001
1.847471e+003	8.732059e+001
1.849400e+003	8.735654e+001
1.851328e+003	8.738641e+001
1.853256e+003	8.731682e+001
1.855185e+003	8.728906e+001
1.857113e+003	8.722353e+001
1.859042e+003	8.714819e+001
1.860970e+003	8.711328e+001
1.862899e+003	8.712415e+001
1.864827e+003	8.700512e+001
1.866756e+003	8.701378e+001
1.868684e+003	8.688397e+001
1.870613e+003	8.686134e+001
1.872541e+003	8.698617e+001
1.874470e+003	8.700355e+001
1.876398e+003	8.684846e+001
1.878327e+003	8.680996e+001
1.880255e+003	8.670746e+001
1.882183e+003	8.672509e+001
1.884112e+003	8.671732e+001
1.886040e+003	8.664630e+001
1.887969e+003	8.652822e+001
1.889897e+003	8.656796e+001
1.891826e+003	8.658463e+001
1.893754e+003	8.661829e+001
1.895683e+003	8.666909e+001
1.897611e+003	8.663069e+001
1.899540e+003	8.667808e+001
1.901468e+003	8.672416e+001
1.903397e+003	8.653503e+001
1.905325e+003	8.636002e+001
1.907254e+003	8.632231e+001
1.909182e+003	8.628477e+001
1.911110e+003	8.623213e+001
1.913039e+003	8.630156e+001
1.914967e+003	8.625580e+001
1.916896e+003	8.621888e+001
1.918824e+003	8.622841e+001
1.920753e+003	8.628361e+001
1.922681e+003	8.632642e+001
1.924610e+003	8.638177e+001
1.926538e+003	8.616480e+001
1.928467e+003	8.612940e+001
1.930395e+003	8.617017e+001
1.932324e+003	8.617941e+001
1.934252e+003	8.609229e+001
1.936181e+003	8.617890e+001
1.938109e+003	8.628394e+001
1.940037e+003	8.626614e+001

1.941966e+003	8.634310e+001
1.943894e+003	8.639495e+001
1.945823e+003	8.630650e+001
1.947751e+003	8.640609e+001
1.949680e+003	8.625375e+001
1.951608e+003	8.613464e+001
1.953537e+003	8.618980e+001
1.955465e+003	8.635251e+001
1.957394e+003	8.598695e+001
1.959322e+003	8.577515e+001
1.961251e+003	8.609396e+001
1.963179e+003	8.615548e+001
1.965108e+003	8.617946e+001
1.967036e+003	8.673009e+001
1.968964e+003	8.685182e+001
1.970893e+003	8.693045e+001
1.972821e+003	8.687054e+001
1.974750e+003	8.661114e+001
1.976678e+003	8.631477e+001
1.978607e+003	8.654947e+001
1.980535e+003	8.654090e+001
1.982464e+003	8.623057e+001
1.984392e+003	8.604604e+001
1.986321e+003	8.604323e+001
1.988249e+003	8.608733e+001
1.990178e+003	8.586079e+001
1.992106e+003	8.559507e+001
1.994035e+003	8.609916e+001
1.995963e+003	8.596204e+001
1.997891e+003	8.553187e+001
1.999820e+003	8.549600e+001
2.001748e+003	8.568690e+001
2.003677e+003	8.572100e+001
2.005605e+003	8.596316e+001
2.007534e+003	8.593169e+001
2.009462e+003	8.580601e+001
2.011391e+003	8.580250e+001
2.013319e+003	8.604013e+001
2.015248e+003	8.616226e+001
2.017176e+003	8.630936e+001
2.019105e+003	8.581786e+001
2.021033e+003	8.547755e+001
2.022962e+003	8.541614e+001
2.024890e+003	8.535566e+001
2.026818e+003	8.512548e+001
2.028747e+003	8.528190e+001
2.030675e+003	8.523862e+001
2.032604e+003	8.541099e+001
2.034532e+003	8.570799e+001
2.036461e+003	8.578254e+001

2.038389e+003	8.537186e+001
2.040318e+003	8.549038e+001
2.042246e+003	8.559682e+001
2.044175e+003	8.573296e+001
2.046103e+003	8.524586e+001
2.048032e+003	8.489983e+001
2.049960e+003	8.486473e+001
2.051889e+003	8.499909e+001
2.053817e+003	8.493212e+001
2.055746e+003	8.488800e+001
2.057674e+003	8.494127e+001
2.059603e+003	8.483298e+001
2.061531e+003	8.427592e+001
2.063459e+003	8.431133e+001
2.065388e+003	8.464182e+001
2.067316e+003	8.484668e+001
2.069245e+003	8.484606e+001
2.071173e+003	8.468456e+001
2.073102e+003	8.444530e+001
2.075030e+003	8.431969e+001
2.076959e+003	8.425919e+001
2.078887e+003	8.424705e+001
2.080816e+003	8.402236e+001
2.082744e+003	8.401784e+001
2.084673e+003	8.408098e+001
2.086601e+003	8.415024e+001
2.088530e+003	8.398634e+001
2.090458e+003	8.383835e+001
2.092386e+003	8.386967e+001
2.094315e+003	8.395134e+001
2.096243e+003	8.382744e+001
2.098172e+003	8.376046e+001
2.100100e+003	8.381686e+001
2.102029e+003	8.370964e+001
2.103957e+003	8.365588e+001
2.105886e+003	8.382208e+001
2.107814e+003	8.373560e+001
2.109743e+003	8.345891e+001
2.111671e+003	8.335828e+001
2.113600e+003	8.338371e+001
2.115528e+003	8.343667e+001
2.117457e+003	8.350880e+001
2.119385e+003	8.361058e+001
2.121313e+003	8.373354e+001
2.123242e+003	8.369253e+001
2.125170e+003	8.361723e+001
2.127099e+003	8.340511e+001
2.129027e+003	8.348828e+001
2.130956e+003	8.371286e+001
2.132884e+003	8.401640e+001

2.134813e+003	8.404799e+001
2.136741e+003	8.394029e+001
2.138670e+003	8.380544e+001
2.140598e+003	8.388599e+001
2.142527e+003	8.367483e+001
2.144455e+003	8.359531e+001
2.146384e+003	8.354897e+001
2.148312e+003	8.387714e+001
2.150240e+003	8.437738e+001
2.152169e+003	8.431685e+001
2.154097e+003	8.385161e+001
2.156026e+003	8.444631e+001
2.157954e+003	8.486270e+001
2.159883e+003	8.449098e+001
2.161811e+003	8.427789e+001
2.163740e+003	8.399774e+001
2.165668e+003	8.366539e+001
2.167597e+003	8.335915e+001
2.169525e+003	8.359818e+001
2.171454e+003	8.364844e+001
2.173382e+003	8.377370e+001
2.175311e+003	8.379514e+001
2.177239e+003	8.361521e+001
2.179167e+003	8.402800e+001
2.181096e+003	8.380898e+001
2.183024e+003	8.359200e+001
2.184953e+003	8.314555e+001
2.186881e+003	8.287186e+001
2.188810e+003	8.301106e+001
2.190738e+003	8.292171e+001
2.192667e+003	8.308049e+001
2.194595e+003	8.346247e+001
2.196524e+003	8.338564e+001
2.198452e+003	8.314626e+001
2.200381e+003	8.294431e+001
2.202309e+003	8.286971e+001
2.204238e+003	8.264737e+001
2.206166e+003	8.271567e+001
2.208094e+003	8.291793e+001
2.210023e+003	8.318884e+001
2.211951e+003	8.308672e+001
2.213880e+003	8.282028e+001
2.215808e+003	8.256062e+001
2.217737e+003	8.237344e+001
2.219665e+003	8.232927e+001
2.221594e+003	8.273248e+001
2.223522e+003	8.267085e+001
2.225451e+003	8.259343e+001
2.227379e+003	8.253964e+001
2.229308e+003	8.243685e+001

2.231236e+003	8.246270e+001
2.233165e+003	8.252221e+001
2.235093e+003	8.234632e+001
2.237021e+003	8.224654e+001
2.238950e+003	8.228373e+001
2.240878e+003	8.257925e+001
2.242807e+003	8.243797e+001
2.244735e+003	8.203918e+001
2.246664e+003	8.216289e+001
2.248592e+003	8.249148e+001
2.250521e+003	8.235905e+001
2.252449e+003	8.215819e+001
2.254378e+003	8.203667e+001
2.256306e+003	8.202381e+001
2.258235e+003	8.197528e+001
2.260163e+003	8.190034e+001
2.262092e+003	8.176546e+001
2.264020e+003	8.186975e+001
2.265948e+003	8.183558e+001
2.267877e+003	8.179941e+001
2.269805e+003	8.208150e+001
2.271734e+003	8.202217e+001
2.273662e+003	8.176208e+001
2.275591e+003	8.164780e+001
2.277519e+003	8.166171e+001
2.279448e+003	8.174348e+001
2.281376e+003	8.162762e+001
2.283305e+003	8.146663e+001
2.285233e+003	8.131717e+001
2.287162e+003	8.123758e+001
2.289090e+003	8.124194e+001
2.291019e+003	8.128535e+001
2.292947e+003	8.124497e+001
2.294875e+003	8.122347e+001
2.296804e+003	8.118174e+001
2.298732e+003	8.118369e+001
2.300661e+003	8.118978e+001
2.302589e+003	8.117261e+001
2.304518e+003	8.107648e+001
2.306446e+003	8.106823e+001
2.308375e+003	8.106002e+001
2.310303e+003	8.107551e+001
2.312232e+003	8.101725e+001
2.314160e+003	8.095851e+001
2.316089e+003	8.090516e+001
2.318017e+003	8.092409e+001
2.319946e+003	8.086907e+001
2.321874e+003	8.077866e+001
2.323802e+003	8.063297e+001
2.325731e+003	8.061282e+001

2.327659e+003	8.053781e+001
2.329588e+003	8.040277e+001
2.331516e+003	8.046085e+001
2.333445e+003	8.064075e+001
2.335373e+003	8.065504e+001
2.337302e+003	8.066414e+001
2.339230e+003	8.054138e+001
2.341159e+003	8.050966e+001
2.343087e+003	8.046465e+001
2.345016e+003	8.052489e+001
2.346944e+003	8.043221e+001
2.348873e+003	8.045053e+001
2.350801e+003	8.041955e+001
2.352729e+003	8.040930e+001
2.354658e+003	8.060618e+001
2.356586e+003	8.045902e+001
2.358515e+003	8.030520e+001
2.360443e+003	8.029868e+001
2.362372e+003	8.011990e+001
2.364300e+003	8.042101e+001
2.366229e+003	8.030659e+001
2.368157e+003	8.001153e+001
2.370086e+003	8.003598e+001
2.372014e+003	8.011764e+001
2.373942e+003	8.005939e+001
2.375871e+003	8.008077e+001
2.377799e+003	8.010747e+001
2.379728e+003	8.008760e+001
2.381656e+003	8.006891e+001
2.383585e+003	7.997377e+001
2.385513e+003	7.989465e+001
2.387442e+003	7.990507e+001
2.389370e+003	7.990489e+001
2.391299e+003	7.986906e+001
2.393227e+003	7.982689e+001
2.395156e+003	7.981143e+001
2.397084e+003	7.980357e+001
2.399012e+003	7.974393e+001
2.400941e+003	7.977151e+001
2.402869e+003	7.981072e+001
2.404798e+003	7.982203e+001
2.406726e+003	7.974836e+001
2.408655e+003	7.965956e+001
2.410583e+003	7.959359e+001
2.412512e+003	7.969617e+001
2.414440e+003	7.975340e+001
2.416369e+003	7.963009e+001
2.418297e+003	7.956776e+001
2.420226e+003	7.953638e+001
2.422154e+003	7.958540e+001

2.424083e+003	7.967828e+001
2.426011e+003	7.965067e+001
2.427939e+003	7.956590e+001
2.429868e+003	7.955756e+001
2.431796e+003	7.944588e+001
2.433725e+003	7.941955e+001
2.435653e+003	7.946718e+001
2.437582e+003	7.944286e+001
2.439510e+003	7.934731e+001
2.441439e+003	7.932047e+001
2.443367e+003	7.930858e+001
2.445296e+003	7.924660e+001
2.447224e+003	7.923182e+001
2.449153e+003	7.921545e+001
2.451081e+003	7.918368e+001
2.453010e+003	7.914982e+001
2.454938e+003	7.910524e+001
2.456866e+003	7.907346e+001
2.458795e+003	7.914859e+001
2.460723e+003	7.917263e+001
2.462652e+003	7.912362e+001
2.464580e+003	7.911026e+001
2.466509e+003	7.912650e+001
2.468437e+003	7.908712e+001
2.470366e+003	7.903126e+001
2.472294e+003	7.896461e+001
2.474223e+003	7.889948e+001
2.476151e+003	7.893184e+001
2.478080e+003	7.898983e+001
2.480008e+003	7.898920e+001
2.481937e+003	7.894433e+001
2.483865e+003	7.884724e+001
2.485793e+003	7.877485e+001
2.487722e+003	7.884185e+001
2.489650e+003	7.885757e+001
2.491579e+003	7.875449e+001
2.493507e+003	7.876273e+001
2.495436e+003	7.877502e+001
2.497364e+003	7.877879e+001
2.499293e+003	7.874158e+001
2.501221e+003	7.867072e+001
2.503150e+003	7.867373e+001
2.505078e+003	7.868309e+001
2.507007e+003	7.860529e+001
2.508935e+003	7.855350e+001
2.510864e+003	7.852287e+001
2.512792e+003	7.846121e+001
2.514720e+003	7.849691e+001
2.516649e+003	7.851149e+001
2.518577e+003	7.852895e+001

2.520506e+003	7.852534e+001
2.522434e+003	7.843345e+001
2.524363e+003	7.839720e+001
2.526291e+003	7.838757e+001
2.528220e+003	7.838850e+001
2.530148e+003	7.837092e+001
2.532077e+003	7.833383e+001
2.534005e+003	7.825312e+001
2.535934e+003	7.821422e+001
2.537862e+003	7.825953e+001
2.539791e+003	7.827516e+001
2.541719e+003	7.823646e+001
2.543647e+003	7.816210e+001
2.545576e+003	7.817799e+001
2.547504e+003	7.820174e+001
2.549433e+003	7.815253e+001
2.551361e+003	7.809176e+001
2.553290e+003	7.809560e+001
2.555218e+003	7.806802e+001
2.557147e+003	7.797858e+001
2.559075e+003	7.794035e+001
2.561004e+003	7.790214e+001
2.562932e+003	7.788761e+001
2.564861e+003	7.785371e+001
2.566789e+003	7.786054e+001
2.568718e+003	7.790021e+001
2.570646e+003	7.786666e+001
2.572574e+003	7.784756e+001
2.574503e+003	7.780664e+001
2.576431e+003	7.780705e+001
2.578360e+003	7.785013e+001
2.580288e+003	7.778392e+001
2.582217e+003	7.768997e+001
2.584145e+003	7.767781e+001
2.586074e+003	7.769226e+001
2.588002e+003	7.765181e+001
2.589931e+003	7.750682e+001
2.591859e+003	7.743498e+001
2.593788e+003	7.748690e+001
2.595716e+003	7.746881e+001
2.597645e+003	7.740093e+001
2.599573e+003	7.735394e+001
2.601501e+003	7.736472e+001
2.603430e+003	7.737418e+001
2.605358e+003	7.728677e+001
2.607287e+003	7.725196e+001
2.609215e+003	7.721713e+001
2.611144e+003	7.714839e+001
2.613072e+003	7.711067e+001
2.615001e+003	7.708614e+001

2.616929e+003	7.709985e+001
2.618858e+003	7.710303e+001
2.620786e+003	7.701371e+001
2.622715e+003	7.696413e+001
2.624643e+003	7.695137e+001
2.626572e+003	7.693189e+001
2.628500e+003	7.688570e+001
2.630428e+003	7.683890e+001
2.632357e+003	7.677306e+001
2.634285e+003	7.671356e+001
2.636214e+003	7.668073e+001
2.638142e+003	7.659013e+001
2.640071e+003	7.657731e+001
2.641999e+003	7.658387e+001
2.643928e+003	7.651361e+001
2.645856e+003	7.646284e+001
2.647785e+003	7.640961e+001
2.649713e+003	7.634387e+001
2.651642e+003	7.631898e+001
2.653570e+003	7.633749e+001
2.655499e+003	7.629937e+001
2.657427e+003	7.620327e+001
2.659355e+003	7.615211e+001
2.661284e+003	7.614870e+001
2.663212e+003	7.611047e+001
2.665141e+003	7.608067e+001
2.667069e+003	7.609171e+001
2.668998e+003	7.604623e+001
2.670926e+003	7.597134e+001
2.672855e+003	7.594989e+001
2.674783e+003	7.594254e+001
2.676712e+003	7.591948e+001
2.678640e+003	7.588293e+001
2.680569e+003	7.587509e+001
2.682497e+003	7.591187e+001
2.684426e+003	7.591505e+001
2.686354e+003	7.587186e+001
2.688282e+003	7.583479e+001
2.690211e+003	7.580775e+001
2.692139e+003	7.575669e+001
2.694068e+003	7.576343e+001
2.695996e+003	7.578470e+001
2.697925e+003	7.574862e+001
2.699853e+003	7.570522e+001
2.701782e+003	7.568461e+001
2.703710e+003	7.565338e+001
2.705639e+003	7.561216e+001
2.707567e+003	7.558649e+001
2.709496e+003	7.556386e+001
2.711424e+003	7.555983e+001

2.713353e+003	7.556081e+001
2.715281e+003	7.554273e+001
2.717209e+003	7.553307e+001
2.719138e+003	7.550013e+001
2.721066e+003	7.542958e+001
2.722995e+003	7.538012e+001
2.724923e+003	7.535629e+001
2.726852e+003	7.535077e+001
2.728780e+003	7.535368e+001
2.730709e+003	7.536125e+001
2.732637e+003	7.534550e+001
2.734566e+003	7.529934e+001
2.736494e+003	7.527763e+001
2.738423e+003	7.526220e+001
2.740351e+003	7.524146e+001
2.742280e+003	7.520420e+001
2.744208e+003	7.519476e+001
2.746136e+003	7.520599e+001
2.748065e+003	7.520072e+001
2.749993e+003	7.515914e+001
2.751922e+003	7.511089e+001
2.753850e+003	7.508324e+001
2.755779e+003	7.506728e+001
2.757707e+003	7.503379e+001
2.759636e+003	7.500478e+001
2.761564e+003	7.501554e+001
2.763493e+003	7.501778e+001
2.765421e+003	7.498475e+001
2.767350e+003	7.492972e+001
2.769278e+003	7.488432e+001
2.771207e+003	7.486338e+001
2.773135e+003	7.486852e+001
2.775063e+003	7.483475e+001
2.776992e+003	7.480202e+001
2.778920e+003	7.478339e+001
2.780849e+003	7.475896e+001
2.782777e+003	7.475698e+001
2.784706e+003	7.475518e+001
2.786634e+003	7.473370e+001
2.788563e+003	7.477834e+001
2.790491e+003	7.475930e+001
2.792420e+003	7.469086e+001
2.794348e+003	7.466529e+001
2.796277e+003	7.461119e+001
2.798205e+003	7.456665e+001
2.800134e+003	7.456501e+001
2.802062e+003	7.454888e+001
2.803990e+003	7.453517e+001
2.805919e+003	7.453571e+001
2.807847e+003	7.451969e+001

2.809776e+003	7.449014e+001
2.811704e+003	7.447348e+001
2.813633e+003	7.448347e+001
2.815561e+003	7.448676e+001
2.817490e+003	7.443646e+001
2.819418e+003	7.436203e+001
2.821347e+003	7.431172e+001
2.823275e+003	7.431374e+001
2.825204e+003	7.433739e+001
2.827132e+003	7.436347e+001
2.829061e+003	7.435426e+001
2.830989e+003	7.430611e+001
2.832917e+003	7.429171e+001
2.834846e+003	7.424638e+001
2.836774e+003	7.420393e+001
2.838703e+003	7.416584e+001
2.840631e+003	7.410994e+001
2.842560e+003	7.409856e+001
2.844488e+003	7.411737e+001
2.846417e+003	7.412148e+001
2.848345e+003	7.412213e+001
2.850274e+003	7.409625e+001
2.852202e+003	7.403139e+001
2.854131e+003	7.396444e+001
2.856059e+003	7.397279e+001
2.857988e+003	7.401202e+001
2.859916e+003	7.399437e+001
2.861844e+003	7.396957e+001
2.863773e+003	7.393231e+001
2.865701e+003	7.392197e+001
2.867630e+003	7.392313e+001
2.869558e+003	7.388351e+001
2.871487e+003	7.385637e+001
2.873415e+003	7.386586e+001
2.875344e+003	7.384829e+001
2.877272e+003	7.383202e+001
2.879201e+003	7.382237e+001
2.881129e+003	7.377515e+001
2.883058e+003	7.375361e+001
2.884986e+003	7.375530e+001
2.886915e+003	7.372889e+001
2.888843e+003	7.371864e+001
2.890771e+003	7.369349e+001
2.892700e+003	7.368544e+001
2.894628e+003	7.364143e+001
2.896557e+003	7.358073e+001
2.898485e+003	7.356273e+001
2.900414e+003	7.357233e+001
2.902342e+003	7.356403e+001
2.904271e+003	7.352520e+001

2.906199e+003	7.355527e+001
2.908128e+003	7.355287e+001
2.910056e+003	7.350698e+001
2.911985e+003	7.345455e+001
2.913913e+003	7.342355e+001
2.915842e+003	7.346188e+001
2.917770e+003	7.346577e+001
2.919698e+003	7.349243e+001
2.921627e+003	7.349467e+001
2.923555e+003	7.343204e+001
2.925484e+003	7.337875e+001
2.927412e+003	7.334785e+001
2.929341e+003	7.327699e+001
2.931269e+003	7.325356e+001
2.933198e+003	7.328218e+001
2.935126e+003	7.329820e+001
2.937055e+003	7.328693e+001
2.938983e+003	7.328690e+001
2.940912e+003	7.326642e+001
2.942840e+003	7.325058e+001
2.944769e+003	7.320486e+001
2.946697e+003	7.318821e+001
2.948625e+003	7.318597e+001
2.950554e+003	7.316303e+001
2.952482e+003	7.312725e+001
2.954411e+003	7.309543e+001
2.956339e+003	7.311089e+001
2.958268e+003	7.312350e+001
2.960196e+003	7.310236e+001
2.962125e+003	7.308881e+001
2.964053e+003	7.311900e+001
2.965982e+003	7.308384e+001
2.967910e+003	7.304969e+001
2.969839e+003	7.302971e+001
2.971767e+003	7.303338e+001
2.973696e+003	7.301105e+001
2.975624e+003	7.294013e+001
2.977552e+003	7.294261e+001
2.979481e+003	7.291550e+001
2.981409e+003	7.286972e+001
2.983338e+003	7.286109e+001
2.985266e+003	7.288451e+001
2.987195e+003	7.291102e+001
2.989123e+003	7.287087e+001
2.991052e+003	7.277525e+001
2.992980e+003	7.276482e+001
2.994909e+003	7.276997e+001
2.996837e+003	7.275882e+001
2.998766e+003	7.275439e+001
3.000694e+003	7.273715e+001

3.002623e+003	7.268286e+001
3.004551e+003	7.266436e+001
3.006479e+003	7.263803e+001
3.008408e+003	7.258923e+001
3.010336e+003	7.254800e+001
3.012265e+003	7.251340e+001
3.014193e+003	7.249789e+001
3.016122e+003	7.251559e+001
3.018050e+003	7.252063e+001
3.019979e+003	7.251102e+001
3.021907e+003	7.251018e+001
3.023836e+003	7.244093e+001
3.025764e+003	7.237171e+001
3.027693e+003	7.236432e+001
3.029621e+003	7.232944e+001
3.031550e+003	7.229834e+001
3.033478e+003	7.229485e+001
3.035406e+003	7.227264e+001
3.037335e+003	7.228493e+001
3.039263e+003	7.229892e+001
3.041192e+003	7.223608e+001
3.043120e+003	7.217236e+001
3.045049e+003	7.223627e+001
3.046977e+003	7.224953e+001
3.048906e+003	7.216824e+001
3.050834e+003	7.208830e+001
3.052763e+003	7.212874e+001
3.054691e+003	7.215920e+001
3.056620e+003	7.210522e+001
3.058548e+003	7.205171e+001
3.060477e+003	7.207042e+001
3.062405e+003	7.204479e+001
3.064333e+003	7.196481e+001
3.066262e+003	7.193166e+001
3.068190e+003	7.193327e+001
3.070119e+003	7.190926e+001
3.072047e+003	7.190189e+001
3.073976e+003	7.193734e+001
3.075904e+003	7.193864e+001
3.077833e+003	7.190320e+001
3.079761e+003	7.190654e+001
3.081690e+003	7.186754e+001
3.083618e+003	7.184284e+001
3.085547e+003	7.181355e+001
3.087475e+003	7.178481e+001
3.089404e+003	7.177866e+001
3.091332e+003	7.177620e+001
3.093260e+003	7.178626e+001
3.095189e+003	7.181007e+001
3.097117e+003	7.177804e+001

3.099046e+003	7.167963e+001
3.100974e+003	7.160227e+001
3.102903e+003	7.158729e+001
3.104831e+003	7.160451e+001
3.106760e+003	7.160091e+001
3.108688e+003	7.157620e+001
3.110617e+003	7.156907e+001
3.112545e+003	7.161801e+001
3.114474e+003	7.161295e+001
3.116402e+003	7.154804e+001
3.118331e+003	7.151333e+001
3.120259e+003	7.149505e+001
3.122188e+003	7.148972e+001
3.124116e+003	7.149109e+001
3.126044e+003	7.148089e+001
3.127973e+003	7.147892e+001
3.129901e+003	7.141524e+001
3.131830e+003	7.138048e+001
3.133758e+003	7.142411e+001
3.135687e+003	7.139429e+001
3.137615e+003	7.131881e+001
3.139544e+003	7.126023e+001
3.141472e+003	7.126975e+001
3.143401e+003	7.129196e+001
3.145329e+003	7.127023e+001
3.147258e+003	7.118514e+001
3.149186e+003	7.114002e+001
3.151115e+003	7.116970e+001
3.153043e+003	7.117722e+001
3.154971e+003	7.119440e+001
3.156900e+003	7.113696e+001
3.158828e+003	7.108934e+001
3.160757e+003	7.111819e+001
3.162685e+003	7.114597e+001
3.164614e+003	7.115439e+001
3.166542e+003	7.107900e+001
3.168471e+003	7.101520e+001
3.170399e+003	7.095392e+001
3.172328e+003	7.094653e+001
3.174256e+003	7.103014e+001
3.176185e+003	7.104449e+001
3.178113e+003	7.096206e+001
3.180042e+003	7.093327e+001
3.181970e+003	7.092487e+001
3.183898e+003	7.089029e+001
3.185827e+003	7.087695e+001
3.187755e+003	7.089094e+001
3.189684e+003	7.090125e+001
3.191612e+003	7.093032e+001
3.193541e+003	7.088817e+001

3.195469e+003	7.079478e+001
3.197398e+003	7.078770e+001
3.199326e+003	7.080037e+001
3.201255e+003	7.072091e+001
3.203183e+003	7.072750e+001
3.205112e+003	7.074626e+001
3.207040e+003	7.069949e+001
3.208969e+003	7.065582e+001
3.210897e+003	7.062310e+001
3.212825e+003	7.064267e+001
3.214754e+003	7.065679e+001
3.216682e+003	7.062154e+001
3.218611e+003	7.068002e+001
3.220539e+003	7.068192e+001
3.222468e+003	7.058233e+001
3.224396e+003	7.057632e+001
3.226325e+003	7.062214e+001
3.228253e+003	7.056398e+001
3.230182e+003	7.051419e+001
3.232110e+003	7.052417e+001
3.234039e+003	7.048428e+001
3.235967e+003	7.048465e+001
3.237896e+003	7.045062e+001
3.239824e+003	7.040161e+001
3.241752e+003	7.037185e+001
3.243681e+003	7.038950e+001
3.245609e+003	7.042377e+001
3.247538e+003	7.043309e+001
3.249466e+003	7.038004e+001
3.251395e+003	7.030178e+001
3.253323e+003	7.030480e+001
3.255252e+003	7.039022e+001
3.257180e+003	7.040216e+001
3.259109e+003	7.031673e+001
3.261037e+003	7.025085e+001
3.262966e+003	7.023482e+001
3.264894e+003	7.031093e+001
3.266823e+003	7.029810e+001
3.268751e+003	7.024705e+001
3.270679e+003	7.025330e+001
3.272608e+003	7.019186e+001
3.274536e+003	7.015221e+001
3.276465e+003	7.021785e+001
3.278393e+003	7.017455e+001
3.280322e+003	7.005711e+001
3.282250e+003	6.999187e+001
3.284179e+003	7.002618e+001
3.286107e+003	7.005511e+001
3.288036e+003	7.000308e+001
3.289964e+003	7.000481e+001

3.291893e+003	7.005025e+001
3.293821e+003	7.003358e+001
3.295750e+003	6.995922e+001
3.297678e+003	6.986203e+001
3.299606e+003	6.978725e+001
3.301535e+003	6.978285e+001
3.303463e+003	6.986868e+001
3.305392e+003	6.983022e+001
3.307320e+003	6.981535e+001
3.309249e+003	6.988770e+001
3.311177e+003	6.986240e+001
3.313106e+003	6.978409e+001
3.315034e+003	6.976585e+001
3.316963e+003	6.975854e+001
3.318891e+003	6.970850e+001
3.320820e+003	6.970152e+001
3.322748e+003	6.970944e+001
3.324677e+003	6.971544e+001
3.326605e+003	6.970201e+001
3.328533e+003	6.967566e+001
3.330462e+003	6.966733e+001
3.332390e+003	6.960582e+001
3.334319e+003	6.953825e+001
3.336247e+003	6.947363e+001
3.338176e+003	6.942166e+001
3.340104e+003	6.947208e+001
3.342033e+003	6.953524e+001
3.343961e+003	6.952100e+001
3.345890e+003	6.949414e+001
3.347818e+003	6.945543e+001
3.349747e+003	6.947679e+001
3.351675e+003	6.952581e+001
3.353604e+003	6.950628e+001
3.355532e+003	6.948397e+001
3.357460e+003	6.945739e+001
3.359389e+003	6.942837e+001
3.361317e+003	6.941875e+001
3.363246e+003	6.938490e+001
3.365174e+003	6.940134e+001
3.367103e+003	6.940784e+001
3.369031e+003	6.938945e+001
3.370960e+003	6.942831e+001
3.372888e+003	6.946737e+001
3.374817e+003	6.947819e+001
3.376745e+003	6.942751e+001
3.378674e+003	6.938590e+001
3.380602e+003	6.940208e+001
3.382531e+003	6.943361e+001
3.384459e+003	6.941314e+001
3.386387e+003	6.946729e+001

3.388316e+003	6.950842e+001
3.390244e+003	6.952609e+001
3.392173e+003	6.958845e+001
3.394101e+003	6.958233e+001
3.396030e+003	6.955648e+001
3.397958e+003	6.957423e+001
3.399887e+003	6.956552e+001
3.401815e+003	6.955280e+001
3.403744e+003	6.953983e+001
3.405672e+003	6.955052e+001
3.407601e+003	6.956778e+001
3.409529e+003	6.960187e+001
3.411458e+003	6.962327e+001
3.413386e+003	6.960763e+001
3.415314e+003	6.958424e+001
3.417243e+003	6.959081e+001
3.419171e+003	6.954887e+001
3.421100e+003	6.949776e+001
3.423028e+003	6.951038e+001
3.424957e+003	6.956131e+001
3.426885e+003	6.952185e+001
3.428814e+003	6.944790e+001
3.430742e+003	6.942154e+001
3.432671e+003	6.941069e+001
3.434599e+003	6.943327e+001
3.436528e+003	6.944643e+001
3.438456e+003	6.943328e+001
3.440385e+003	6.935502e+001
3.442313e+003	6.924983e+001
3.444241e+003	6.929497e+001
3.446170e+003	6.929415e+001
3.448098e+003	6.928336e+001
3.450027e+003	6.936399e+001
3.451955e+003	6.938274e+001
3.453884e+003	6.934813e+001
3.455812e+003	6.930185e+001
3.457741e+003	6.920831e+001
3.459669e+003	6.917363e+001
3.461598e+003	6.920899e+001
3.463526e+003	6.927219e+001
3.465455e+003	6.928239e+001
3.467383e+003	6.929202e+001
3.469312e+003	6.927425e+001
3.471240e+003	6.919664e+001
3.473168e+003	6.923925e+001
3.475097e+003	6.931994e+001
3.477025e+003	6.929375e+001
3.478954e+003	6.923442e+001
3.480882e+003	6.913940e+001
3.482811e+003	6.908842e+001

3.484739e+003	6.916747e+001
3.486668e+003	6.921323e+001
3.488596e+003	6.913206e+001
3.490525e+003	6.910379e+001
3.492453e+003	6.913128e+001
3.494382e+003	6.912290e+001
3.496310e+003	6.911310e+001
3.498239e+003	6.908827e+001
3.500167e+003	6.905162e+001
3.502095e+003	6.899379e+001
3.504024e+003	6.894879e+001
3.505952e+003	6.894157e+001
3.507881e+003	6.892169e+001
3.509809e+003	6.898725e+001
3.511738e+003	6.911695e+001
3.513666e+003	6.907273e+001
3.515595e+003	6.897247e+001
3.517523e+003	6.897058e+001
3.519452e+003	6.899825e+001
3.521380e+003	6.894043e+001
3.523309e+003	6.886903e+001
3.525237e+003	6.886295e+001
3.527166e+003	6.885868e+001
3.529094e+003	6.894585e+001
3.531022e+003	6.898138e+001
3.532951e+003	6.888606e+001
3.534879e+003	6.885374e+001
3.536808e+003	6.887189e+001
3.538736e+003	6.886859e+001
3.540665e+003	6.877991e+001
3.542593e+003	6.876523e+001
3.544522e+003	6.876240e+001
3.546450e+003	6.880583e+001
3.548379e+003	6.879586e+001
3.550307e+003	6.881992e+001
3.552236e+003	6.877451e+001
3.554164e+003	6.871882e+001
3.556093e+003	6.875255e+001
3.558021e+003	6.884633e+001
3.559949e+003	6.883983e+001
3.561878e+003	6.872224e+001
3.563806e+003	6.860155e+001
3.565735e+003	6.860783e+001
3.567663e+003	6.863004e+001
3.569592e+003	6.860272e+001
3.571520e+003	6.863879e+001
3.573449e+003	6.872025e+001
3.575377e+003	6.869362e+001
3.577306e+003	6.868839e+001
3.579234e+003	6.869167e+001

3.581163e+003	6.863899e+001
3.583091e+003	6.855949e+001
3.585020e+003	6.842654e+001
3.586948e+003	6.837136e+001
3.588876e+003	6.846162e+001
3.590805e+003	6.851347e+001
3.592733e+003	6.851318e+001
3.594662e+003	6.850079e+001
3.596590e+003	6.846046e+001
3.598519e+003	6.838117e+001
3.600447e+003	6.843015e+001
3.602376e+003	6.843652e+001
3.604304e+003	6.847867e+001
3.606233e+003	6.843536e+001
3.608161e+003	6.841140e+001
3.610090e+003	6.835845e+001
3.612018e+003	6.838236e+001
3.613947e+003	6.831571e+001
3.615875e+003	6.817616e+001
3.617803e+003	6.796819e+001
3.619732e+003	6.807037e+001
3.621660e+003	6.823391e+001
3.623589e+003	6.822013e+001
3.625517e+003	6.814188e+001
3.627446e+003	6.812815e+001
3.629374e+003	6.806654e+001
3.631303e+003	6.803216e+001
3.633231e+003	6.807757e+001
3.635160e+003	6.814849e+001
3.637088e+003	6.822733e+001
3.639017e+003	6.828092e+001
3.640945e+003	6.827462e+001
3.642874e+003	6.818881e+001
3.644802e+003	6.814429e+001
3.646730e+003	6.806723e+001
3.648659e+003	6.824713e+001
3.650587e+003	6.821719e+001
3.652516e+003	6.801578e+001
3.654444e+003	6.806718e+001
3.656373e+003	6.797845e+001
3.658301e+003	6.804708e+001
3.660230e+003	6.814909e+001
3.662158e+003	6.814911e+001
3.664087e+003	6.812035e+001
3.666015e+003	6.807199e+001
3.667944e+003	6.800662e+001
3.669872e+003	6.789206e+001
3.671801e+003	6.768402e+001
3.673729e+003	6.756733e+001
3.675657e+003	6.754664e+001

3.677586e+003	6.778789e+001
3.679514e+003	6.793648e+001
3.681443e+003	6.784516e+001
3.683371e+003	6.772626e+001
3.685300e+003	6.761495e+001
3.687228e+003	6.767344e+001
3.689157e+003	6.766074e+001
3.691085e+003	6.741647e+001
3.693014e+003	6.740293e+001
3.694942e+003	6.747635e+001
3.696871e+003	6.746198e+001
3.698799e+003	6.756415e+001
3.700728e+003	6.764259e+001
3.702656e+003	6.743733e+001
3.704584e+003	6.738438e+001
3.706513e+003	6.739446e+001
3.708441e+003	6.737416e+001
3.710370e+003	6.739017e+001
3.712298e+003	6.722080e+001
3.714227e+003	6.705026e+001
3.716155e+003	6.705788e+001
3.718084e+003	6.708566e+001
3.720012e+003	6.704221e+001
3.721941e+003	6.698958e+001
3.723869e+003	6.687687e+001
3.725798e+003	6.694925e+001
3.727726e+003	6.698215e+001
3.729655e+003	6.692722e+001
3.731583e+003	6.697138e+001
3.733511e+003	6.682836e+001
3.735440e+003	6.664519e+001
3.737368e+003	6.673022e+001
3.739297e+003	6.670373e+001
3.741225e+003	6.664088e+001
3.743154e+003	6.669578e+001
3.745082e+003	6.672575e+001
3.747011e+003	6.670404e+001
3.748939e+003	6.656669e+001
3.750868e+003	6.652875e+001
3.752796e+003	6.673151e+001
3.754725e+003	6.669048e+001
3.756653e+003	6.661813e+001
3.758582e+003	6.655405e+001
3.760510e+003	6.653100e+001
3.762438e+003	6.653465e+001
3.764367e+003	6.656821e+001
3.766295e+003	6.640469e+001
3.768224e+003	6.646097e+001
3.770152e+003	6.651384e+001
3.772081e+003	6.647073e+001

3.774009e+003	6.641207e+001
3.775938e+003	6.632775e+001
3.777866e+003	6.637120e+001
3.779795e+003	6.642293e+001
3.781723e+003	6.634704e+001
3.783652e+003	6.629578e+001
3.785580e+003	6.622694e+001
3.787509e+003	6.624113e+001
3.789437e+003	6.625610e+001
3.791365e+003	6.623183e+001
3.793294e+003	6.618429e+001
3.795222e+003	6.622993e+001
3.797151e+003	6.602116e+001
3.799079e+003	6.585912e+001
3.801008e+003	6.587730e+001
3.802936e+003	6.588951e+001
3.804865e+003	6.592477e+001
3.806793e+003	6.589524e+001
3.808722e+003	6.584003e+001
3.810650e+003	6.596114e+001
3.812579e+003	6.600796e+001
3.814507e+003	6.587988e+001
3.816436e+003	6.557290e+001
3.818364e+003	6.541261e+001
3.820292e+003	6.554065e+001
3.822221e+003	6.557468e+001
3.824149e+003	6.562156e+001
3.826078e+003	6.564088e+001
3.828006e+003	6.559277e+001
3.829935e+003	6.556991e+001
3.831863e+003	6.544816e+001
3.833792e+003	6.540282e+001
3.835720e+003	6.538181e+001
3.837649e+003	6.532738e+001
3.839577e+003	6.530509e+001
3.841506e+003	6.532508e+001
3.843434e+003	6.536633e+001
3.845363e+003	6.530482e+001
3.847291e+003	6.525259e+001
3.849219e+003	6.516386e+001
3.851148e+003	6.510328e+001
3.853076e+003	6.546694e+001
3.855005e+003	6.526389e+001
3.856933e+003	6.515226e+001
3.858862e+003	6.517635e+001
3.860790e+003	6.516296e+001
3.862719e+003	6.506941e+001
3.864647e+003	6.502645e+001
3.866576e+003	6.492378e+001
3.868504e+003	6.497901e+001

3.870433e+003	6.490869e+001
3.872361e+003	6.489429e+001
3.874290e+003	6.496428e+001
3.876218e+003	6.500848e+001
3.878146e+003	6.502681e+001
3.880075e+003	6.505129e+001
3.882003e+003	6.489296e+001
3.883932e+003	6.491116e+001
3.885860e+003	6.499881e+001
3.887789e+003	6.484098e+001
3.889717e+003	6.475263e+001
3.891646e+003	6.472466e+001
3.893574e+003	6.474589e+001
3.895503e+003	6.470648e+001
3.897431e+003	6.463919e+001
3.899360e+003	6.458073e+001
3.901288e+003	6.456836e+001
3.903217e+003	6.436642e+001
3.905145e+003	6.437651e+001
3.907073e+003	6.456413e+001
3.909002e+003	6.466647e+001
3.910930e+003	6.472945e+001
3.912859e+003	6.468279e+001
3.914787e+003	6.457877e+001
3.916716e+003	6.453232e+001
3.918644e+003	6.452748e+001
3.920573e+003	6.453310e+001
3.922501e+003	6.448993e+001
3.924430e+003	6.437153e+001
3.926358e+003	6.433815e+001
3.928287e+003	6.433866e+001
3.930215e+003	6.433320e+001
3.932144e+003	6.430418e+001
3.934072e+003	6.430745e+001
3.936000e+003	6.432341e+001
3.937929e+003	6.427636e+001
3.939857e+003	6.427699e+001
3.941786e+003	6.430280e+001
3.943714e+003	6.422871e+001
3.945643e+003	6.416328e+001
3.947571e+003	6.413081e+001
3.949500e+003	6.407285e+001
3.951428e+003	6.413719e+001
3.953357e+003	6.420678e+001
3.955285e+003	6.416702e+001
3.957214e+003	6.411954e+001
3.959142e+003	6.410013e+001
3.961071e+003	6.410209e+001
3.962999e+003	6.410789e+001
3.964927e+003	6.416663e+001

3.966856e+003	6.416638e+001
3.968784e+003	6.407645e+001
3.970713e+003	6.402985e+001
3.972641e+003	6.402248e+001
3.974570e+003	6.394360e+001
3.976498e+003	6.391476e+001
3.978427e+003	6.388617e+001
3.980355e+003	6.390150e+001
3.982284e+003	6.395122e+001
3.984212e+003	6.392346e+001
3.986141e+003	6.378749e+001
3.988069e+003	6.371294e+001
3.989998e+003	6.373185e+001
3.991926e+003	6.374173e+001
3.993854e+003	6.371293e+001
3.995783e+003	6.370767e+001
3.997711e+003	6.370755e+001
3.999640e+003	6.370592e+001
4.001568e+003	0.000000e+000
