# Supplementary figures and images for: Synthesis of 1D Bi2O3 nanostructures from hybrid electrospun fibrous mats and their morphology, structure, optical and electrical properties
Source: Sci Rep. 2022 Mar 8;12:4046. doi: 10.1038/s41598-022-07830-z (PMC8904472; doi:10.1038/s41598-022-07830-z)

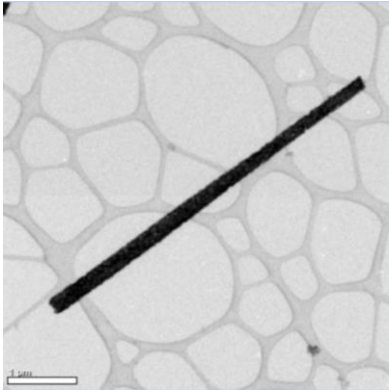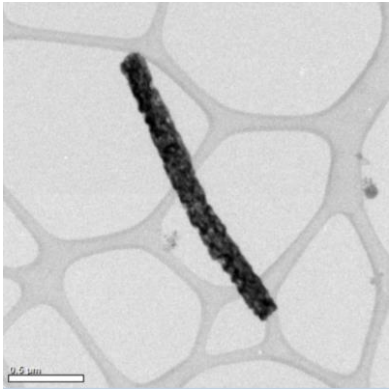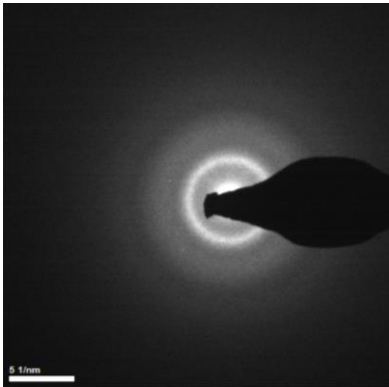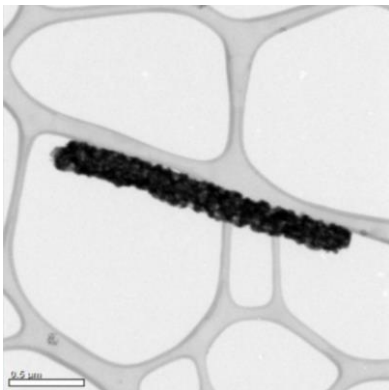

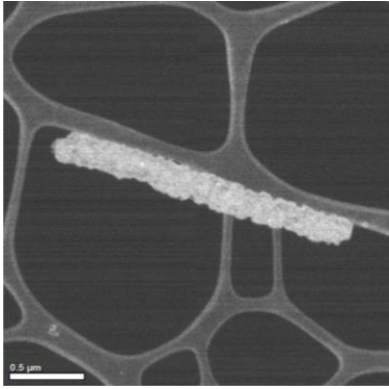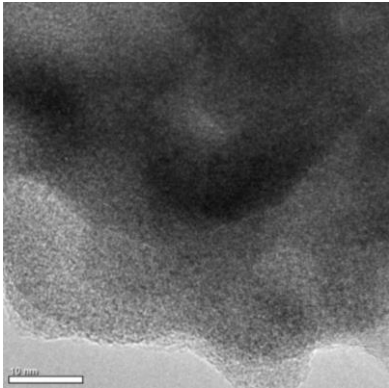

Supplement: Supplementary file 3 — Supplementary Information 3. [file 41598_2022_7830_MOESM3_ESM.pdf]
