# Supplementary material for: Synthesis of 1D Bi2O3 nanostructures from hybrid electrospun fibrous mats and their morphology, structure, optical and electrical properties
Source: Sci Rep. 2022 Mar 8;12:4046. doi: 10.1038/s41598-022-07830-z (PMC8904472; doi:10.1038/s41598-022-07830-z)
Supplement: Supplementary file 4 — Supplementary Information 4. [file 41598_2022_7830_MOESM4_ESM.pdf]

Wavelength (nm)	Absorbance	400˚C
800	0,559454	
798	0,559401	
796	0,559811	
794	0,559346	
792	0,559266	
790	0,559617	
788	0,560172	
786	0,560049	
784	0,561137	
782	0,562111	
780	0,562244	
778	0,563386	
776	0,563598	
774	0,564855	
772	0,564995	
770	0,564559	
768	0,56472	
766	0,56389	
764	0,562565	
762	0,56287	
760	0,563321	
758	0,564119	
756	0,563852	
754	0,565052	
752	0,565503	
750	0,566082	
748	0,566326	
746	0,567004	
744	0,567812	
742	0,568099	
740	0,567844	
738	0,567594	
736	0,567276	
734	0,567821	
732	0,567048	
730	0,567752	
728	0,567672	
726	0,568147	
724	0,569094	
722	0,569444	
720	0,569264	
718	0,569504	
716	0,569551	
714	0,569863	
712	0,571539	
710	0,571772	
708	0,573219	
706	0,572728	
704	0,573131	

702	0,57261
700	0,572023
698	0,571372
696	0,571653
694	0,573202
692	0,575178
690	0,576851
688	0,576733
686	0,575687
684	0,574608
682	0,573838
680	0,572251
678	0,571108
676	0,568963
674	0,568864
672	0,569055
670	0,569526
668	0,568631
666	0,568604
664	0,568591
662	0,570056
660	0,571226
658	0,571113
656	0,571169
654	0,572045
652	0,573085
650	0,57414
648	0,575172
646	0,576156
644	0,577228
642	0,577743
640	0,577495
638	0,57764
636	0,57838
634	0,577491
632	0,576743
630	0,575456
628	0,576082
626	0,576393
624	0,576326
622	0,577768
620	0,579153
618	0,58014
616	0,581769
614	0,58207
612	0,580569
610	0,580486
608	0,580163
606	0,579596
604	0,578282

602	0,578093
600	0,578123
598	0,578092
596	0,578325
594	0,579643
592	0,579947
590	0,580715
588	0,582519
586	0,583975
584	0,58524
582	0,585395
580	0,586462
578	0,585688
576	0,585161
574	0,58397
572	0,582962
570	0,58209
568	0,58283
566	0,583711
564	0,584378
562	0,585714
560	0,587689
558	0,589152
556	0,591714
554	0,593268
552	0,593148
550	0,593032
548	0,592179
546	0,591641
544	0,59088
542	0,590619
540	0,589671
538	0,589163
536	0,587507
534	0,587066
532	0,586338
530	0,585321
528	0,583846
526	0,582644
524	0,582369
522	0,583607
520	0,586285
518	0,588017
516	0,58869
514	0,589664
512	0,59099
510	0,592599
508	0,593804
506	0,595927
504	0,598326

502	0,600039
500	0,602702
498	0,603615
496	0,603662
494	0,60114
492	0,597928
490	0,595433
488	0,592614
486	0,589907
484	0,58822
482	0,586488
480	0,587464
478	0,589089
476	0,592556
474	0,596222
472	0,597869
470	0,598071
468	0,5977
466	0,596975
464	0,596424
462	0,596445
460	0,596398
458	0,596191
456	0,596539
454	0,59761
452	0,598201
450	0,598405
448	0,598113
446	0,598003
444	0,597696
442	0,598135
440	0,599071
438	0,600585
436	0,602991
434	0,604142
432	0,603833
430	0,603194
428	0,603195
426	0,602055
424	0,599834
422	0,598609
420	0,597643
418	0,59764
416	0,599047
414	0,600949
412	0,603922
410	0,605622
408	0,606921
406	0,608419
404	0,609583

402	0,609495
400	0,608793
398	0,608937
396	0,609079
394	0,60854
392	0,607781
390	0,607635
388	0,607778
386	0,607369
384	0,607929
382	0,609121
380	0,608891
378	0,610182
376	0,611509
374	0,613924
372	0,615274
370	0,615872
368	0,616721
366	0,615895
364	0,614226
362	0,612745
360	0,610751
358	0,609938
356	0,610555
354	0,612192
352	0,614821
350	0,618149
348	0,620668
346	0,623032
344	0,625012
342	0,626217
340	0,628223
338	0,632641
336	0,636939
334	0,639951
332	0,643684
330	0,648171
328	0,650018
326	0,657814
324	0,666225
322	0,675572
320	0,685877
318	0,698114
316	0,711144
314	0,724206
312	0,741797
310	0,761558
308	0,784085
306	0,810262
304	0,836089

302	0,863584
300	0,892988
