# Supplementary material for: Synthesis of 1D Bi2O3 nanostructures from hybrid electrospun fibrous mats and their morphology, structure, optical and electrical properties
Source: Sci Rep. 2022 Mar 8;12:4046. doi: 10.1038/s41598-022-07830-z (PMC8904472; doi:10.1038/s41598-022-07830-z)
Supplement: Supplementary file 5 — Supplementary Information 5. [file 41598_2022_7830_MOESM5_ESM.pdf]

Wavelength (nm)	Absorbance	600˚C
800	1,370768	
798	1,367882	
796	1,369036	
794	1,367164	
792	1,367913	
790	1,366196	
788	1,368306	
786	1,369969	
784	1,371638	
782	1,374835	
780	1,376541	
778	1,382388	
776	1,38472	
774	1,386222	
772	1,386984	
770	1,386349	
768	1,385827	
766	1,379788	
764	1,375336	
762	1,373194	
760	1,370687	
758	1,37128	
756	1,374918	
754	1,377249	
752	1,380306	
750	1,381296	
748	1,383927	
746	1,388005	
744	1,388201	
742	1,39077	
740	1,39158	
738	1,390344	
736	1,389547	
734	1,386765	
732	1,384015	
730	1,383149	
728	1,385586	
726	1,387635	
724	1,394309	
722	1,394395	
720	1,394385	
718	1,395942	
716	1,395723	
714	1,395829	
712	1,398315	
710	1,40646	
708	1,414633	
706	1,413949	
704	1,412629	

702	1,409722
700	1,408305
698	1,405726
696	1,403494
694	1,408672
692	1,417649
690	1,429567
688	1,432703
686	1,430234
684	1,421304
682	1,408089
680	1,395925
678	1,383701
676	1,369948
674	1,359538
672	1,356858
670	1,357207
668	1,358093
666	1,359854
664	1,362478
662	1,369172
660	1,374225
658	1,377391
656	1,376735
654	1,377721
652	1,380004
650	1,385923
648	1,389303
646	1,394455
644	1,399017
642	1,40138
640	1,403664
638	1,406163
636	1,406804
634	1,405203
632	1,401649
630	1,394597
628	1,392345
626	1,390784
624	1,389784
622	1,394102
620	1,399996
618	1,411125
616	1,418472
614	1,420095
612	1,419427
610	1,41206
608	1,407838
606	1,401327
604	1,39632

602	1,393162
600	1,389896
598	1,389963
596	1,392035
594	1,394116
592	1,391047
590	1,394591
588	1,401858
586	1,412126
584	1,420454
582	1,426755
580	1,42865
578	1,426857
576	1,421825
574	1,414295
572	1,40725
570	1,398956
568	1,397533
566	1,39709
564	1,40108
562	1,406435
560	1,414172
558	1,426423
556	1,439284
554	1,452308
552	1,46238
550	1,461282
548	1,457066
546	1,450514
544	1,444547
542	1,438913
540	1,435051
538	1,433565
536	1,428193
534	1,422023
532	1,413535
530	1,401552
528	1,390499
526	1,382213
524	1,376724
522	1,381352
520	1,390241
518	1,40497
516	1,410798
514	1,409817
512	1,413454
510	1,419114
508	1,427787
506	1,43577
504	1,447816

502	1,464704
500	1,480255
498	1,491816
496	1,49859
494	1,49282
492	1,476599
490	1,453544
488	1,425494
486	1,4027
484	1,382464
482	1,366814
480	1,360289
478	1,365995
476	1,384543
474	1,404949
472	1,417937
470	1,423045
468	1,422373
466	1,416336
464	1,409507
462	1,403874
460	1,401438
458	1,40182
456	1,402977
454	1,406783
452	1,412882
450	1,417419
448	1,412371
446	1,408309
444	1,404398
442	1,40196
440	1,402657
438	1,409916
436	1,41836
434	1,427455
432	1,43364
430	1,431918
428	1,421782
426	1,40723
424	1,389475
422	1,369192
420	1,355451
418	1,349234
416	1,350524
414	1,36338
412	1,381241
410	1,396234
408	1,404711
406	1,412049
404	1,416831

402	1,419529
400	1,418869
398	1,417948
396	1,417159
394	1,411548
392	1,401965
390	1,39279
388	1,386684
386	1,388278
384	1,388494
382	1,390655
380	1,389271
378	1,390227
376	1,392382
374	1,401041
372	1,412702
370	1,428407
368	1,440319
366	1,441027
364	1,431424
362	1,409364
360	1,393483
358	1,372471
356	1,359409
354	1,363789
352	1,37438
350	1,385065
348	1,395206
346	1,403052
344	1,408985
342	1,407755
340	1,405273
338	1,408613
336	1,418779
334	1,42782
332	1,429481
330	1,429711
328	1,44575
326	1,454193
324	1,467956
322	1,478971
320	1,490953
318	1,506993
316	1,524411
314	1,537228
312	1,547762
310	1,565802
308	1,585482
306	1,614056
304	1,639367

302	1,660199
300	1,681124
